# High-mobility organic mixed conductors with a low synthetic complexity index *via* direct arylation polymerization†

**DOI:** 10.1039/d4sc01430h

**Published:** 2024-04-19

**Authors:** Joost Kimpel, Youngseok Kim, Jesika Asatryan, Jaime Martín, Renee Kroon, Christian Müller

**Affiliations:** a Department of Chemistry and Chemical Engineering, Chalmers University of Technology 412 96 Göteborg Sweden kimpel@chalmers.se christian.muller@chalmers.se; b Universidade da Coruña, Campus Industrial de Ferrol, CITENI Esteiro 15403 Ferrol Spain; c Laboratory of Organic Electronics, Department of Science and Technology, Linköping University Norrköping Sweden; d Wallenberg Initiative Materials Science for Sustainability, Department of Science and Technology, Linköping University Norrköping Sweden

## Abstract

Through direct arylation polymerization, a series of mixed ion-electron conducting polymers with a low synthetic complexity index is synthesized. A thieno[3,2-*b*]thiophene monomer with oligoether side chains is used in direct arylation polymerization together with a wide range of aryl bromides with varying electronic character from electron-donating thiophene to electron-accepting benzothiadiazole. The obtained polymers are less synthetically complex than other mixed ion–electron conducting polymers due to higher yield, fewer synthetic steps and less toxic reagents. Organic electrochemical transistors (OECTs) based on a newly synthesized copolymer comprising thieno[3,2-*b*]thiophene with oligoether side chains and bithiophene exhibit excellent device performance. A high charge-carrier mobility of up to *μ* = 1.8 cm^2^ V^−1^ s^−1^ was observed, obtained by dividing the figure of merit [μC*] from OECT measurements by the volumetric capacitance C* from electrochemical impedance spectroscopy, which reached a value of more than 215 F cm^−3^.

## Abstract 2

The synthesis of conjugated copolymers with glycolated thieno[3,2-*b*]thiophene units by direct arylation polymerization, their synthetic complexity in the context of literature compounds, and their performance in organic electrochemical transistors is described.

## Graphical abstract

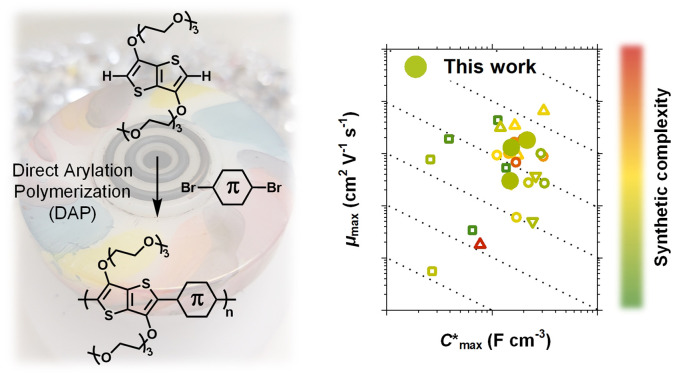

## Introduction

Organic mixed ionic–electronic conductors (OMIECs) are a class of semiconducting materials that are widely used in the field of bioelectronics^1–4^ as well as for energy harvesting^5–7^ and storage.^8,9^ OMIECs can transport both ions and electrons and the charge-carrier density can be modulated through the application of an electrochemical potential, for instance when used as the channel material of organic electrochemical transistors (OECTs), the basic building blocks of more advanced electrochemical circuitry.^10,11^ However, many high-performance materials and devices require cumbersome synthetic protocols and fabrication methods, and hence significant improvements are needed to realize a truly sustainable technology.^12^

**Fig. 1:**
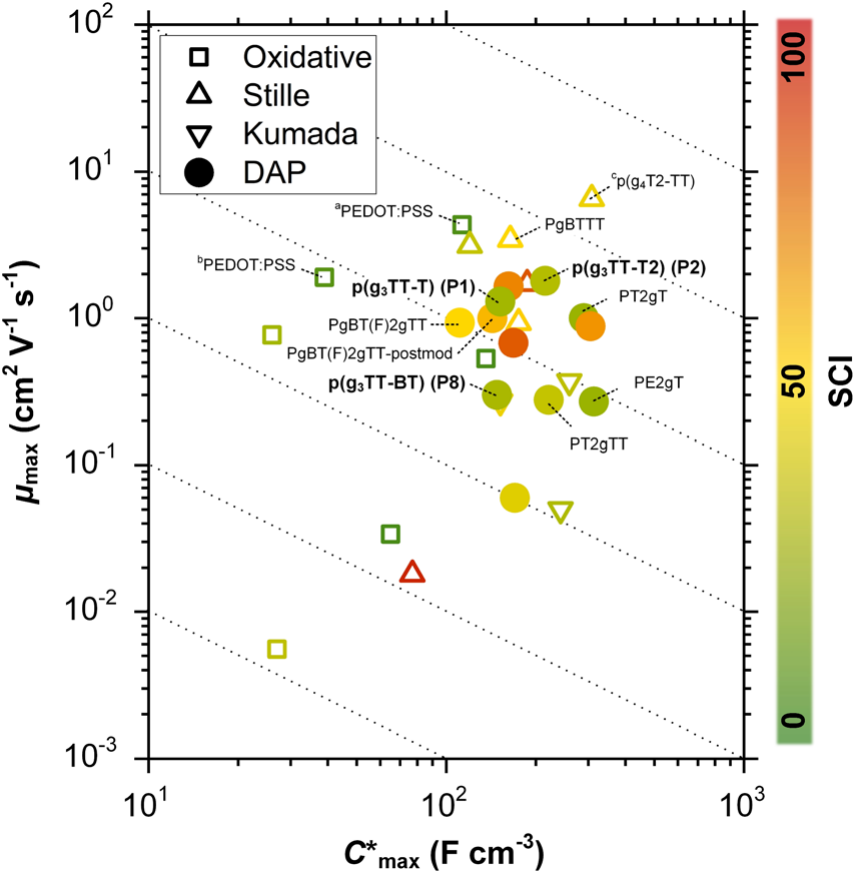
Ashby plot of maximum volumetric capacitance 
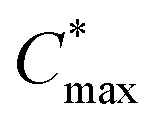
 and maximum charge-carrier mobility *μ*_max_ of previously reported p-type polymers,^15–18,35–37,39–56^ and selected polymers synthesized in this work (bold) by oxidative polymerization (squares), Stille coupling (upward triangles), Kumada coupling (downward triangles) or DAP (circles), with the synthetic complexity index (SCI) of each synthesis indicated by a red-green scale. ^*a*^Poly(3,4-ethylenedioxythiophene):poly(styrene sulfonate) (PEDOT:PSS) post-treated with sulfuric acid,^17^^*b*^PEDOT:PSS post-treated with ethylene glycol,^15^^*c*^p(g_4_T2-TT) (see Fig. S1† for chemical structure) fractionated by size-exclusion chromatography (highest molecular weight fraction, catalyst removed).^16^ See Fig. S2† for the Ashby plot of SCI *vs.* [*μ*C*]_max_.

Some of the most promising types of OMIECs are conjugated polymers with oligoether side chains. The conjugated backbone can conduct electronic charge while the oligoether side chains result in a high ionic mobility and facilitate the ingression of counterions from the electrolyte.^13,14^ As a result, many conjugated polymers with oligoether side chains feature both a high charge-carrier mobility *μ* as well as a high volumetric capacitance C^*^ (see Fig. 1), two parameters that are often used to compare OMIECs.^15^ Among p-type materials, thieno[3,2-*b*]thiophene-based copolymers can exhibit outstanding electrochemical performance, *e.g.* a [μC*] value as large as 10^2^ to 10^3^ F cm^−1^ V^−1^ s^−1^,^16^ which in a few cases exceeds that of poly(3,4-ethylenedioxythiophene):poly(styrene sulfonate) (PEDOT:PSS, see Fig. 1).^17,18^

Thieno[3,2-*b*]thiophene-based copolymers, and more generally many state-of-the-art OMIEC materials, are synthesized *via* cross coupling polycondensation reactions between aryl halides and other reactive monomers. The most widely used cross coupling polycondensation reactions such as Stille coupling (organotin), Suzuki coupling (organoboron) and Kumada coupling (organomagnesium) share a common drawback: one of the monomers must be functionalized to include the reactive group that facilitates the polymerization. Besides adding a synthetic step, the functionalization makes precursors toxic (Stille), atom inefficient (Suzuki), or pyrophoric and difficult to handle (Kumada). As a result, OMIECs made by these techniques feature a relatively high synthetic complexity index (SCI). The SCI is a factor that assesses the accessibility of a compound by considering a variety of parameters including the number of synthetic steps, the yield, the number of work-up operations and the hazards involved, and should therefore be kept low.^19^ The SCI is a value relative to the highest SCI in the set of compounds that is compared.

An alternative synthesis route is direct arylation polymerization (DAP), a polycondensation method that uses a palladium catalyst and various additives to couple unfunctionalized aromatics and aryl halides yielding conjugated polymers.^20–22^ Avoidance of functionalization leads to a better atom economy as well as less toxic reactive species and side products. This all results in a lower SCI.

Despite the clear advantages of DAP, the resulting materials often underperform compared to other conventional routes. Compared to Stille-made polymers, direct arylation polymers often have a lower molecular weight^23^ and a greater prevalence of defects.^24^ Homocoupling defects are defined by subsequent monomers in the chain repeating themselves. This is caused by aryl nucleophiles (Ar–H) and aryl electrophiles (Ar–Br) in DAP being much closer in reactivity. Accordingly, the C–H bond must be sufficiently active to undergo reaction and prevent homocoupling of the dibrominated monomer – a side reaction also seen in Stille and Suzuki coupling despite highly orthogonal reactivity of the monomers in those polymerization reactions.^25,26^ Generally, homocoupling defects somewhat limit the synthesis of high molecular-weight polymers as per Carothers' equation. Branching defects, *i.e.* β-defects, are more detrimental to the device properties of the final material and are an explicit class of defects that can arise when DAP is used. Branching occurs when an unintended aryl C–H is activated on the monomer. Activation of β-protons can cause cross-conjugation in the polymer as well as poor π-stacking due to crowding of polymer chains. For instance, an increased branching content in a diketopyrrolopyrrole (DPP) based copolymer led to a significant decrease in photovoltaic device efficiency due to poor π-stacking.^27^ Instead, naphthalenediimide (NDI) based copolymers made with controlled defect-free DAP to minimize branching defects showed more reproducible results in field effect transistors.^28,29^ In some cases, β-defects led to crosslinking and thus less soluble polymers.^30^

One strategy to avoid coupling defects is through judicious monomer design. Homocoupling can be mostly circumvented by using aromatic units with low C–H bond dissociation energies. This can be achieved by making the aromatic unit electron-poor, in the case of 1,2,4,5-tetrafluorobenzene, or electron rich, in the case of 3,4-ethylenedioxythiophene (EDOT).^31,32^ Occurrence of β-defects can be completely ruled out by substituting all aryl protons except the desired reaction sites. This design principle has been employed in case of direct arylation between EDOT and 2,7-dibromo-9,9-dioctylfluorene, as well as direct arylation between EDOT and dibromo-EDOT analogues, yielding polymers without any branching defects.^33,34^ The possibility of defect formation shrinks the pool of monomers for use in DAP. Even so, with all these design principles in mind, a highly active monomer, which circumvents homocoupling and branching, can lead to successful DAP and high-performance polymers.

Since some of the best performing OMIEC materials comprise thieno[3,2-*b*]thiophene units,^16^ 3,6-bis(triethylene glycol monomethyl ether)thieno[3,2-*b*]thiophene (g_3_TT), which only features two active C–H bonds, is an attractive monomer for DAP. The oligoether chains block positions that could have led to β-defects. Ding *et al.* have polymerized g_3_TT in combination with a fluorinated bis(thiophenyl)benzothiadiazole to obtain the polymer PgBT(F)2gTT (see Fig. S1† for chemical structure), albeit only achieving a low number-average molecular weight of *M*_*n*_ ≈ 4 kg mol^−1^ and a relatively low figure-of-merit of [*μ*C*] = 103 F cm^−1^ V^−1^ s^−1^, which has recently been improved to 145 F cm^−1^ V^−1^ s^−1^ through post-polymerization modification (see Fig. 1 and S1†).^35,36^ In another recent study g_3_TT was paired with thiophene, resulting in a *M*_*n*_ ≈ 11 kg mol^−1^ and a low figure-of-merit of [*μ*C*] = 61 F cm^−1^ V^−1^ s^−1^.^37^ In case of a polythiophene with oligoether side chains, prepared by Stille coupling, intermediate values of *M*_*n*_ ≈ 20 kg mol^−1^ yield an optimal OECT performance and thus it can be anticipated that higher [*μ*C*] values can be achieved if g_3_TT is paired with other comonomers that enable a higher degree of polymerization.^38^

In this work, the versatility of g_3_TT monomers for DAP is explored. We demonstrate that g_3_TT can be combined with a wide range of common conjugated comonomers, including electron-rich, electron-deficient, and neutral comonomers such as thiophene (T), benzothiadiazole (BT), and fluorene (F), respectively. Gratifyingly, the makeup of g_3_TT-copolymers allows for absolute molecular weight determination through high-temperature NMR. The most promising copolymer, p(g_3_TT-T2), gave rise to a state-of-the-art OECT performance with [*μ*C*] = 370 F cm^−1^ V^−1^ s^−1^. Importantly, the here described polymers are associated with a lower SCI than other OMIEC materials made by DAP or Stille coupling, which can be attributed to fewer synthetic steps and less toxic reagents (see Fig. 1; see Table S1, Fig. S1† for considered chemical structures, Fig. S2† for SCI *vs.* [*μ*C*]_max_, and Fig. S3–S28† for SCI calculations).

## Results and discussion

### Synthesis

**Fig. 2:**
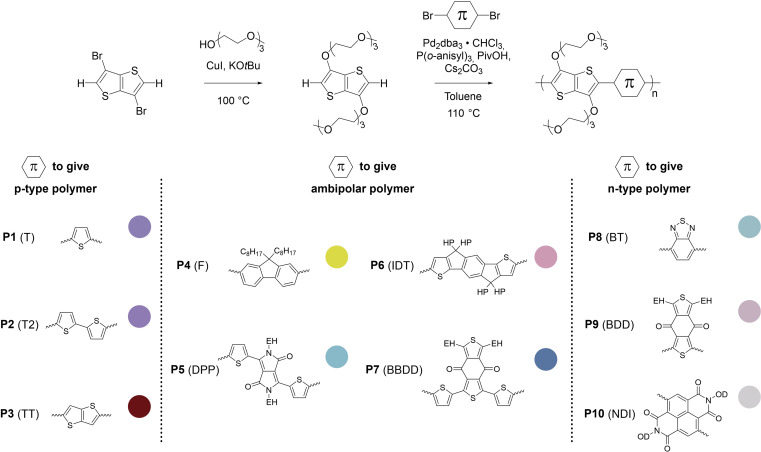
Synthesis of g_3_TT and direct arylation polymerization (DAP) of g_3_TT with comonomers that tend to give polymers with p-type, ambipolar or n-type character. The insets show colors extracted from photographs of thin films of the polymers P1–P10. EH = 2-ethylhexyl, HP = 4-hexylphenyl, OD = 2-octyldodecyl.

3,6-Bis(triethylene glycol monomethyl ether)thieno[3,2-*b*]thiophene (g_3_TT) was synthesized by Ullmann-type coupling of triethylene glycol monomethyl ether to 3,6-dibromothieno[3,2-*b*]thiophene (see Fig. 2). To circumvent the use of harmful solvents and to improve the yield, we attempted the reaction using triethylene glycol monomethyl ether as the reaction medium, *i.e.* the compound acted as both the solvent and reagent. Coupling of the glycol chain to the aromatic unit was achieved by using copper(i)-catalyzed nucleophilic aromatic substitution. Commonly, the oligoether chain is deprotonated using a strong base, *e.g.* potassium *tert*-butoxide (KO*t*Bu), to make it more nucleophilic. We explored the use of sodium hydroxide and cesium hydroxide monohydrate instead of highly reactive and highly hygroscopic KO*t*Bu. Ullmann couplings also benefit from the inclusion of amine ligands to stabilize the copper(i) species. Hence, alongside the solvent and base change we investigated a variety of amine additives (see Fig. S29, Table S3 and ESI Section S2.3† for synthetic details).^57,58^ Ultimately, the use of KO*t*Bu as a base and glycol as the reaction medium significantly increased the final yield of g_3_TT to around 50%, more closely resembling the efficiency of Ullmann coupling for thiophene.^46^ Any changes to this protocol were either detrimental (addition of amine ligand and change of base) or did not improve this initial method (increasing the amount of KO*t*Bu). After a full work-up including extraction, flash column chromatography and recrystallization, g_3_TT was obtained. The final g_3_TT product formed crystals –indicative of the high purity of the compound– and its structure was confirmed with NMR techniques and single crystal X-ray diffraction (see Fig. S30–S35†).

DAP of g_3_TT and a variety of common brominated comonomers was performed. The chosen comonomers ranged from units that generally result in p-type polymers (thiophene (T), bithiophene (T2), and thieno[3,2-*b*]thiophene (TT)), to units that generally result in ambipolar polymers (fluorene (F), diketopyrrolopyrrole (DPP), indacenodithiophene (IDT), and bisthiophenylbenzodithiophenedione (BBDD)), as well as units that generally result in n-type polymers (benzothiadiazole (BT), benzodithiophenedione (BDD), and naphthalenediimide (NDI)), yielding polymers P1–P10 which featured a wide range of colors (see Fig. 2 and ESI Section S2.4† for synthetic details). Final polymers were obtained by precipitating the reaction mixtures into hexane, treating the crude with a palladium scavenging agent, and subsequently purifying the re-precipitated solids by Soxhlet extraction (Table 1).

**Table 1:** Summary of polymer synthesis

Polymer	Total yield^a^ (%)	Yield^b^ (%)	*M*_*n*,SEC_ c (kg mol^−1^)	*Đ* c	*M*_*n*,NMR_ d (kg mol^−1^)
P1 (T)	87	48	14	6.4	14 ± 0.4
P2 (T2)	93	28	29	2.2	39 ± 6
P3 (TT)	77	38	5	>10	3 ± 0.1
P4 (F)	97	31	34	1.7	49 ± 6
P5 (DPP)	73	17	Oligomeric^f^	n.a.^f^	Oligomeric^f^
P6 (IDT)	96	27	9	1.4	29 ± 3
P7 (BBDD)	84	50	15	6.3	16 ± 2
P8 (BT)	84	43	n.a.^e^	n.a.^e^	13 ± 0.2
P9 (BDD)	82	67	90	1.3	21 ± 1
P10 (NDI)	98	94	oligomeric^f^	n.a.^f^	Oligomeric^f^

a Yield of polymer before Soxhlet extraction relative to the loading of monomers and theoretical yield.

b Yield of highest molecular weight fraction from Soxhlet extraction (for P1–P3 and P8, an average yield from multiple syntheses is given, see Table S5), relative to the loading of monomers and theoretical yield.

c From the materials post-Soxhlet, measured by SEC at 70 °C against poly(methyl methacrylate) (PMMA) standards using dimethylformamide (DMF) with 0.1 wt% LiBr as the eluent.

d From the materials post-Soxhlet, determined by end group analysis of NMR spectra recorded at 120 °C in C_2_D_2_Cl_4_ using a Bruker Avance NEO 600 spectrometer. Error determined by adding signal-to-noise of main chain and end-group in quadrature (Section S3.2).

e Polymer did not dissolve in DMF with 0.1 wt% LiBr.

f Polymers elute later than PMMA calibration suggesting oligomers, confirmed by room temperature NMR using a Bruker Avance NEO 600 spectrometer.

By size-exclusion chromatography (SEC), we determined the number-average molecular weight *M*_*n*,SEC_ with a Polargel column using dimethylformamide (DMF) with 0.1 wt% LiBr as the eluent (see Fig. S36†). The polar column was necessary due to the high polarity of the oligoether side chains. A range of polymer molecular weights was obtained using DAP under the aforementioned conditions; oligomers for P5 (DPP) and P10 (NDI), *M*_*n*,SEC_ <10 kg mol^−1^ for P3 (TT) and P6 (IDT), and *M*_*n*,SEC_ >10 kg mol^−1^ for the other polymers reaching up to 91 kg mol^−1^ for P9 (BDD). Aggregation peaks in chromatograms of P2 (T2), P3 (TT), and P7 (BBDD) suggest that the polymers are only partially soluble. In those cases, only the molecular weight of the soluble fraction could be calculated. We suspect that P9 also aggregates, though its aggregates passed through the filter, which would result in an overestimate of the molecular weight and thus a high *M*_*n*,SEC_. In case of P8 (BT) the chromatogram could not be recorded due to the insolubility of the polymer in DMF with 0.1 wt% LiBr. Note that the reported chromatograms were recorded with relative calibration using PMMA as a standard and thus the here quoted values must not be taken as absolute.

**Fig. 3:**
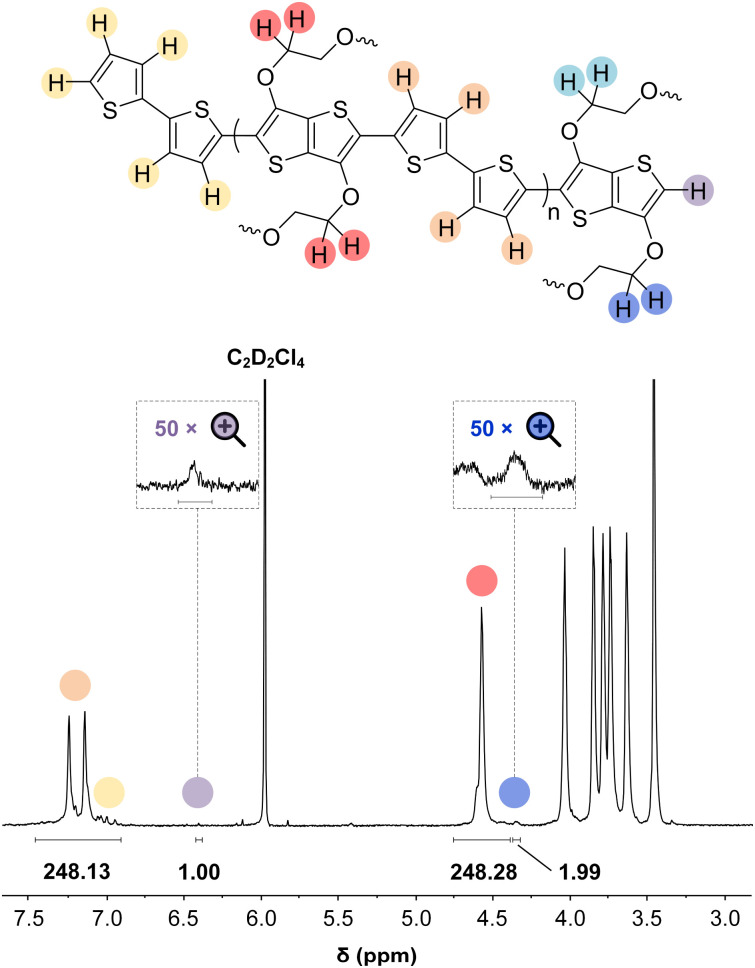
NMR spectrum of p(g_3_TT-T2) (P2 (T2)) recorded in tetrachloroethane-d_2_ at 120 °C, allowing end-group analysis by comparing the end-group oligoether CH_2_ (blue) and the main chain oligoether CH_2_ (red). The signals at circa 6.40 ppm and circa 4.30 are assigned to the aromatic C–H of g_3_TT (purple) and the end-group oligoether CH_2_ (blue), respectively, because the ratio of the integrated areas is 1 : 2.

In some specific cases, end groups of polymers can confidently be assigned by high-temperature NMR.^59,60^ Then, by comparing the end-group signal with the main-chain signal, the absolute number-average molecular weight can be determined by NMR, *M*_*n*,NMR_. High-temperature NMR spectra were recorded by dissolving the polymers in tetrachloroethane-d_2_ at 120 °C using a 600 MHz spectrometer (see Fig. S37–S44†). All polymers display two unique signals indicative of g_3_TT end groups, *i.e.* a peak assigned to the aromatic C–H (*ca.* 6.40 ppm, purple in Fig. 3) and a peak assigned to CH_2_ from the glycol chains closest to the thieno[3,2-*b*]thiophene unit (*ca.* 4.30 ppm, blue in Fig. 3). The aromatic C–H is a clear end-group signal owing to g_3_TT only possessing two aromatic C–H, which are consumed during the cross-coupling reaction. The oligoether signal is assigned to the end group since the ratio between the integrals of the aromatic C–H and the oligoether CH_2_ end group signal lies between 1 : 1.99 and 1 : 2.15 (around 1 : 2) for all polymers. For P7–P9, the other oligoether CH_2_ peak from the end group is also visible (*ca.* 4.55 ppm in Fig. S42–S44,† turquoise in Fig. 3). In all other cases, this end-group peak has merged with the oligoether CH_2_ peak assigned to the polymer repeat unit. By comparing the CH_2_ oligoether end-group signals (*ca.* 4.30 ppm, blue in Fig. 3) to the CH_2_ oligoether main-chain signal (*ca.* 4.60–4.70 ppm, red in Fig. 3), *M*_*n*,NMR_ can be assigned. The end group glycol signal is more shielded than the main-chain glycol signal, also observed for the alkyl chain end group and main chain comparison in case of extensively studied poly(3-hexylthiophene) (P3HT).^59,61^ MALDI-ToF of P2 confirmed debromination of bithiophene end groups (Fig. S45†). All bromines were ultimately replaced with a hydrogen, commonly observed in case of direct arylation polymerization.^62^ Since the synthesis of all polymers is comparable, we argue that none of the polymers P1–4 and P6–P9 possess any residual bromine end groups. Assuming a statistical mixture of end groups of the two monomers, this allowed for the determination of *M*_*n*,NMR_ according to:1
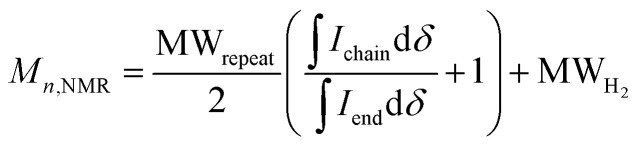
where 
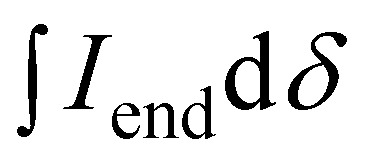
 is the integral of the peak assigned to the oligoether CH_2_ of the end-group side chain that is oriented to the end of the polymer chain (*ca.* 4.30 ppm), 
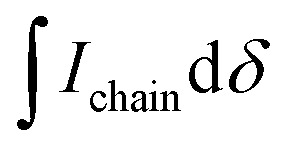
 is the integral of the main-chain oligoether CH_2_ signal assigned to the g_3_TT repeat unit (*ca.* 4.60–4.70 ppm), MW_repeat_ is the molecular weight of the repeat unit, and MW_H_2__ is the molecular weight of the hydrogens at the ends of the polymer chain (see ESI Section S3.1† for derivation of eqn (1)).

By comparing the aromatic proton signals of the comonomer (>7.00 ppm) and the methylene proton signal of the glycol chain closest to the aromatic system of g_3_TT in the main chain (Ar–O–CH_2_–, 4.25–4.70 ppm), the relative incorporation of comonomer and g_3_TT could be determined. The well-matching integrals of the peaks suggest that the comonomers and g3TT are incorporated by the same amount. A maximum deviation of 3% in repeat unit integral is observed, favoring additional g_3_TT incorporation compared to the comonomer. From this, we expect that the homocoupling amount is minimal.

No clear trend in molecular weight could be linked to the electronic character of the comonomers. The lower molecular weights can be explained in some cases: (i) P3 (TT) is highly planar and one-dimensional causing strong aggregation, which possibly causes precipitation during the polymerization, as observed by blockage in the pipette upon dilution with chloroform, (ii) for P5 (DPP) the brominated DPP comonomer may suffer from activated protons leading to β-defects, as observed in an experimental and computational study on DPP reactivity by Bura *et al.*,^63^ which would then cause a monomer imbalance (excess of C–H), and (iii) P10 (NDI) can be expected to experience strong transannular strain between the oligoether side chain from g_3_TT and the aromatic proton or the carbonyl on NDI.

The SCI of the newly synthesized polymers was compared against values for previously reported polymers (see Fig. 1 and ESI Section S1† for SCI calculations).^19^ A lower SCI value indicates a more benign and scalable synthesis. For the polymers in this work, the average yield was used for the SCI calculation in case the material was synthesized multiple times. When considering polymers made by polycondensation, the polymers synthesized in this work feature lower SCI values of 29 to 32 compared to previously reported g_3_TT based polymers made by DAP with a SCI = 35–57 (see Table S1†),^35–37^ thiophene-based polymers with oligoether side chains made by Stille coupling with a SCI = 36 to 93 (see Table S1†),^41,46,64^ and even lower than commercially available poly[2,5-bis(3-tetradecylthiophen-2-yl)thieno[3,2-*b*]thiophene] (PBTTT) with a SCI = 36, also made by Stille coupling (see Table S1†).^49^ The only material with a similar SCI of 26 is a recently reported polythiophene comprising 3,4-bisoligoether thiophene made by DAP.^37^ Generally, higher yield, fewer synthetic steps and fewer hazardous starting materials are the main reasons for the here described low SCI. This is illustrated by calculating the SCI that would be obtained if PgBT(F)2gTT, PgBT(F)2gTT-postmod and PT2gTT (see Fig. 1 and ESI S4 and S8†) were instead prepared using g3TT synthesized as described here. The SCI is then reduced by five points.

One concern associated with an SCI analysis is the variation in reaction yields and accordingly the error in the SCI. To this end, we calculated the error in SCI based on a comparison of repeated syntheses (Table S3†). The monomer synthesis to g_3_TT was repeated a dozen times, and selected polymers were resynthesized several times (Tables S4 and S5†). Through this effort, a range of SCI errors was found between about 2 and 4. For instance, the SCI of P2 (T2) of 32 ± 1.5 and the SCI of P8 (BT) is 29 ± 3.8. In other words, the repeated SCI values are only slightly affected by variations in yield.

In some cases, synthetic steps can be skipped since the intermediate is commercially available (Section S1.2†). By considering commercially available intermediates as a new starting point in the synthesis, a ‘commercially available’ SCI (SCI_comm.avail._) can be calculated. This is the case for seven syntheses in the SCI range 50–93, and PBTTT with an SCI of 37. On average, this decreases the SCI by 13 points, which supports the inclusion of SCI_comm.avail._ in future SCI endeavors.

### Thin-film nanostructure

**Fig. 4:**
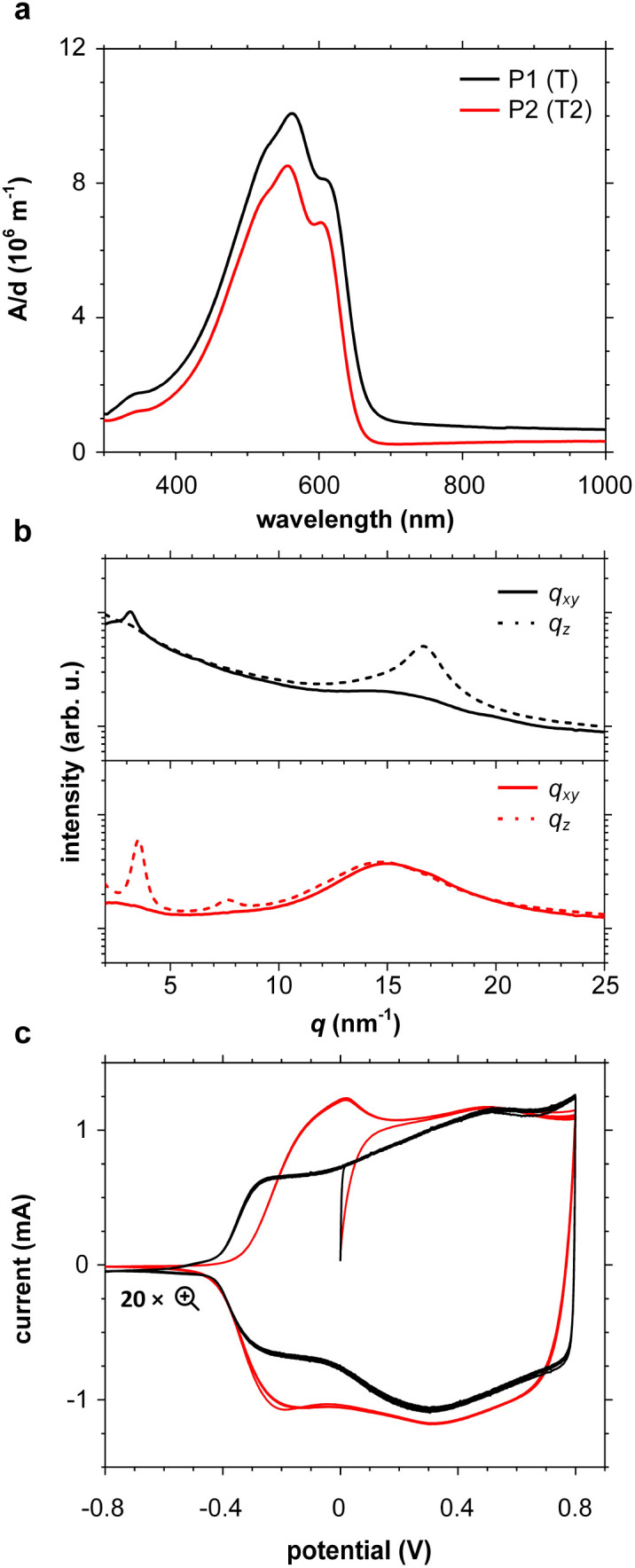
(a) Thickness-normalized UV-vis absorption spectra (absorbance *A* divided by film thickness *d*), (b) GIWAXS in-plane diffractograms (*q*_*xy*_; solid lines) and out-of-plane (*q*_*z*_; dashed lines), and (c) cyclic voltammograms measured in 0.1 M NBu_4_PF_6_ in acetonitrile of thin films of polymers P1 (black) and P2 (red).

UV-vis absorption spectra of thin films of all polymers P1–P10 spin-coated from chloroform solutions feature strong absorption bands in the visible region (see Fig. 4a and S46; see Table S6† for optical bandgaps). Some polymers such as P1 and P2 feature vibronic peaks around 600 nm, which in case of PBTTT indicate the presence of ordered domains.^65,66^ Grazing-incidence wide-angle X-ray scattering (GIWAXS) was performed to examine the solid-state structure of polymer films spin-coated from chloroform solutions in more detail (see Fig. 4b and S47, Table S7†). Diffractograms of polymers P2–P4, P6 and P9 feature a broad amorphous halo at *q* = 14.8 to 15.0 nm^−1^ (*d* = 0.42 to 0.43 nm), while P1, P7 and P8 feature a π-stacking peak at *q*_010_ = 16.7 to 16.8 nm^−1^ (*d*_010_ = 0.36 to 0.38 nm), indicating the presence of ordered domains. The amorphous halo observed in case of several polymers (P2–P4 and P6) also entails a shoulder at higher *q* values, which suggests that there is a minor degree of π-stacking also for these polymers. For polymers P1, P2 and P7–P9 diffractograms also feature lamellar stacking peaks at *q*_100_ of 2.87 to 3.55 nm^−1^ (*d*_100_ = 1.8 to 2.2 nm), respectively, confirming the presence of ordered domains. A coherence length of lamellar stacking of up to 10.4 nm in case of P2 (note that a second order diffraction at *q*_200_ = 7.61 nm^−1^ is also observed) suggests that ordered domains can reach a significant size. Nevertheless, differential scanning calorimetry (DSC) thermograms did not reveal any endothermic melting peaks (data not shown), suggesting that only a small fraction of the polymer films is composed of ordered domains. Degradation during the DSC measurements was ruled out by thermogravimetric analysis (TGA; see Fig. S48†).

### Electrochemical properties

Cyclic voltammetry (CV) was used to determine the oxidation onset potentials *E*_ox_ of thin films of polymers P1–P10 spin-coated from chloroform solutions, using in all cases an acetonitrile-based electrolyte (0.1 M NBu_4_PF_6_ in acetonitrile, AcN, see Fig. 4c and S47†), and for some polymers also an aqueous electrolyte (0.1 M NaCl in H_2_O, see Fig. S49 and Table S6†). Polymers with small, simple comonomers, *i.e.*P1 (T), P2 (T2), P3 (TT) and P8 (BT), feature a low *E*_ox_ of 4.7 eV to 4.8 eV *vs.* ferrocene/ferrocenium (Fc/Fc^+^) in AcN, which can be attributed to the electron-rich g_3_TT core. Interestingly, the electron-withdrawing character of BT does not seem to influence *E*_ox_. Larger comonomers exert a greater effect on *E*_ox_, *e.g. E*_ox_ = 5.6 eV in case of P4 (F), which is similar to values reported for polyfluorene,^67^ and in case of P9 (BDD) the electron-withdrawing ketones in BDD increase *E*_ox_ to 5.7 eV. Oxidation of polymers P1–P5 and P7–P9 was accompanied by a change in color to grey, and the appearance of the neat polymer was recovered upon subsequent reduction, in agreement with the reversibility of CV measurements (see Fig. S50†). Only the polymers P1 (T), P2 (T2), P3 (TT) and P8 (BT), which do not carry alkyl side chains on the comonomer, showed a notable response when carrying out CV with an aqueous electrolyte (see Fig. S51†). Note that all other polymers P4–7, P9 and 10 possess a mixture of oligoether and alkyl side chains. We argue that the more polar nature of polymers P1–3 and P8 facilitates the uptake of ions from an aqueous electrolyte – the oligoether side chains of P1–3 and P8 constitute over 50% of the total mass, but less than 40% in case of the other polymers.

### OECT device characterization

**Fig. 5:**
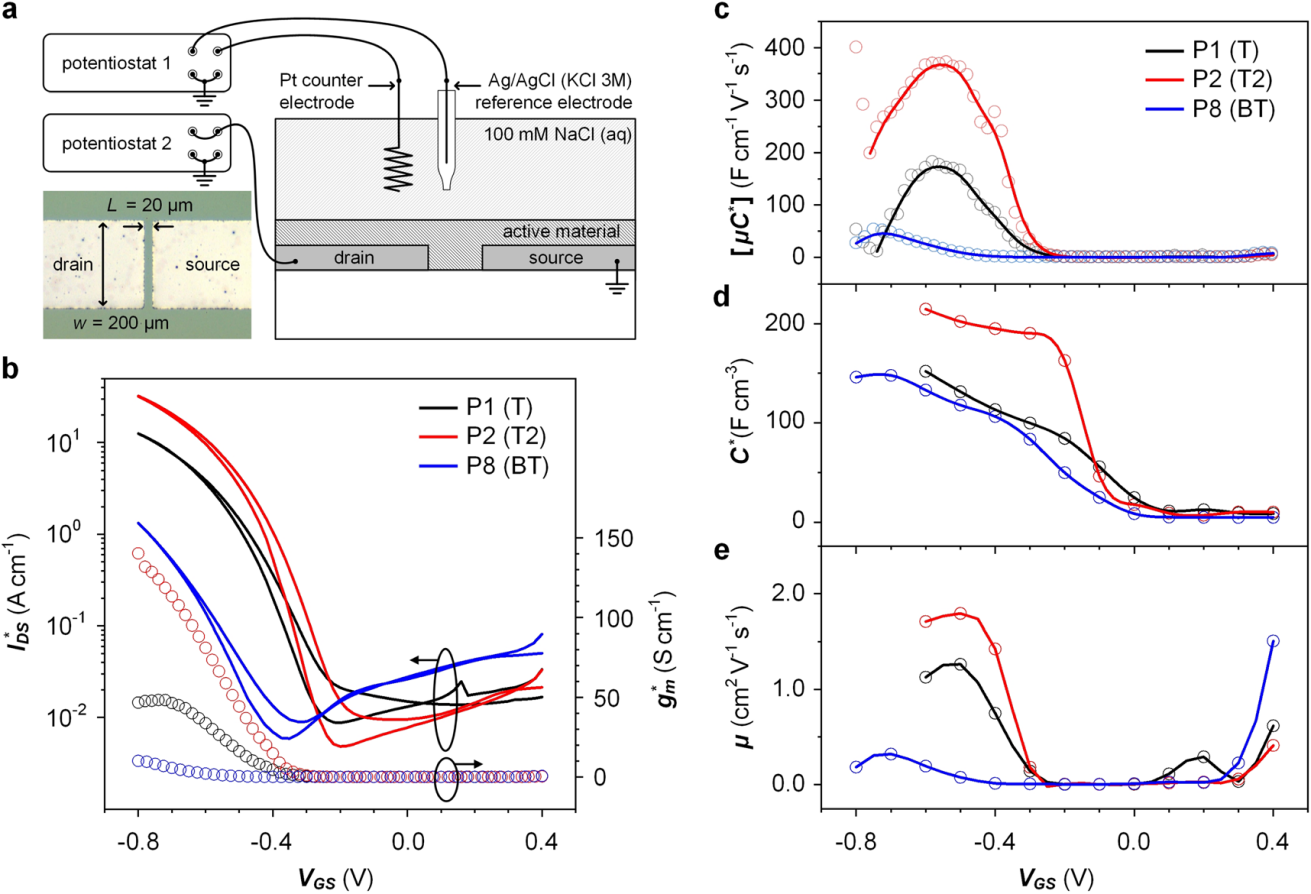
Ionic/electronic properties of conjugated polymers. (a) Schematics of OECT device characterizations. (b) Transfer curves and corresponding normalized transconductance, 
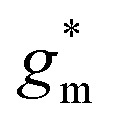
, as a function of gate potential, *V*_GS_. Drain current, *I*_DS_, and *g*_m_ were normalized by the channel dimensions (*i.e.*, 
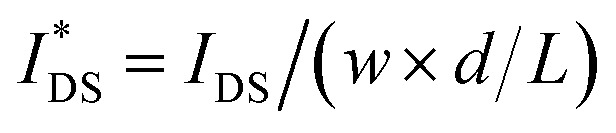
 and 
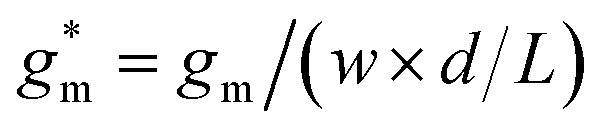
). The characterization was conducted with a backward scan of *V*_GS_ from −0.8 to +0.4 V at a constant *V*_DS_ of −0.6 V. (c–e) Plots of electrical/electrochemical properties as a function of gate potential; (c) product of carrier mobility and volumetric capacitance [μC*] obtained from transfer curves. (d) Volumetric capacitance C* from EIS. (e) Carrier mobility *μ* calculated by dividing [μC*] by C*. Trend lines (solid line) estimated using spline interpolation of measured values (open symbols).

OECTs were prepared by spin-coating the active layer from chloroform solutions on pre-patterned substrates with source-drain metal electrodes. A 0.1 M NaCl aqueous electrolyte was used, and devices comprised a three-electrode configuration with an Ag/AgCl reference electrode and a Pt counter electrode (see Fig. 5a for device architecture). At first, output characteristics were obtained (see Fig. S52†) to determine the type of response (n/p-type) and device operation regime of P1–P3 and P8, which are the only polymers that respond to an aqueous electrolyte (as per CV measurements) owing to the large oligoether side-chain fraction. Devices based on polymers P1, P2 and P8 showed enhancement mode p-type operation with a near zero source-drain current *I*_DS_ at a gate potential *V*_GS_ = 0 V and increased *I*_DS_ at negative *V*_GS_. Noteworthy is that P8 shows p-type behavior despite the n-type character of BT, which is attributed to the strong p-type character of g_3_TT. The response of devices based on P3 was limited (not shown), likely because of the low molecular weight of the polymer and thus the material was not studied further. The polymers P1, P2 and P8 exhibited a negligible contact resistance between the active-layer film and the source/drain Au electrodes. Moreover, OECTs based on P1, P2 and P8 could operate in the saturation regime with a uniform *I*_DS_ at a *V*_DS_ < −0.5 V, and hence devices based on these three polymers were chosen for an in-depth analysis.

Devices based on P2 feature the highest dimension-normalized drain current 
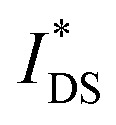
 and dimension-normalized transconductance 
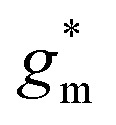
 of 32 A cm^−1^ and 140 S cm^−1^, followed by P1 and P8 with 
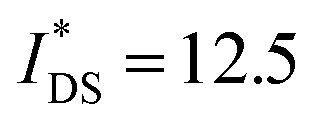
 and 1.3 A cm^−1^, and 
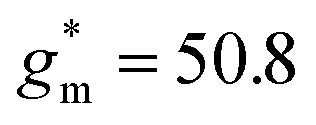
 and 10.3 S cm^−1^, respectively. For devices based on polymers P1 and P2 we observed a similar threshold voltage *V*_th_ of −0.38 V and −0.35 V respectively, while devices based on P8 had a lower *V*_th_ of −0.53 V (see Table 2). The [μC*] values (see Fig. 5c) were extracted according to:

2 ∂*g*_m_/∂*V*_GS_ = [μC*]·(*wd*/*L*)

where *w* and *L* are the width and length of the device channel and *d* is the active-layer thickness. P8 reached a relatively low [μC*]_max_ = 53 F cm^−1^ V^−1^ s^−1^ while for P1 and P2 we obtained high values of 182 and 370 F cm^−1^ V^−1^ s^−1^, respectively (seeTable 2).

**Table 2:** CV and OECT performance. Oxidation onset potential *E*_ox,H_2_O_ measured against Ag/AgCl in 0.1 M NaCl in H_2_O from −0.4 to 0.6 V and *E*_ox,AcN_ measured against Fc/Fc^+^ in 0.1 M NBu_4_PF_6_ in AcN from −0.8 to 0.8 V; threshold voltage *V*_th_ obtained from OECT transfer curves; maximum value of the product of hole mobility and volumetric capacitance, [μC*]_max_, obtained from OECT transfer curves; maximum volumetric capacitance 
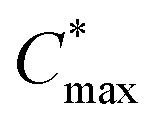
 determined through EIS; maximum hole mobility *μ*_max_ obtained by dividing [μC*] by C*. Values within brackets denote the corresponding gate potential for each value; the mean and min–max error of the values extracted from two devices are given

Polymer	*E*_ox,H_2_O_ (eV)	*E*_ox,AcN_ (eV)	*V*_th_ (V)	[μC*]_max_ (F cm^−1^ V^−1^ s^−1^)	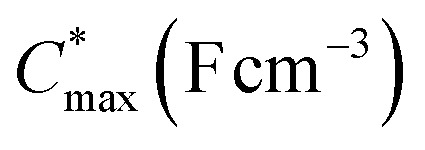	*μ*_max_ (cm^2^ V^−1^ s^−1^)
P1 (T)	4.35	4.69	−0.38 ± 0.02	168 ± 17 (−0.55 V)	>152 ± 17 (−0.6 V)	1.3 ± 0.2 (−0.5 V)
P2 (T2)	4.52	4.77	−0.35 ± 0.01	368 ± 9 (−0.55 V)	>215 ± 24 (−0.6 V)	1.8 ± 0.2 (−0.5 V)
P8 (BT)	4.48	4.77	−0.53 ± 0.01	50 ± 6 (−0.75 V)	148 ± 16 (−0.7 V)	0.3 ± 0.1 (−0.7 V)

Electrochemical impedance spectroscopy (EIS, see Fig. S53†) was used to determine the volumetric capacitance C* (see Fig. 5d). The C* values gradually increased with decreasing *V*_GS_ (=−offset potential *E vs.* Ag/AgCl) up to 152, 215 and 148 F cm^−3^ for P1 and P2 (at *V*_GS_ = −0.6 V) and P8 (at *V*_GS_ = −0.7 V), respectively (see Table 2). P1, P2, and P8 polymers exhibited comparable onset potential values for C* at *V*_GS_ = −0.1 to 0 V, attributed to their similar *E*_ox_ values (see Table 2). Also, it is notable that the electron-withdrawing character of BT did not significantly influence the onset and maximum value of the electrochemical capacitance. We divided the [μC*] values obtained from OECT characterization by C* from EIS to obtain *μ* values for P1, P2 and P8. The polymers P1 and P2 feature a high *μ*_max_ = 1.3 and 1.8 cm^2^ V^−1^ s^−1^ (see Table 2). Thin films of P1 and P2 comprise ordered domains with a face-on and edge-on texture, respectively (see Fig. 4b), with the latter being beneficial for in-plane charge transport, which may explain the somewhat higher *μ*_max_ value for P2. However, it can be expected that both the texture and degree of order are affected by electrochemical oxidation, as recently reported for other polythiophenes and thieno[3,2-*b*]thiophene–thiophene copolymers with oligoether side chains.^68,69^

Besides the *V*_th_, the onset and peak potentials for [μC*] and *μ* of P8 are also shifted to more negative values compared with P1 and P2. The higher magnitude of *V*_th_ of P8 based OECTs is tentatively assigned to a more localized highest-occupied molecular orbital (HOMO) on the g_3_TT unit in case of P8 (see inset Table S8†), which may increase the energy barrier for charge conduction along the polymer backbone, and thus a higher oxidation level is required for hopping of charges to occur in case of P8 compared to P1 and P2. Accordingly, P8 featured a lower *μ*_max_ = 0.3 cm^2^ V^−1^ s^−1^ despite having similar electrochemical properties as P1 and P2 (see Table 2). An alternative explanation of the lower *μ*_max_ is the lower order of P8 compared to P1 and P2 as per the UV-vis absorption spectra and GIWAXS diffractograms (see Fig. S46 and S47†). Comparison of the *μ*_max_ and 
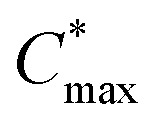
 values obtained for P1, P2 and P8 with those reported for other OMIECs, including other polymers with oligoether side chains as well as PEDOT:PSS, reveals that the here synthesized materials feature an electrochemical response that is comparable to state-of-the-art materials (see Fig. 1).

Under a pulsed gate potential, P2-based OECTs showed a promising degree of operational stability up to 200 cycles (see Fig. S54†). The on-current values at *V*_GS_ = −0.6 V decreased by only 6% from that of the initial cycle (*I*_on_ = 130 μA), while the off-current value at *V*_GS_ = +0.4 V gradually increased from 40 nA to 600 nA, which we assign to the gradual reduction of the polymer film by oxygen.^70^ Even under excessive electrochemical stress during the cyclic measurement (*i.e.*, *V*_GS_ = −0.6 and +0.4 V with *V*_DS_ = −0.6 V for on- and off-state), P2 shows a comparable operational stability as the state-of-the-art polymer p(g2T-TT),^41^ which was characterized with a smaller potential window (*V*_GS_ was cycled between −0.4 and +0.0 V with *V*_DS_ = −0.4 V).

## Conclusions

A variety of potential OMIEC materials was prepared by direct arylation polymerization (DAP) of a thieno[3,2-*b*]thiophene monomer with oligoether side chains (g_3_TT) with a variety of electron-rich, electron-deficient and neutral comonomers. In case of polymers that combined the monomer g_3_TT with the small comonomers thiophene, bithiophene or benzothiadiazole, the materials were active as p-type OMIECs in OECTs operating with an aqueous electrolyte. In particular the copolymer p(g_3_TT-T2) (P2) gave rise to a state-of-the-art OECT performance with a [μC*]_max_ value of 370 F cm^−1^ V^−1^ s^−1^, facilitated by a high 
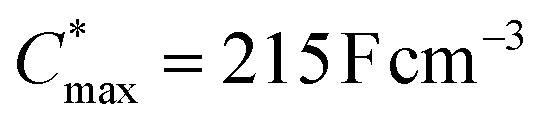
 and *μ*_max_ = 1.8 cm^2^ V^−1^ s^−1^. All synthesized polymers feature a lower synthetic complexity index (SCI) compared to many other polymers made through other polycondensation methods, which considerably enhances the scalability of conjugated polymers and is an important step toward the development of sustainable organic electronics.^12^

## Data availability

Data are available from the authors upon reasonable request.

## Author contributions

JK conceived the study and wrote the manuscript together with CM, and carried out the synthesis, single crystal X-ray diffraction, polymer characterization, UV-vis spectroscopy, cyclic voltammetry, and DFT calculations. YK fabricated and characterized OECT devices and carried out EIS. JA carried out GIWAXS measurements and processed data together with YK; JM supervised the GIWAXS analysis. RK and CM supervised the study.

## Conflicts of interest

The authors declare no conflict of interest.

## Supplementary Material

SC-015-D4SC01430H-s001
