# Supplementary material for: High-mobility organic mixed conductors with a low synthetic complexity index via direct arylation polymerization
Source: Chem Sci. 2024 Apr 19;15(20):7679–88. doi: 10.1039/d4sc01430h (PMC11110131; doi:10.1039/d4sc01430h)
Supplement: SC-015-D4SC01430H-s001 [file SC-015-D4SC01430H-s001.pdf]

Electronic Supplementary Material (ESI) for Chemical Science.
This journal is © The Royal Society of Chemistry 2024

Supplementary information

High-Mobility Organic Mixed Conductors with a Low Synthetic Complexity Index via Direct Arylation Polymerization

Joost Kimpel,[1]* Youngseok Kim,[1] Jesika Asatryan,[2] Jaime Martín,[2] Renee Kroon,[3] Christian Müller[1]*

[1]Department of Chemistry and Chemical Engineering, Chalmers University of Technology, 412 96 Göteborg, Sweden

[2]Universidade da Coruña, Campus Industrial de Ferrol, CITENI, Esteiro, 15403, Ferrol, Spain

[3]Wallenberg Initiative Materials Science for Sustainability, Department of Science and Technology, Linköping University, Norrköping, Sweden.

*e-mail: kimpel@chalmers.se; christian.muller@chalmers.se

Index

S1 Synthetic complexity index (SCI) **page 3**
S1.1 Definition
S1.2 Additional considerations
S1.3 Polymer structures
S1.4 Tabulated data of polymers in this work and literature
S1.5 Synthetic routes

S2 Experimental **page 23**
S2.1 Chemicals
S2.2 Analytical techniques and device details
S2.3 Monomer synthesis and characterization
S2.4 Polymer synthesis and characterization

S3 Polymer molecular weight determination by NMR **page 41**
S3.1 Derivation
S3.2 Uncertainties in molecular weight from NMR
S3.3 Molecular weight calculations for all polymers

S4 Material properties of polymers **page 46**
S4.1 Optical properties
S4.2 Grazing incidence wide angle X-ray scattering patterns
S4.3 Thermogravimetric analysis thermograms
S4.4 Electrical properties

S5 Device properties of polymers **page 53**
S5.1 OECT output characterization curves
S5.2 Electrochemical impedance spectroscopy characterization curves
S5.3 OECT cycling stability curves

S6 Computational results **page 56**

S7 References **page 57**

S1 Synthetic complexity index (SCI)

S1.1 Definition

A clear description and derivation of the synthetic complexity index (SCI) is found in the supplementary information of the perspective from Po *et al.* where they discuss the SCI in context of organic photovoltaics.[1] One alteration we include is the yield of the polymer synthesis in the yield term. Here, we summarize the main points of the calculation.

The normalized SCI is defined as equation S1:

$$SCI = a * \frac{NSS}{NSS_{max}} + b * \frac{\log RY}{\log RY_{max}} + c * \frac{NO}{NO_{max}} + d * \frac{NC}{NC_{max}} + e * \frac{NH}{NH_{max}} \quad \text{(S1)}$$

where NSS is the number of synthetic steps, RY is the reciprocal yield of the total yield, NO is the number of operations (specifically in our case quenching, precipitation, extraction, filtration/plug, recrystallization, Soxhlet, dialysis, and centrifugation), NC is the number of columns (specifically in our case flash column or preparative GPC), and NH is the number of hazard codes (a point is assigned if a chemical has any of the following hazard codes: H200-H205, H220, H222, H224, H240, H241, H250, H260, H261, H271, H290, H300, H304, H310, H314, H318, H330, H340, H341, H350, H351, H360, H361, H370, H372, H400, H410, and H411, taken from Po *et al.*). All numbers are compared against the maximum of each step in the list of compounds considered. The higher the number of a parameter, the more unfavorable the step. Multiple lists of SCI analyses can be compared if at least two compounds exist in the different SCI lists. This allows for the values to be renormalized.

The coefficients **a** – **e** are assigned semi-empirically according to overall cost and safety, leading to equation S2:

$$SCI = 35 * \frac{NSS}{NSS_{max}} + 25 * \frac{\log RY}{\log RY_{max}} + 15 * \frac{NO}{NO_{max}} + 15 * \frac{NC}{NC_{max}} + 10 * \frac{NH}{NH_{max}} \quad \text{(S2)}$$

S1.2 Additional considerations

In this section we elaborate on choices that have been made in this work which add onto/deviate from the SCI analysis done by Po *et al.*

Price of the starting materials and monomers

As described by Po *et al.*, the synthetic complexity index (SCI) is a description of cost of the total synthesis to a material. This makes the price of the starting material a plausible factor to analyze. Considering compounds made by polycondensation, from Fisher Scientific US or Millipore Sigma US (accessed 08-04-2024), a table to compare prices is made and shown on the next page.

The average price for a starting material or a monomer in a polymerization is \$182/g per reagent (as per $\frac{\sum(price \cdot no.\ of\ polymers\ employing\ reagent)}{\sum(no.\ of\ polymers\ employing\ reagent)}$). Accordingly, the average prices for the polymers in this work range from significantly below this average (\$110/g per reagent - \$127/g per reagent for **P1**, **P2**, **P4**, and **P8**) to be around the average (\$209/g per reagent - \$222/g per reagent for **P9** and **P3**), to going significantly above this average (\$241/g per reagent - \$371/g per reagent for **P6** and **P7**). The average of all polymers synthesized in this work is \$202/g per reagent. Ultimately, the values of the majority of compounds lies close to this average and accordingly we deem that the price of the starting material not necessary to consider.

Compound	Price ($USD/g)	Precursor to following polymer
4,7-dibromo-5,6-difluoro-2,1,3-benzothiadiazole	900	PgBT(F)2gTT, PgBT(F)2gTT, PgBT(F)2gTT-postmod
2,5-bis(trimethylstannyl)-thieno[3,2-*b*]thiophene	530	p(g_3T2-TT), p(g_4T2-TT), p(g_4T2-TT)-GPC, PgBTTT
1,3-bis(5-bromothiophen-2-yl)-5,7-bis(2-ethylhexyl)benzo[1,2-c:4,5-c']dithiophene-4,8-dione**	525	**P7**
7,7-dibromo-4,4,9,9-tetrahexyl-4,9-dihydro-s-indaceno[1,2-b:5,6-b']dithiophene	265	**P6**
2,9-dibromo-6,13-dioxatetracyclo[6.6.2.$0^{4,16}$.$0^{11,15}$]hexadeca-1,3,8,10,15-pentaene-5,7,12,14-tetrone	250	p(gNDI-g_3T2)
2,5-dibromothieno[3,2-*b*]thiophene	227	**P3**
3,6-dibromothieno[3,2-*b*]thiophene	217	**P1-P4**, **P6-P9**, PgBT(F)2gTT, PgBT(F)2gTT-postmod
5,7-bis(2-ethylhexyl)benzo[1,2-c:4,5-c']dithiophene-4,8-dione	200	**P9**
4,4'-didodecyl-2,2'-bithiophene	168	PBTTT
thieno[3,2-*b*]thiophene	112	PBTTT
2,5-bis(trimethylstannyl)thiophene	95	p(g_3T2-T), p(g_4T2-T)
3,6-bis(thiophen-2-yl)-1H,2H,4H,5H-pyrrolo[3,4-*c*]pyrrole-1,4-dione	90	PProDOT-DPP
3,3'-dibromo-2,2'-bithiophene	88	p(g_4T2-TT)
3,3-Bis(bromomethyl)-3,4-dihydro-2H-thieno[3,4-b][1,4]dioxepine**	71	PProDOT
5,7-dibromo-2H,3H-thieno[3,4-*b*][1,4]dioxine	37	PE2gT
5,5'-dibromo-2,2'-bithiophene	36	**P2**
4,7-dibromo-2,1,3-benzothiadiazole	27	**P8**
3-methoxythiophene	25	PgBTTT, p(g2T2- g4T2)
(thiophen-3-yl)methanol	24	P3MEEMT
2,7-dibromo-9,9-dioctyl-9H-fluorene	15	**P4**
3,4-dibromothiophene	12	PgBT(F)2gTT, PT2gT, PE2gT
2,5-dibromohydroxyquinone	12	inDTP-T, inDTP-T2
3-bromothiophene	4	inDTP-T, inDTP-T2, p(gNDI-g_3T2), p(gBDT-g_3T2), p(g_3T2-T), p(g_4T2-T), p(g_3T2-TT), p(g_4T2-TT), p(g_4T2-TT)-GPC, P3APPT
2,5-dibromothiophene	2	**P1**

Commercial availability of intermediates in synthetic protocols

In the analysis, syntheses are thoroughly followed as they have been reported. However, depending on a myriad of factors, authors might choose to start their synthesis at a certain molecule despite the commercial availability of an intermediate further in the synthetic route. Accordingly, the SCI analysis includes steps that might no longer be necessary owing to commercial availability. We account for this by including a ‘commercially available’ SCI

($SCI_{comm.avail.}$) in the analysis. These values would simulate the SCI if the commercially available compounds furthest in the synthetic protocol are used (excluding the final polymers themselves). One limitation imposed is that the commercial compound must be available at Fisher Scientific US or Millipore Sigma US (accessed 08-04-2024). For materials where compounds further in the synthesis are commercially available, an $SCI_{comm.avail.}$ is provided in red in Figures S2-S27.

Excess of reagents and use of solvents

For some reactions, authors might choose to use reagents as solvents. This could bias the SCI, by improving the yield whilst disregarding the potential additional cost. Using a similar approach as the first point (i.e. cost of the compounds at various vendors (accessed 08-04-2024)), the price of reagents used as solvents is determined and compared against the price of commonly used solvents. For instance, specific to this work, triethylene glycol monomethyl ether (between $70-100/L) is on the same order of other common solvents such as toluene (between $45-150/L), DMF (between $70-150/L), chlorobenzene (between $100-150/L), and pyridine (roughly $200-500/L). This comparison is the same for other cases where reagents are used as solvents.

End-capping of polymers

In our analysis, the literature procedures are followed as reported and end-capping is considered as a quenching operation in the polymerization step. This is attributed to end-cappers stopping the growing chains in the polymerization. The identity of end-cappers have little influence on performance of materials in e.g. OPVs,[2] but molecular weight of the

polymer does affect the performance significantly. Since end-cappers help obtain the right molecular weight, their addition is considered as a quenching step operation.

S1.3 Literature polymer structures

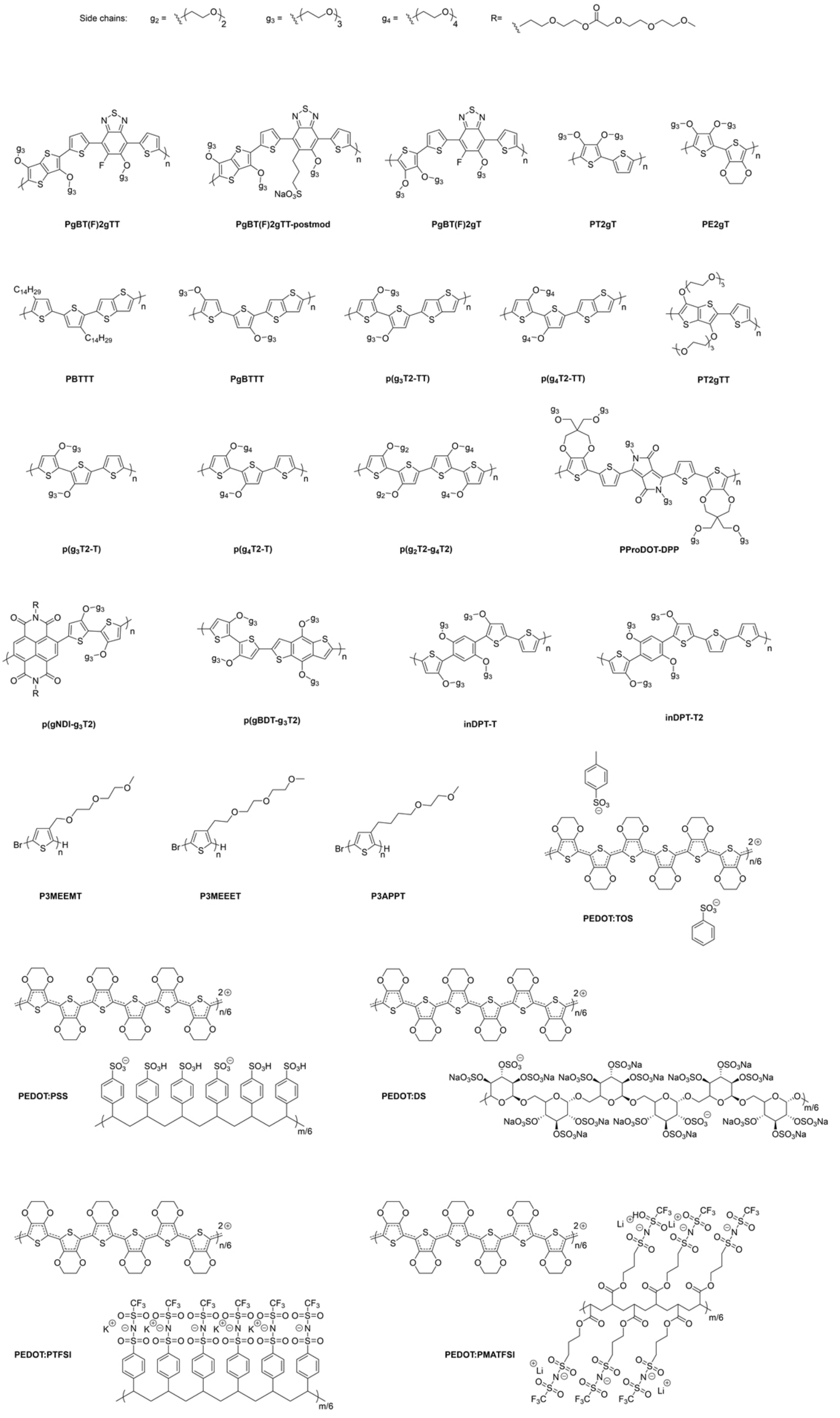

Figure S1. Polymers from literature considered for SCI calculations.

S1.4 Tabulated data of polymers in this work and literature

Table S1. Synthetic complexity index (SCI) determination of synthesized polymers and polymers from literature.

					Operations									Hazards								
Polymer	SCI	**Number of Synthetic Steps**	Yield	**Reciprocal Yield (100/Yield)**	Quenching	Precipitation	Extraction	Plug	Recrystallization	Distillation	Soxhlet / Dialysis / Centrifuge	**Sum of Operations**	**Number of Columns**	Carcinogenic / mutagenic (H340, H341, H350, H351)	Toxic for reproduction (H360, H361)	Highly toxic (H300, H304, H310, H330, H370, H372)	Environmentally toxic (H400, H410, H411)	Highly reactive with water (H260, H261)	Highly flammable (H220, H222, H224, H250)	Highly corrosive (H290, H314, H318)	Explosive (H200, H201, H202, H203, H204, H205, H240, H241, H271)	**Sum of Hazard Codes**
**P1** (T)	30	2	23	4	1	2	2	0	1	1	4	11	1	2	5	6	5	1	1	5	0	25
**P2** (T2)	32	2	13	8	1	2	2	0	1	0	4	10	1	2	5	6	5	1	1	5	0	25
**P3** (TT)	31	2	18	6	1	2	2	0	1	0	4	10	1	2	5	6	5	1	1	5	0	25
**P4** (F)	31	2	15	7	1	2	2	0	2	0	3	10	1	1	4	5	5	1	1	5	0	22
**P6** (IDT)	31	2	13	8	1	2	2	0	1	0	3	9	1	1	4	5	5	1	1	5	0	22
**P7** (BBDD)	30	2	24	4	1	2	2	0	1	0	4	10	1	2	5	6	5	1	1	5	0	25
**P8** (BT)	29	2	20	5	1	2	2	0	1	0	4	10	1	2	5	6	5	1	1	5	0	25
**P9** (BDD)	29	2	32	3	1	2	2	0	1	0	4	10	1	2	5	6	5	1	1	5	0	25
PgBT(F)2gTT	47	4	5	19	0	2	2	0	0	0	5	9	2	4	9	5	5	2	0	5	0	30
PgBT(F)2gTT-postmod	57	5	4	24	0	4	2	0	0	0	7	13	2	5	11	9	5	3	0	0	6	39
PgBT(F)2gT	42	4	17	6	0	2	2	0	0	0	5	9	2	4	9	5	5	2	0	5	0	30
PT2gT	26	2	31	3	0	2	1	0	0	0	5	8	1	1	6	7	4	1	1	2	0	22
PE2gT	26	2	33	3	0	2	1	0	0	0	5	8	1	1	6	7	4	1	1	2	0	22
PT2gTT	35	2	4	24	0	2	1	0	0	0	5	8	1	1	6	7	4	1	1	2	0	22
PProDOT-DPP	67	6	3	32	1	2	3	0	0	0	4	10	5	6	16	9	7	0	0	6	0	44
inDTP-T	71	6	3	30	2	1	3	2	0	0	4	12	5	8	14	16	8	2	3	8	0	59
inDTP-T2	82	8	7	15	1	1	5	2	0	0	4	13	6	10	17	19	13	3	3	10	0	75
PBTTT	36	3	24	4	1	2	2	0	1	0	3	9	2	4	2	8	6	1	1	5	0	27
PgBTTT	50	4	6	16	1	1	2	0	0	0	5	9	3	4	8	12	8	0	2	7	0	41
p(gNDI-g_3T2)	74	5	1	200	1	2	2	0	1	0	5	11	5	5	10	20	11	3	3	8	0	60
p(gBDT-g_3T2)	93	7	1	200	1	2	5	0	4	0	4	16	7	6	14	22	13	4	5	7	0	71
p(g_4T2-T)	51	4	9	11	1	3	4	0	0	0	4	12	3	5	6	7	6	2	4	8	0	38
p(g_3T2-T)	53	4	16	6	1	2	1	1	0	0	5	11	5	4	9	13	9	2	4	6	0	47
p(g_3T2-TT)	53	4	12	8	1	3	2	2	2	0	5	15	3	4	9	11	7	2	4	5	0	42
p(g_4T2-TT)	36	3	43	2	1	2	1	1	0	0	6	11	2	4	5	10	6	1	0	6	0	32
p(g_4T2-TT)-GPC	44	3	11	9	1	2	1	1	0	0	6	11	3	5	6	11	6	1	0	6	0	35
p(g_2T2-g_4T2)	83	7	1	83	1	2	4	0	1	0	5	13	6	6	11	19	9	3	4	11	0	63
P3MEEMT	36	4	58	2	2	2	0	1	0	0	3	8	2	6	4	6	4	1	1	7	0	29
P3MEET	31	3	43	2	2	1	2	1	0	0	3	9	1	6	4	6	4	1	1	7	0	29
P3APPT	45	4	11	9	1	2	1	2	0	0	1	7	3	9	5	8	5	2	0	8	0	37
PEDOT:PSS-SA	11	1	71	1	1	0	0	0	0	0	4	5	0	0	0	0	0	0	0	3	0	3
PEDOT:PSS-EG	11	1	71	1	1	0	0	0	0	0	4	5	0	0	0	0	0	0	0	2	0	2
PEDOT:DS	7	1	87	1	0	0	0	0	0	0	2	2	0	0	0	0	0	0	0	0	0	0
PEDOT:PSTFSI	28	4	43	2	1	1	1	1	1	0	0	5	0	1	2	2	1	1	0	9	0	16
PEDOT:PMATFSI	34	5	41	2	0	0	4	0	1	0	0	5	0	2	6	1	1	1	0	8	0	19
PEDOT:TOS	6	1	90	1	1	0	0	0	0	0	0	1	0	0	0	0	0	0	0	4	0	4

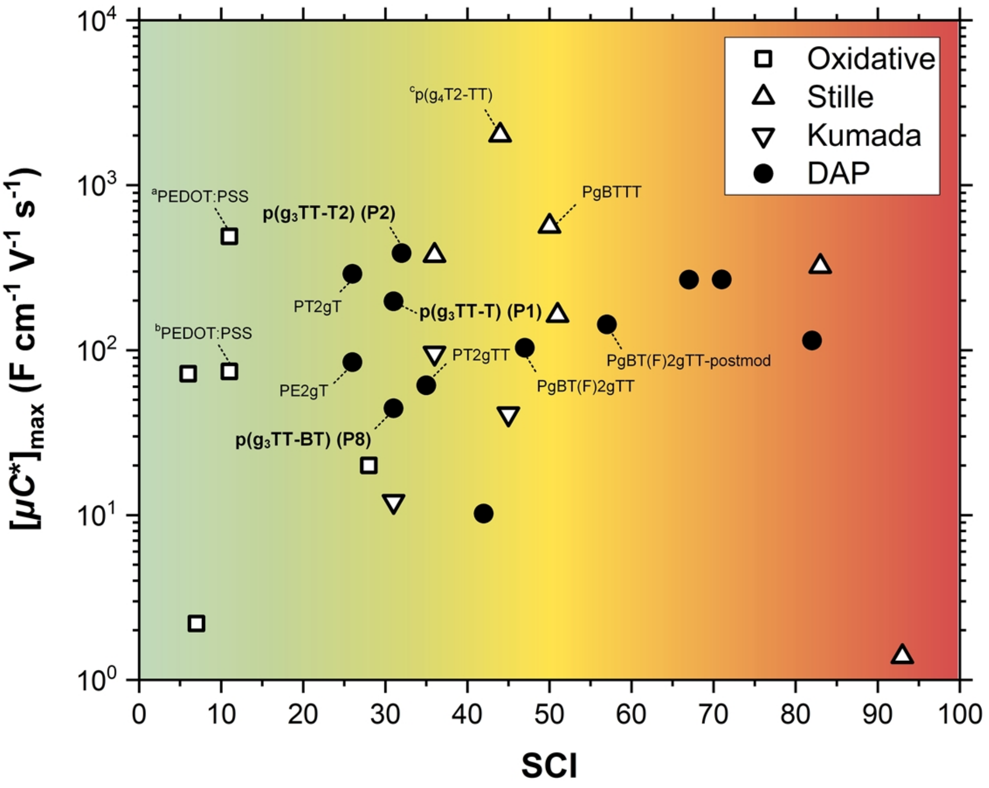

Figure S2. Ashby plot of SCI *vs* [μC*]$_{max}$ and selected polymers synthesized in this work (**bold**) by oxidative polymerization (squares), Stille coupling (upward triangles), Kumada coupling (downward triangles) or DAP (circles), with the synthetic complexity index (SCI) of each synthesis indicated by a red-green scale. [a]Poly(3,4-ethylenedioxythiophene):poly(styrene sulfonate) (PEDOT:PSS) post-treated with sulfuric acid, [b]PEDOT:PSS post-treated with ethylene glycol, [c]p(g$_4$T2-TT) (see Figure S1 for chemical structure) fractionated by size-exclusion chromatography (highest molecular weight fraction, catalyst removed).

Table S2. Mean SCI and standard error in SCI for polymers in this work. [a] Standard error determined by $SE = \sigma/\sqrt{n}$, where σ is standard deviation and n is sample size ($n = 12$). [b] Standard error determined by $SE = \sigma/\sqrt{n}$, where σ is standard deviation and n is sample size ($n = 6$). [c] Error determined by the mean and min-max error of the values ($n = 2$). [d] No repeats performed, i.e. no error determined. Error assumed as percentage error from **P1** reactions.

<table>
<tr><th>Material</th><th>Mean yield g_3TT synthesis (%)</th><th>Mean yield polymer synthesis (%)</th><th>Average total yield (%)</th><th>Average SCI</th></tr>
<tr><td>P1</td><td rowspan="8">47.1 ± 2.9[a]</td><td>47.8 ± 4.1[b]</td><td>22.5 ± 2.4</td><td>31.3 ± 1.4</td></tr>
<tr><td>P2</td><td>27.7 ± 2.3[b]</td><td>13.0 ± 1.3</td><td>33.9 ± 1.5</td></tr>
<tr><td>P3</td><td>37.5 ± 3.5[c]</td><td>17.7 ± 2.0</td><td>32.4 ± 1.6</td></tr>
<tr><td>P4</td><td>31.3 ± 2.7[d]</td><td>14.7 ± 1.6</td><td>31.9 ± 1.4</td></tr>
<tr><td>P6</td><td>27.2 ± 2.3[d]</td><td>12.8 ± 1.4</td><td>32.5 ± 1.5</td></tr>
<tr><td>P7</td><td>50.4 ± 4.3[d]</td><td>23.7 ± 2.5</td><td>31.0 ± 1.4</td></tr>
<tr><td>P8</td><td>42.5 ± 11.5[c]</td><td>20.0 ± 5.6</td><td>31.8 ± 3.8</td></tr>
<tr><td>P9</td><td>67.2 ± 5.8[d]</td><td>31.6 ± 3.4</td><td>29.7 ± 1.3</td></tr>
</table>

S1.5 Synthetic routes

Reagents are shown before or above arrows. Work-up chemicals are shown below arrows. If chemicals are repeated in the work-up chemicals, it means they are used for different work-up steps. Yields of steps are shown underneath the product of each step. Total yield of the material is given in **bold** at the end of the synthesis. Operations are abbreviated as follows: Q = quenching, Prec = precipitation, E = extraction, Plug = silica plug or cumbersome filtration, R = recrystallization, Sox = Soxhlet, D = dialysis, Dist = distillation, Cent = centrifugation, C = flash column chromatography or preparative gel permeation chromatography. The synthetic complexity index (SCI) is provided at the end of the synthesis. In case a 'commercially available' SCI ($SCI_{comm.avail.}$) is determined (SCI from commercially available compounds further in the synthetic route), a red box annotates the new starting point of the synthesis, new total yield in red font, and $SCI_{comm.avail.}$ is provided at the end of the synthesis in red font.

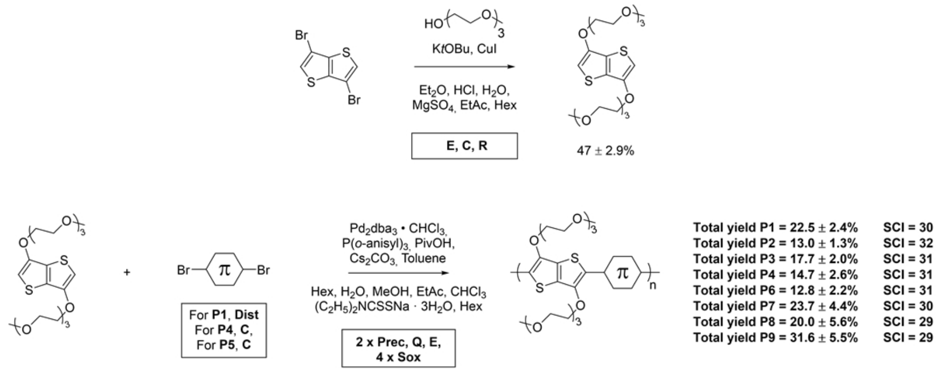

Figure S3. Synthesis including SCI information of **P1**, **P2**, **P3**, **P4**, **P6**, **P7**, **P8**, and **P9** as reported in this work.

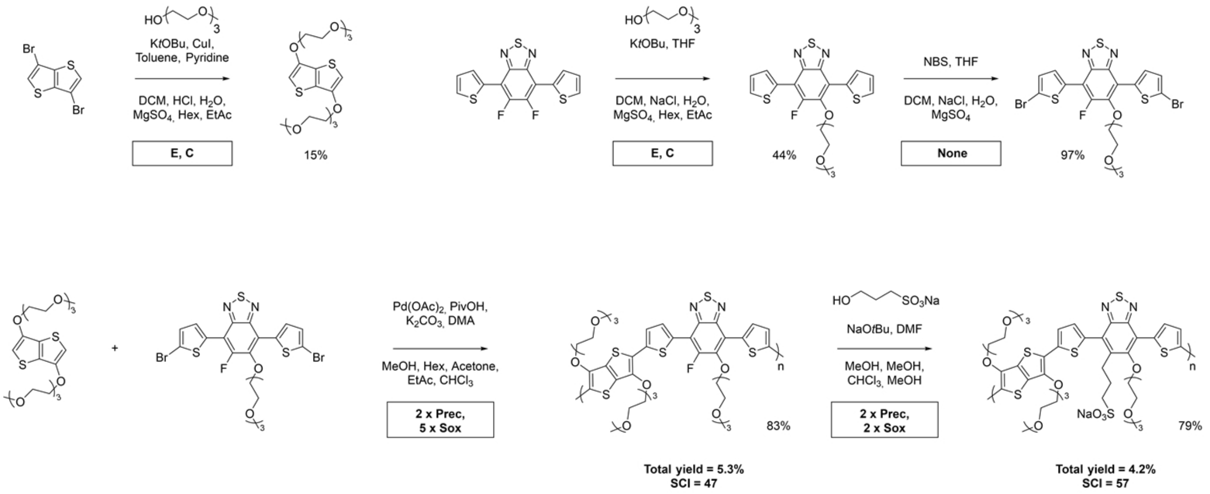

Figure S4. Synthesis including SCI information of PgBT(F)2gTT and post polymerization modified PgBT(F)2gTT.[3, 4]

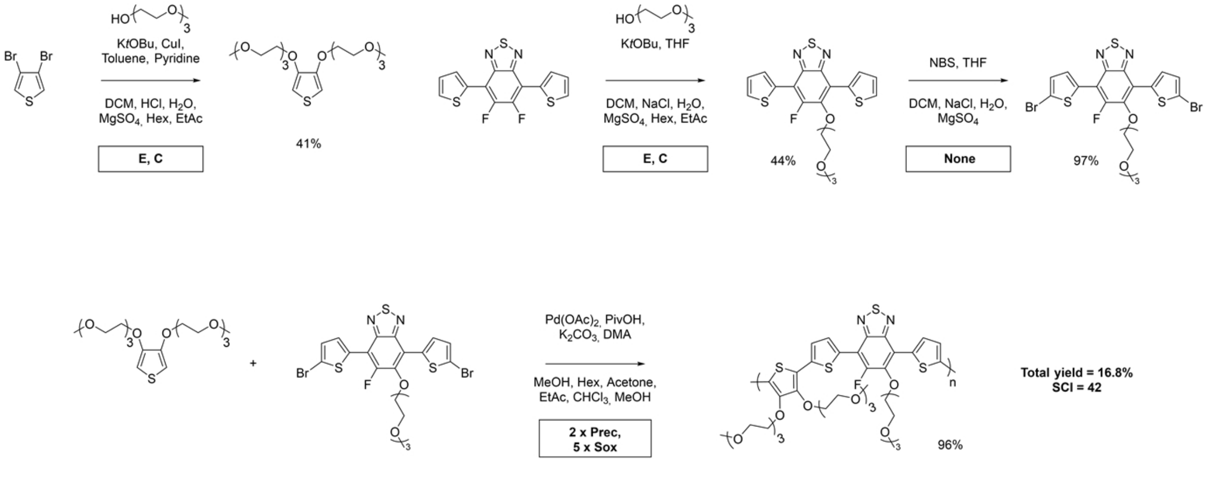

Figure S5. Synthesis including SCI information of PgBT(F)2gT.[4]

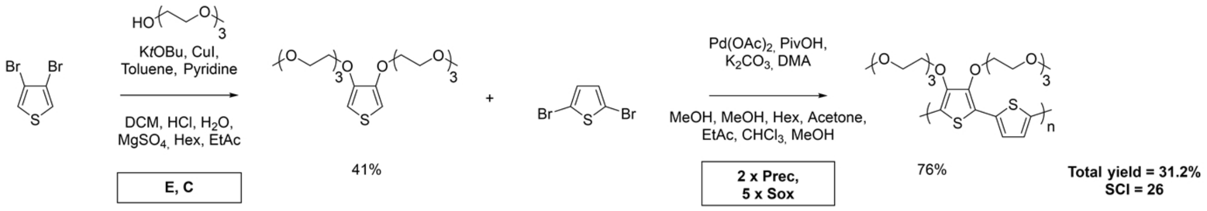

Figure S6. Synthesis including SCI information of PT2gT.[5]

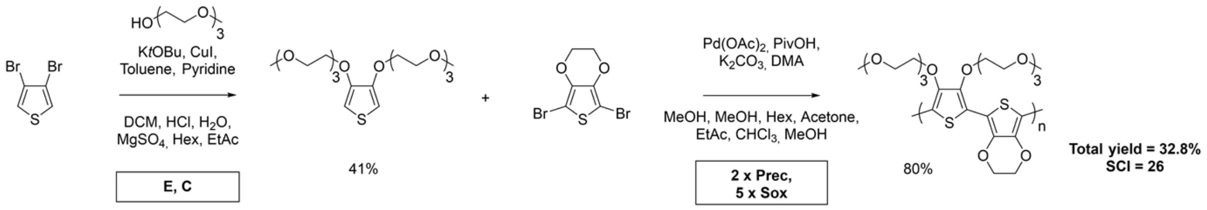

Figure S7. Synthesis including SCI information of PE2gT.[5]

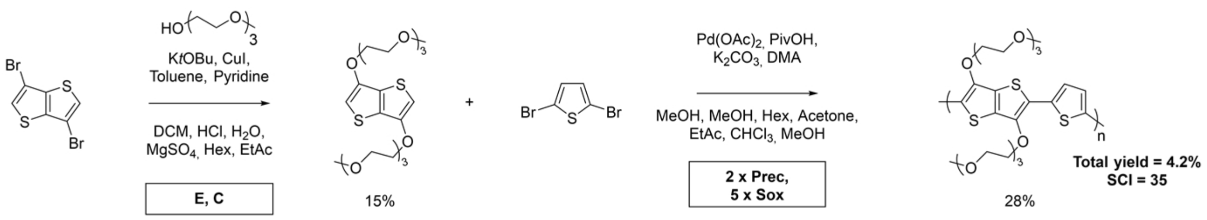

Figure S8. Synthesis including SCI information of PT2gTT.[5]

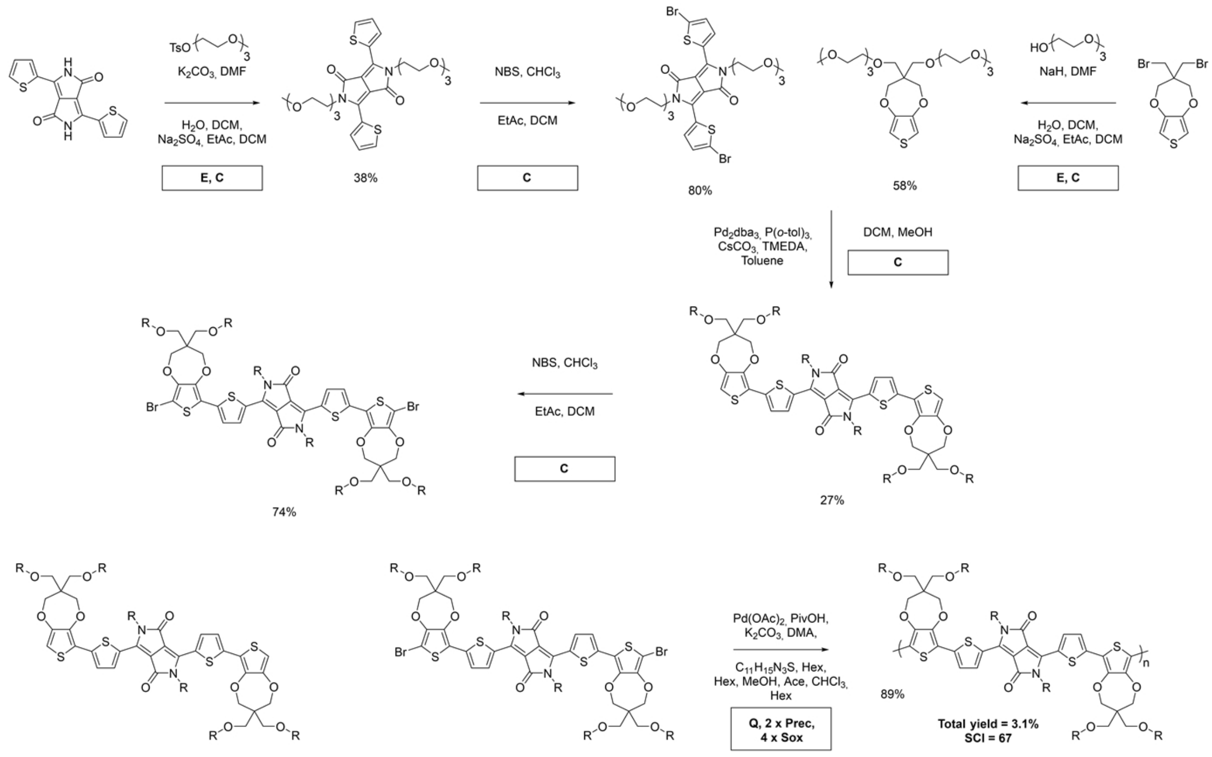

Figure S9. Synthesis including SCI information of PProDOT-DPP.[6]

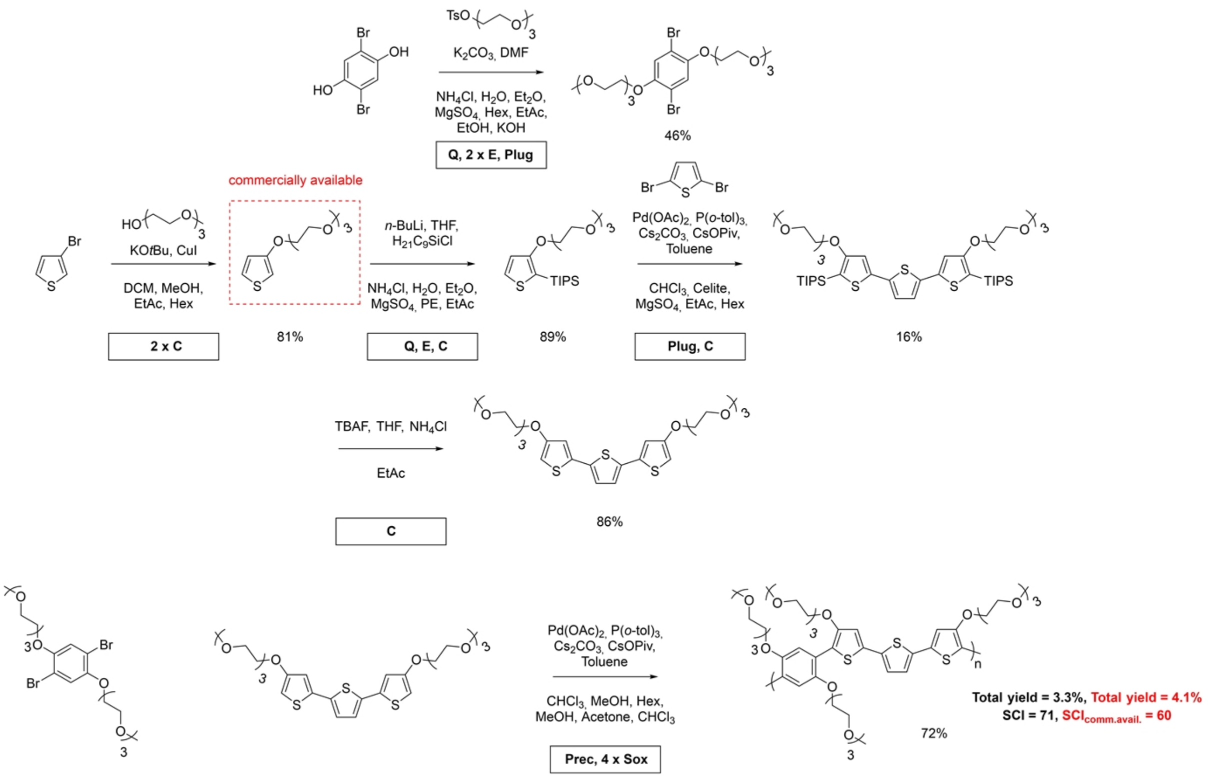

Figure S10. Synthesis including SCI information of inDTP-T.[7]

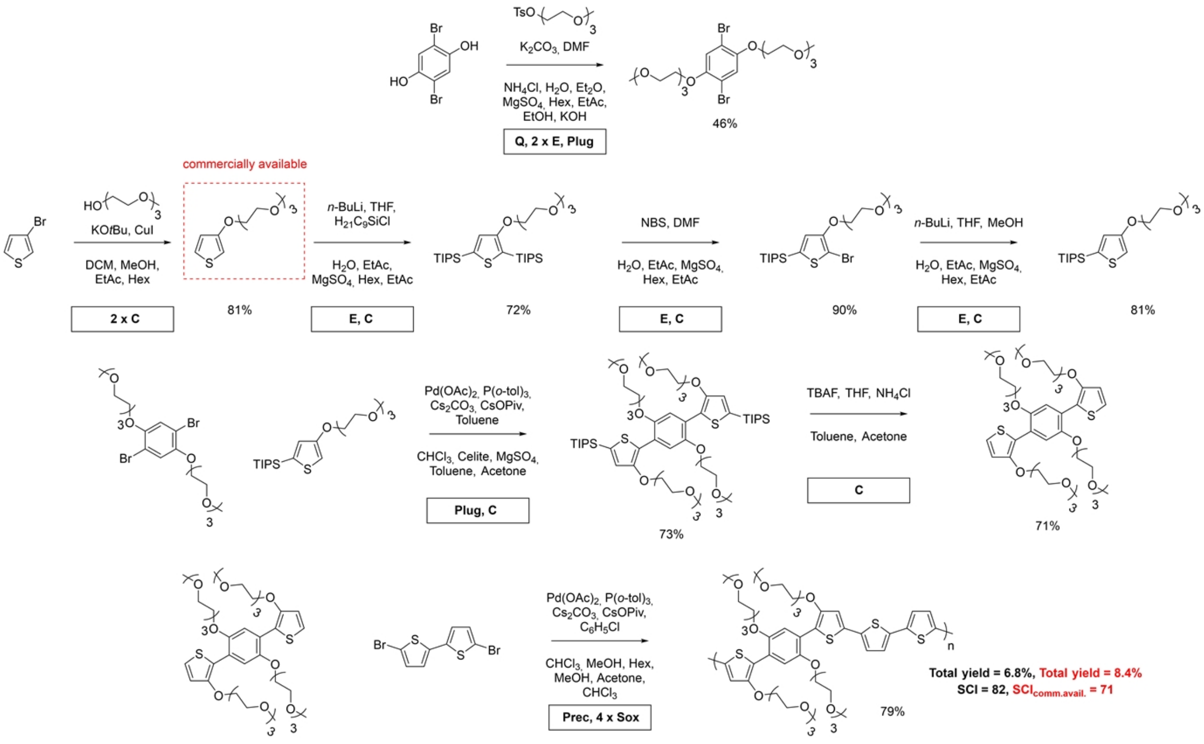

Figure S11. Synthesis including SCI information of inDTP-T2.[7]

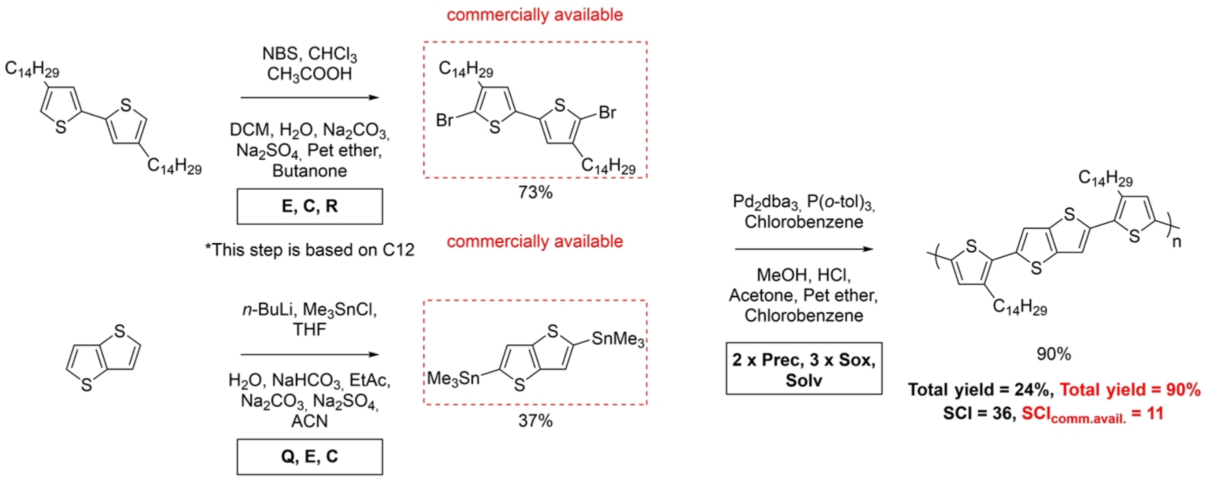

Figure S12. Synthesis including SCI information of PBTTT-C14.[8]

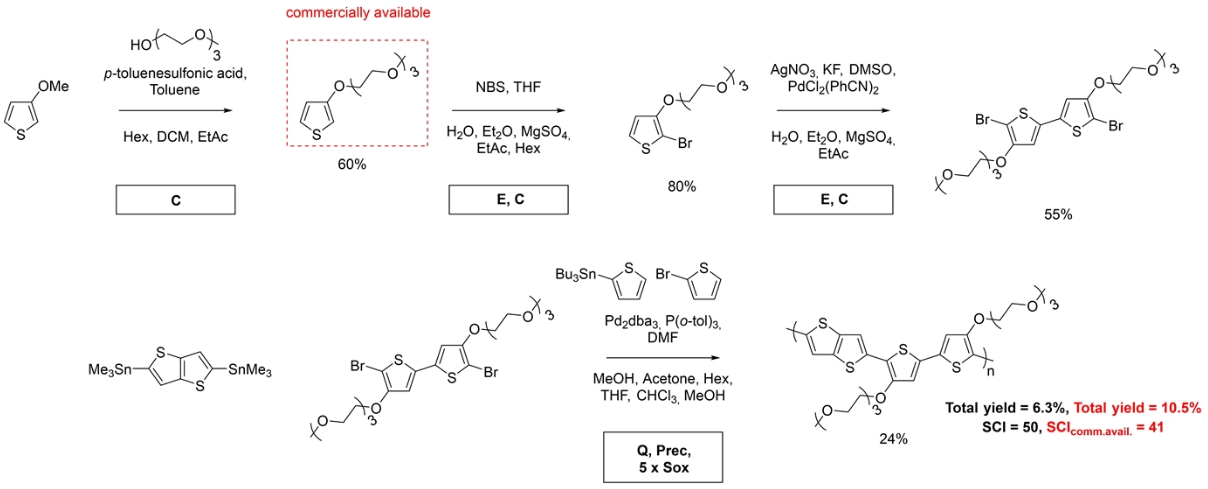

Figure S13. Synthesis including SCI information of PgBTTT.[9]

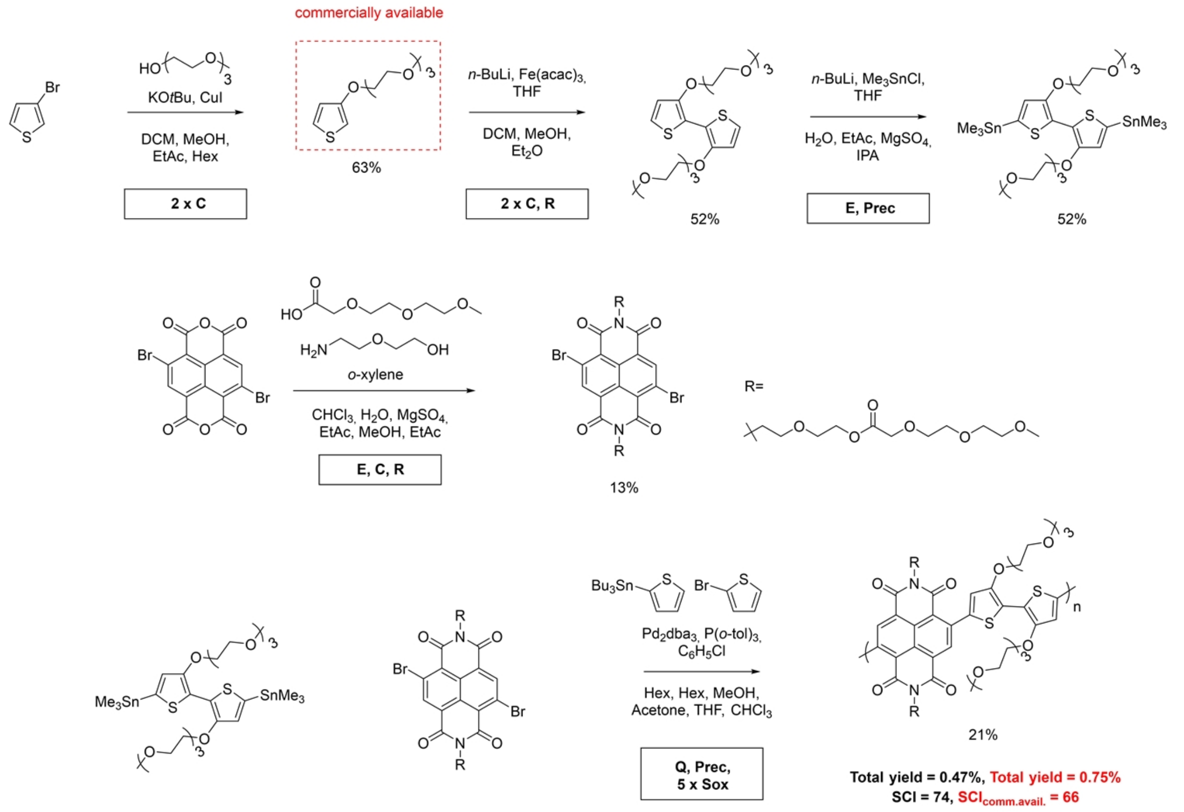

Figure S14. Synthesis including SCI information of p(gNDI-g_3T2).[10, 11]

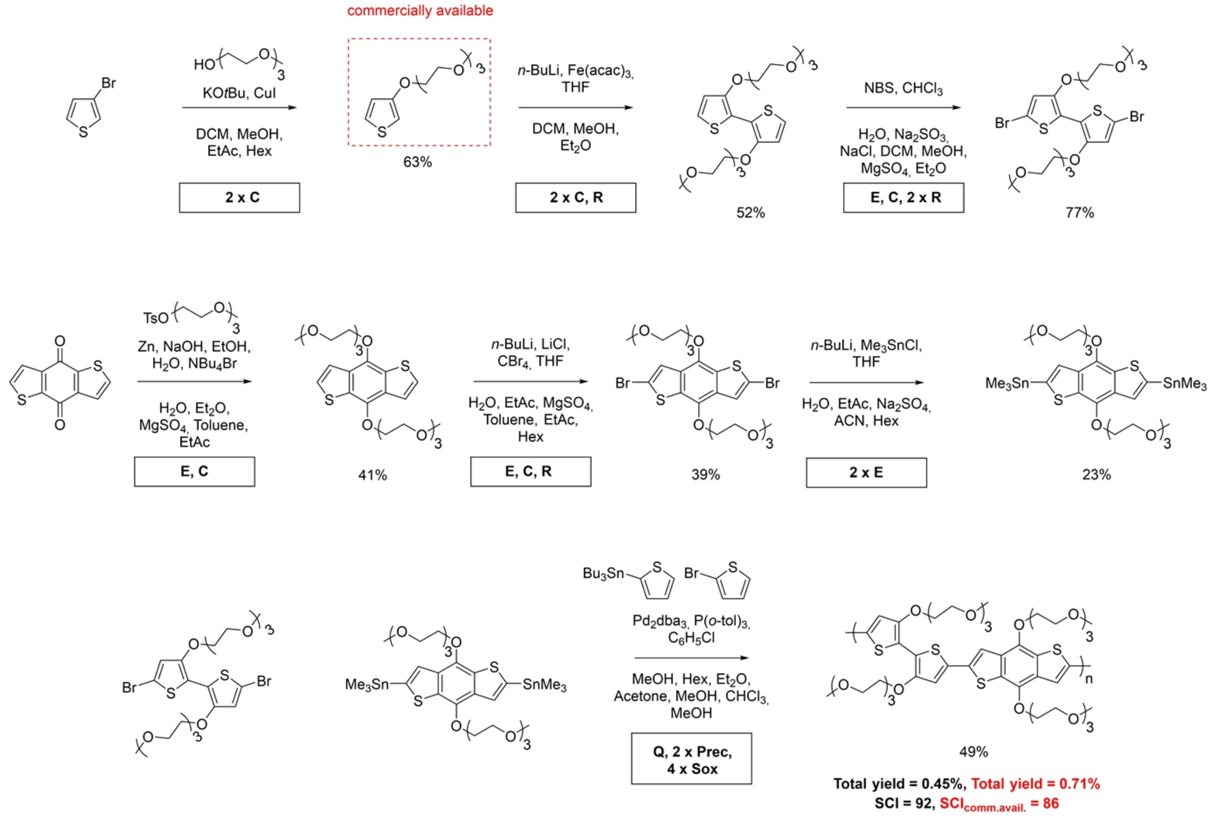

Figure S15. Synthesis including SCI information of p(gBDT-g_3T2).[12, 13]

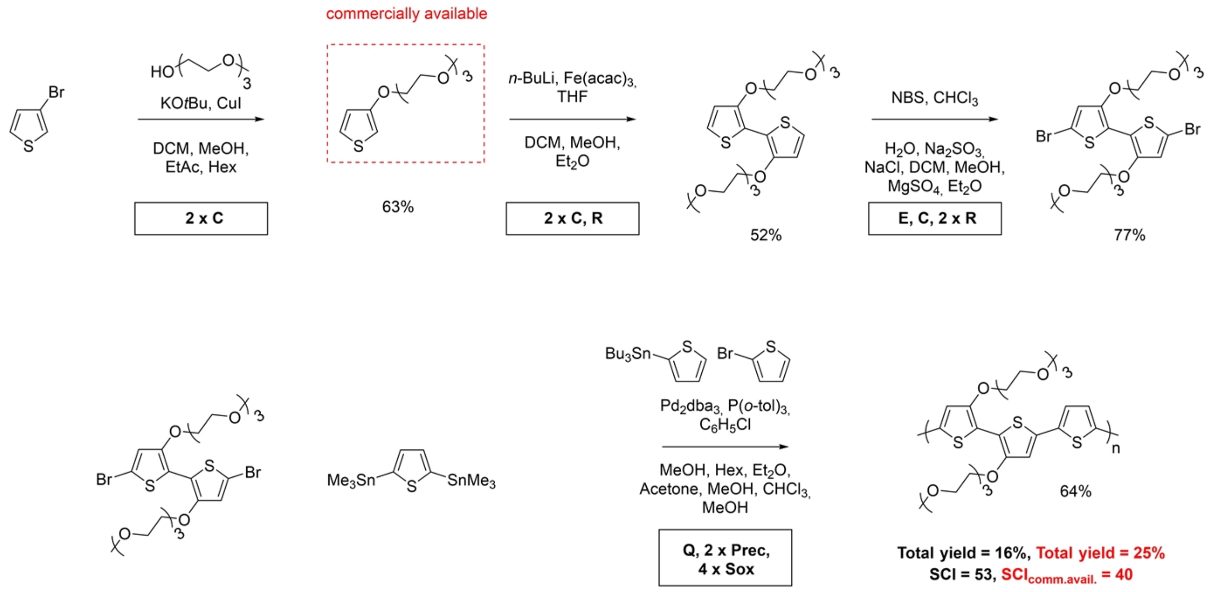

Figure S16. Synthesis including SCI information of p(g_3T2-T).[13]

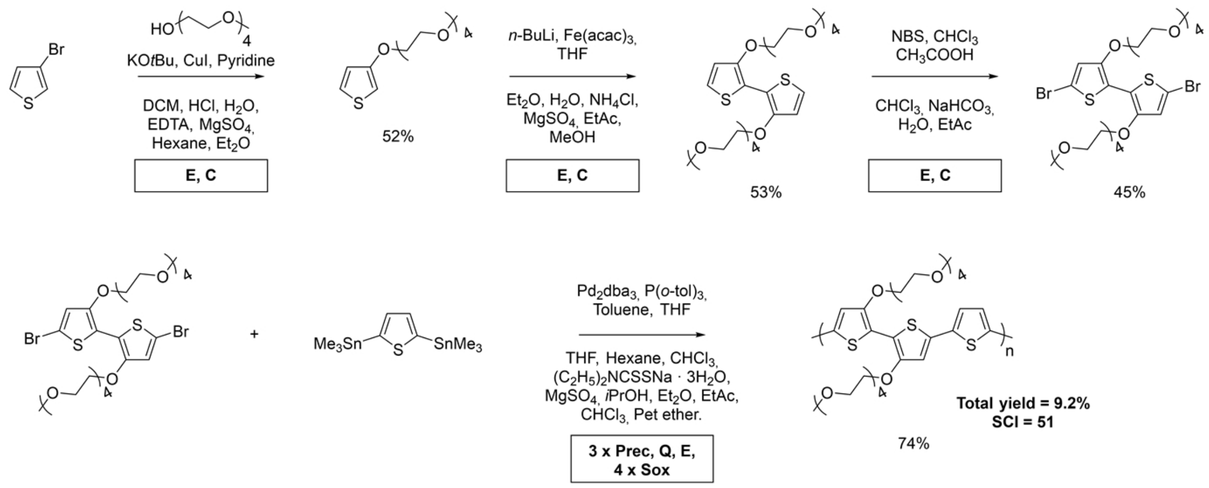

Figure S17. Synthesis including SCI information of p(g_4T2-T).[14]

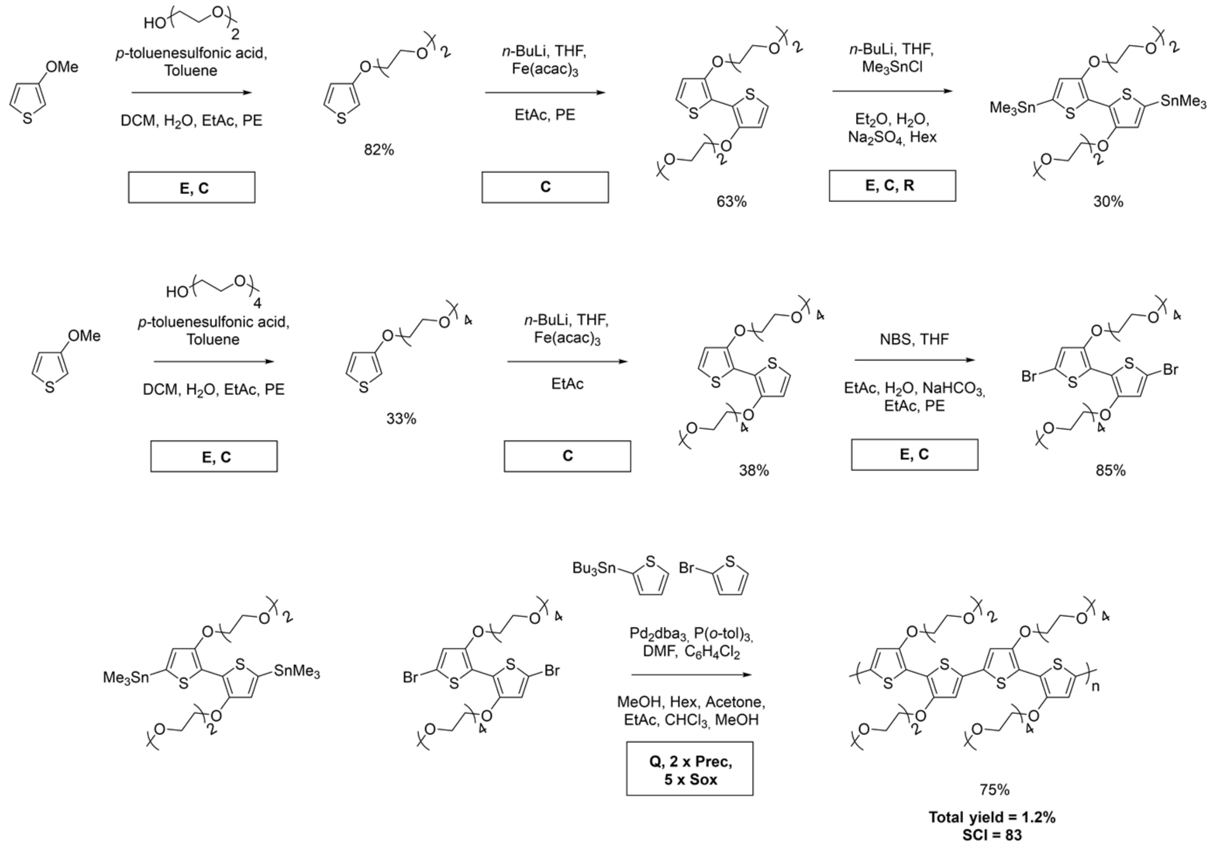

Figure S18. Synthesis including SCI information of p(g_2T2- g_4T2).[15]

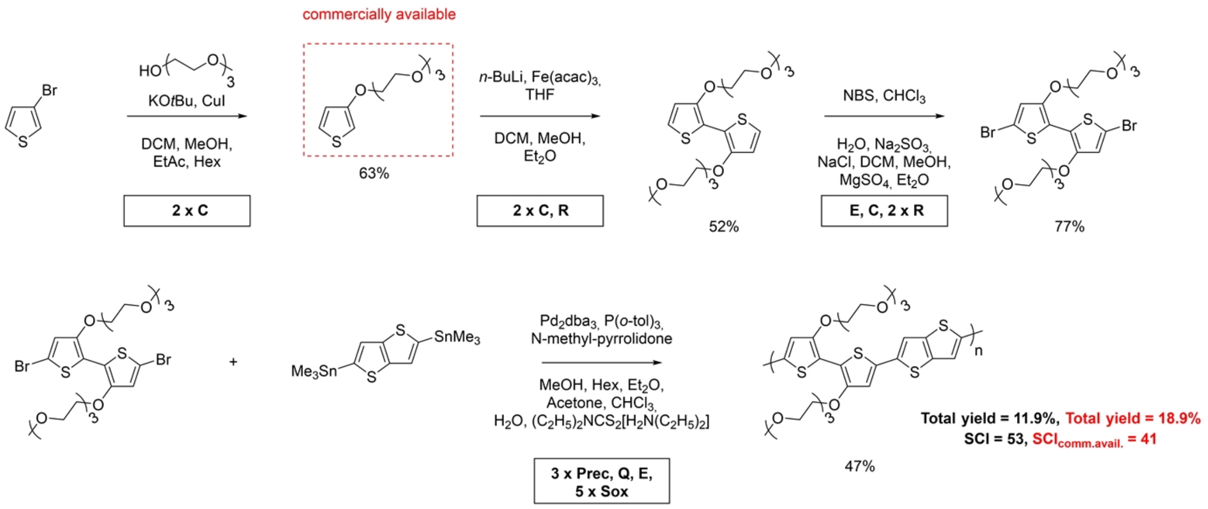

Figure S19. Synthesis including SCI information of p(g_3T2-TT).[16]

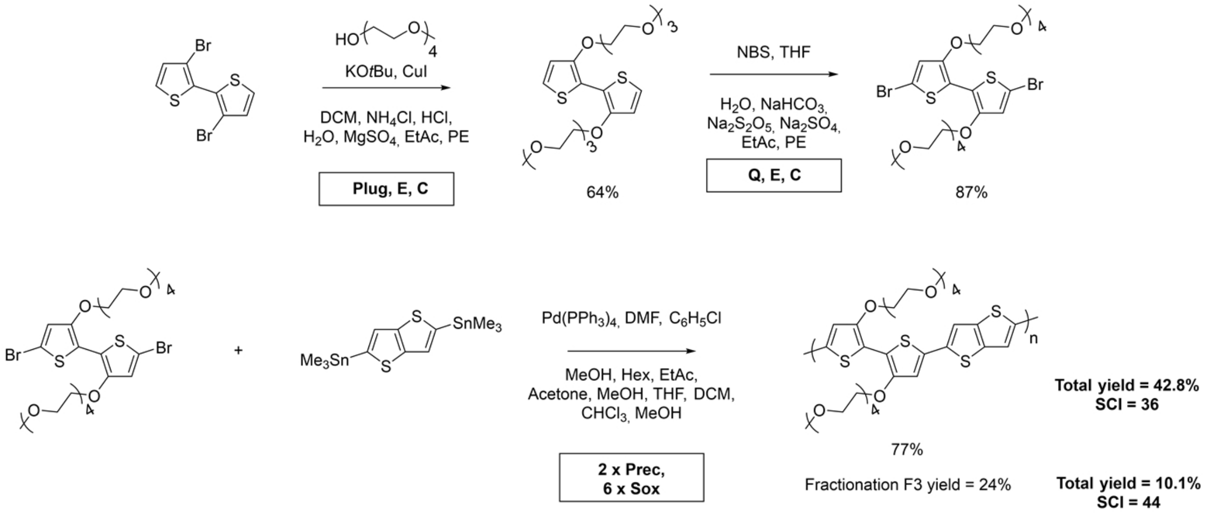

Figure S20. Synthesis including SCI information of p(g_4T2-TT) and preparative GPC purified p(g_4T2-TT).[17]

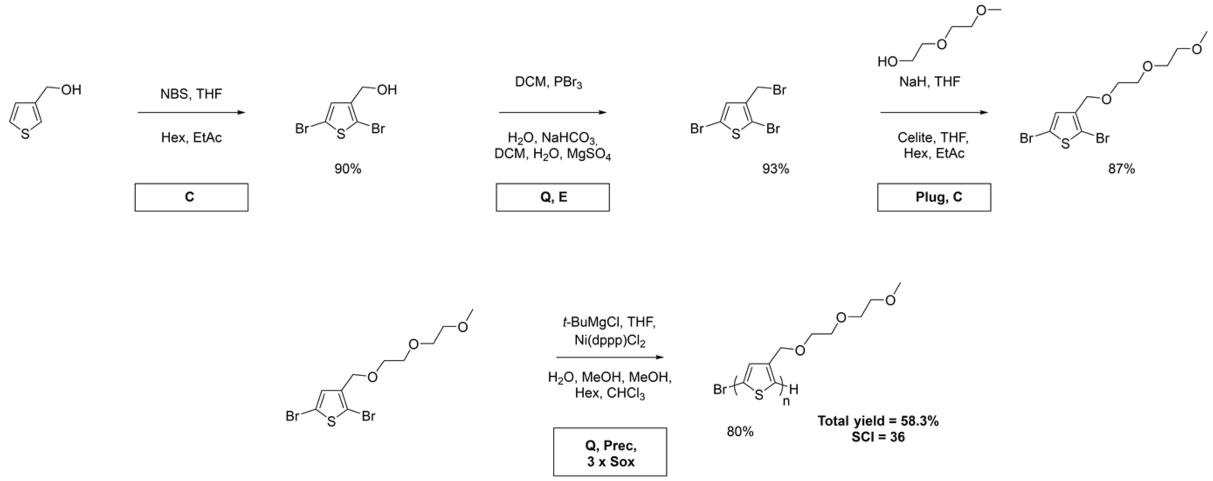

Figure S21. Synthesis including SCI information of P3MEEMT.[18]

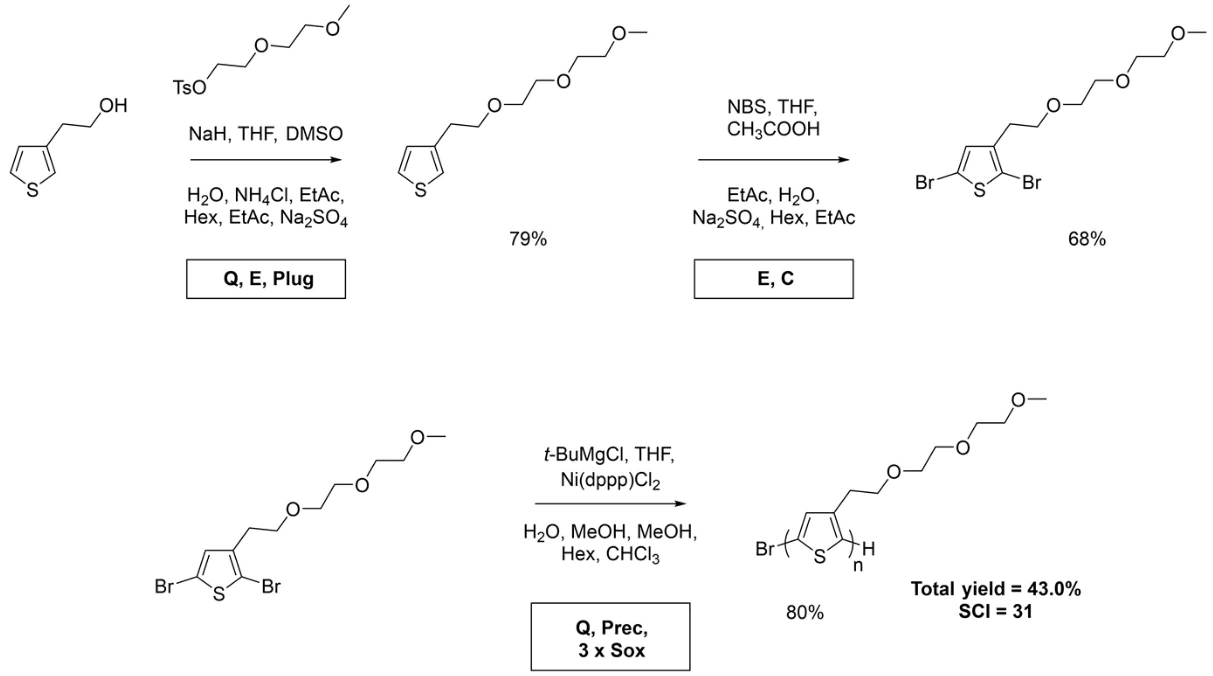

Figure S22. Synthesis including SCI information of P3MEEET.[18]

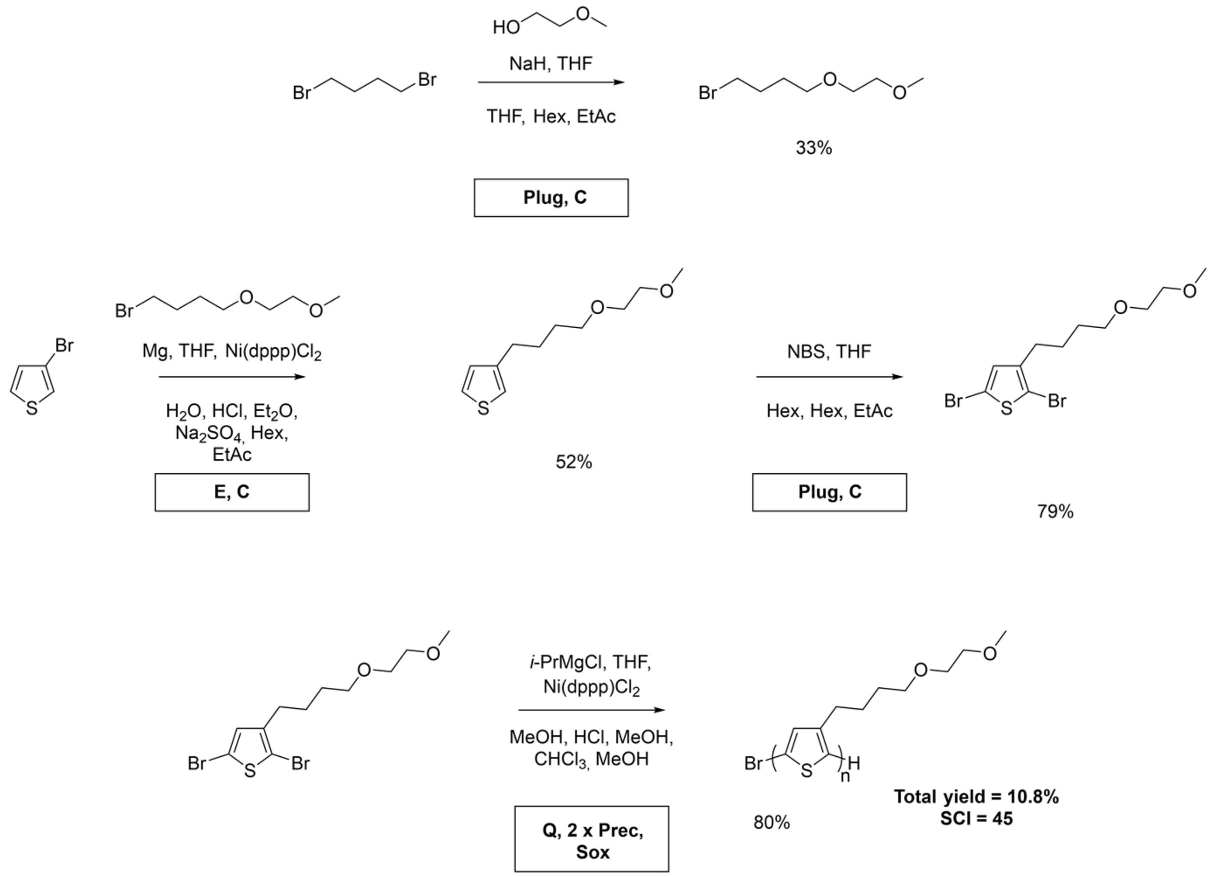

Figure S23. Synthesis including SCI information of P3APPT.[19]

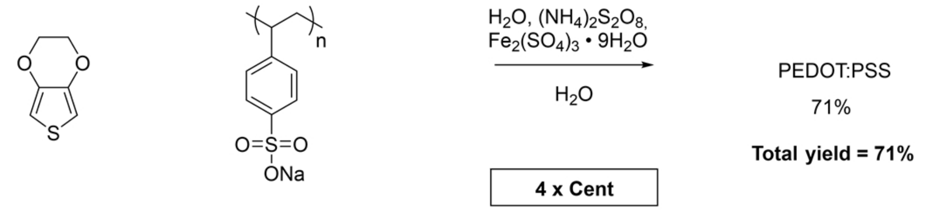

The following are not considered as synthetic steps, and give 100% yield:

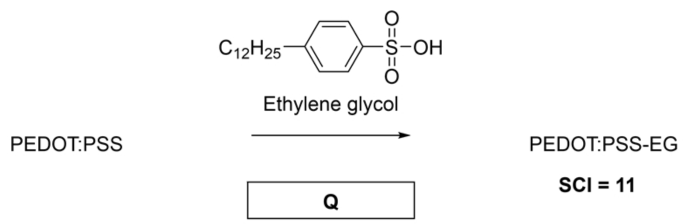

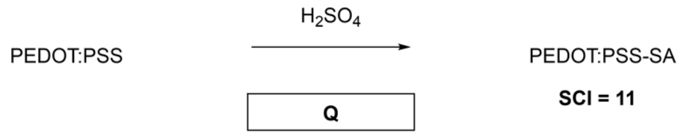

Figure S24. Synthesis including SCI information of PEDOT:PSS and post-treated PEDOT:PSS.[20-23]

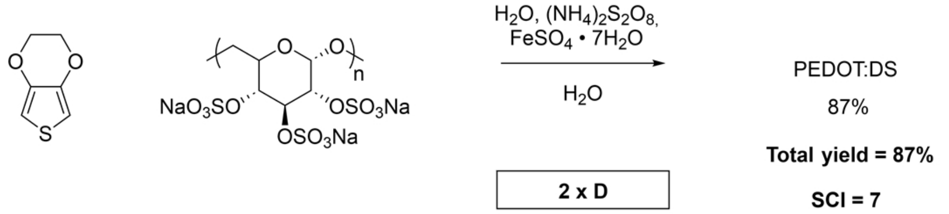

Figure S25. Synthesis including SCI information of PEDOT:DS.[20, 21]

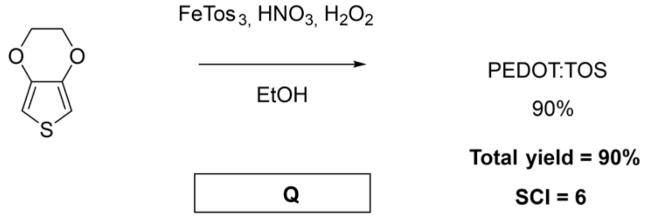

Figure S26. Synthesis including SCI information of PEDOT:TOS.[21, 24]

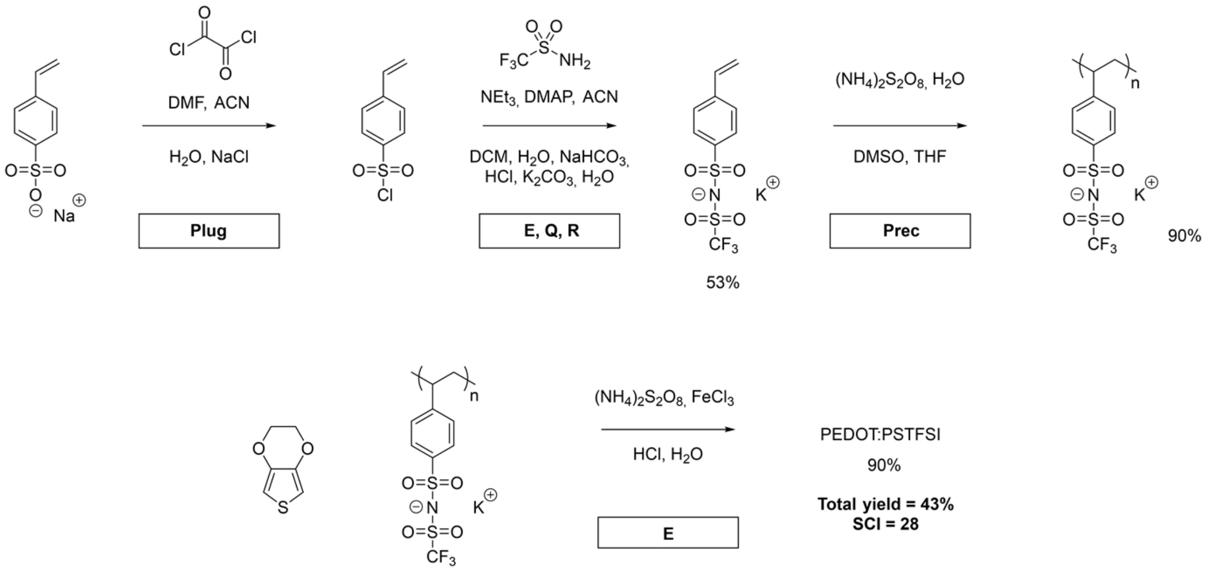

Figure S27. Synthesis including SCI information of PEDOT:PSTFSI.[21, 25-27]

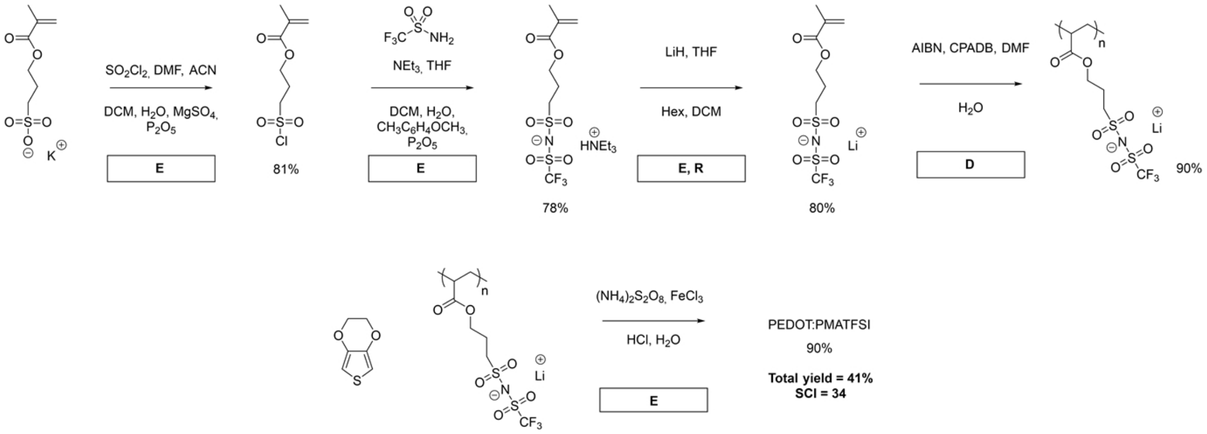

Figure S28. Synthesis including SCI information of PEDOT:PMATFSI.[21, 28, 29]

S2 Experimental

S2.1 Chemicals

Triethylene glycol monomethyl ether, *t*BuOK, CuI, amine ligands, hydroxide bases, pivalic acid, cesium carbonate, tris(dibenzylideneacetone)dipalladium(0)-chloroform adduct and sodium diethyldithiocarbamate trihydrate were received from Sigma Aldrich and VWR and used without further purification. Analytical grade hexane, ethyl acetate, diethyl ether, methanol, and chloroform were obtained from Fisher Scientific and used as received. Analytical grade toluene and tetrahydrofuran (THF) were purchased from Fisher Scientific, dried, charged into a solvent purification system, and directly taken as required. 3,6-dibromothieno[3,2-*b*]thiophene (BLDpharm, 98%) was obtained through Chemtronica. Other comonomers were obtained from various vendors: 2,5-dibromothiophene (TCI, >95%), 5,5'-dibromo-2,2'-bithiophene (TCI, >98%), 2,5-dibromothieno[3,2-*b*]thiophene (TCI, >98%) and 3,6-bis(5-bromothiophen-2-yl)-2,5-bis(2-ethylhexyl)pyrrolo[3,4-c]pyrrole-1,4(2*H*,5*H*)-dione (BLDpharm, 95%) were obtained from VWR; 9,9-dioctyl-2,7-dibromofluorene (Sigma Aldrich, 96%) and 4,9-dibromo-2,7-bis(2-octyldodecyl)benzo[*lmn*][3,8]phenanthroline-1,3,6,8(2*H*,7*H*)-tetrone (Sigma Aldrich, ≥99%) were obtained from Sigma Aldrich; 4,7-dibromo-2,1,3-benzothiadiazole (GR-Chem, 98%), 7,7-dibromo-4,4,9,9-tetrahexyl-4,9-dihydro-*s*-indaceno[1,2-*b*:5,6-*b*']dithiophene (GR-Chem, 98%), 1,3-bis(5-bromothiophen-2-yl)-5,7-bis(2-ethylhexyl)benzo[1,2-*c*:4,5-*c*']dithiophene-4,8-dione (GR-Chem, 98%) and 5,7-bis(2-ethylhexyl)benzo[1,2-*c*:4,5-*c*']dithiophene-4,8-dione (GR-Chem, 98%) were obtained from JiangSu GR-Chem Pharma Technology Ltd. In case the purity was below 98%, solid comonomers were recrystallized from pentane or methanol at -20 °C and oven-dried before use. 2,5-Dibromothiophene was purified through distillation before use.

S2.2 Analytical techniques and device details

Nuclear Magnetic Resonance. Room temperature and high temperature spectra were recorded on a Bruker Avance NEO 600 spectrometer (^{1}H: 600.13 MHz, ^{13}C: 150.90 MHz). The ^{1}H and ^{13}C NMR spectra were referenced to the residual solvent peak ($CDCl_3$: $\delta(^{1}H)$ = 7.26 ppm, $\delta(^{13}C)$ = 77.16 ppm; $C_2D_2Cl_4$: $\delta(^{1}H)$ = 5.98 ppm).

Single crystal X-ray crystallography. The monomer crystal structures were obtained by mounting suitable crystals on a nylon loop on an XtaLAB Rigaku Synergy R, HyPix diffractometer using CuKα radiation (λ = 1.54184 Å). The crystals were kept at a steady temperature T = 116.0(2) K during data collection. Their structures were solved with the ShelXT21 structure solution program using the Intrinsic Phasing solution method and by using Olex2 as the graphical interface.[30, 31]

Photographs. An EOS-RP, Canon was used to capture pictures of the thin films. Substrates and films were held against a white background. Digital Photo Professional software was used to match (and correct against) the white balance and ImageJ software was used to obtain the RGB values of the colors.

Size exclusion chromatography. Chromatograms were recorded using an Agilent 1260 Infinity GPC running at an oven temperature of 70 °C, employing two columns and a precolumn containing Polargel M 300 × 7.5 mm with mixed pores and a pore size of 8 µm. Polymer samples were dissolved in dimethylformamide (DMF) at a concentration of about 1 g L^{-1}. The eluent used was DMF (DMF, Sigma, HPLC-grade, ≥99.9%) with 0.1 wt% LiBr (Sigma, Reagentplus®, ≥99.9%). Relative calibration was carried out with poly(methyl methacrylate) (PMMA) standards.

Matrix-assisted laser desorption/ionization – time of flight (MALDI-ToF). The MALDI-TOF MS spectra were taken using BRUKER autoflex maX MALDI-TOF instrument in reflectron

negative mode. The laser of this instrument is a smartbeam-II with a wavelength of 355 nm. The software flexControl 3.4 and flexAnalysis 3.4 are used for measuring and evaluating the spectra, respectively. For all samples 20 g L^{-1} DCTB in chloroform was used as matrix. Samples were prepared on a standard sample plate (Bruker "MTP 384 target plate polish steel BC") using double layer method. 1 μL sample solution in chloroform 1 g L^{-1} is deposited on the sample plate and dried in air. Then, 1 μL matrix solution was dropped on top of the sample droplet and dried in air.

Film thickness. The film thickness was determined using a KLA Alphastep Tencor D-100 profilometer or NTEGRA NT-DMT instrument.

Grazing incidence wide angle X-ray scattering (GIWAXS). Polymer solutions in chloroform with a concentration of 5 - 10 g L^{-1} were prepared and spin-coated onto cleaned and plasma treated silicon wafers. GIWAXS patterns were recorded at the beamline NCD – SWEET of the Alba synchrotron light source facility using an X-ray wavelength of 1 Å and a sample detector distance of 201.17 cm. To characterize the GIWAXS diffractograms, each peak was fitted using a Gaussian curve after eliminating the linear background. The crystalline structure distance d was determined from the peak location of each fitted Gaussian, while the grain size L was estimated using the Scherrer equation $L = 2\pi K/(\Delta q_r)$, where K and Δq_r represent a shape factor (= 0.9) and the full-width-half-maximum (FWHM) of the fitted Gaussians, respectively. For peaks which overlapped with a π-π stacking and halo pattern, the q value at the maximum intensity describing the center of the Gaussian was set as the primary peak (e.g., π-π stacking for **P1**, **P7**, and **P8**, and halo peak for **P2**, **P3**, **P4**, **P6**, and **P9**), then an additional Gaussian curve was added to account for the near shoulder peak. The orientation of the polymer was estimated from in-plane and out-of-plane GIWAXS diffractograms.

Thermal analysis. Differential scanning calorimetry (DSC) thermograms were recorded with a Mettler Toledo DSC2 equipped with a gas controller GC 200 system. For three consecutive cycles, samples were heated/cooled between 25 °C to 300 °C under nitrogen atmosphere (flow rate = 60 mL min^{-1}) using a heating/cooling rate of 10 °C min^{-1}. Thermogravimetric analysis (TGA) thermograms were recorded with a Mettler Toledo TGA/DSC 3+. Samples were heated from 25 °C to 450 °C (flow rate = 60 mL min^{-1}) using a heating/cooling rate of 10 °C min^{-1}.

UV-Vis-NIR absorption. UV-Vis-NIR spectra of thin films were recorded with a PerkinElmer Lambda 1050 spectrophotometer. Spectra were normalized by the film thickness obtained with AFM/profilometry.

Cyclic voltammetry (CV) and electrochemical impedance spectroscopy (EIS). Polymer films were spin-coated from chloroform solutions (5 - 10 g L^{-1}) onto an ITO-coated glass substrate (Ossila, 20 Ω cm^{-2}). Then, the thin films were patterned with a size of ~0.5 cm^2 using a swab soaked in chloroform. The exposed ITO layer was passivated with epoxy resin, separating the glass substrate in one part with the active film and another exposed ITO part. The side without the thin film was connected with a crocodile clip and the side with the polymer film was submerged in the solution during analysis. Electrochemical characterization was performed with a CH instrument CHI 650D using a three-electrode configuration. For the characterization in an aqueous electrolyte (0.1 M NaCl/H_2O), an Ag/AgCl reference electrode was used, while in case of a non-aqueous electrolyte (0.1 M, NBu_4PF_6/acetonitrile), a Ag/Ag^+ reference electrode was used. A Pt wire served as the counter electrode in both cases. Before and during the measurement, the electrolyte was degassed by nitrogen bubbling. CV curves were recorded with a scan rate of 50 mV s^{-1} in the range of -0.4 V to +0.6 V and -0.8 V to +0.8 V for the aqueous and non-aqueous electrolyte, respectively. After the measurement, the capacitive background current, which was generated by the ITO layer, was subtracted by

using the voltammogram curve obtained from a bare ITO substrate under identical measurement conditions. The oxidation onset potential Φ_{ox} was extracted from the tangent fitted to the oxidation peak of the second cycle. In case of the aqueous electrolyte and Ag/AgCl reference electrode, the reference potential E^0 of the electrode was taken as $E^0 = 4.4$ eV. In the case of the non-aqueous electrolyte and Ag/Ag^+ electrode, the potential was calibrated using the redox peak of the ferrocene/ferrocenium (Fc/Fc^+) redox couple, which was measured prior to the characterization and its reference potential was taken as $E^0 = 5.1$ eV. Accordingly, oxidation potentials were calculated by $E_{ox} = \Phi_{ox} + E^0$.

The EIS spectra were recorded in a frequency range from 10^{-1} to 10^5 Hz, and the offset potential was varied from -0.4 V to +0.6 V vs. Ag/AgCl with a 20 mV peak-to-peak sinusoidal signal. The electrochemical capacitance of the active layer was extracted from EIS data using the EIS Spectrum Analyser software and an equivalent circuit model $R_e[R_sC_s[R_c[R_aC_a]]]$,where R_e, R_s, C_s, R_c, R_a and C_a are the resistance of the electrolyte, electrochemical resistance/capacitance of the ITO substrate, contact resistance between the substrate and the active layer, and electrochemical resistance/capacitance of the active layer, respectively. The obtained values for C_a were normalized by the volume = $d \times A$ of the active layer, where d and A are the film thickness and area, respectively.

Organic electronic chemical transistor (OECT) device fabrication and characterization. The source and drain metal electrodes were defined via a lift-off process using a Karl Suss MA6 contact aligner and a Kurt J Lesker PVD e-beam evaporator on cleaned Marienfeld soda lime glass slides, resulting in channels with a width $w = 200$ µm and length $L = 20$ µm. Then, active layers were spin-coated from chloroform solutions (7 g L^{-1}) at 1500 rpm yielding films with a thickness of $d = 50$ to 120 nm. The active layers were partially removed near the contact pads with a swab soaked in chloroform. Then, a glass reservoir was attached to the OECT channel region with an elastomer sealing (Sylgard-184, Corning), and 15 mL of an

aqueous electrolyte (100 mM NaCl) was placed in the reservoir. Device characterization was conducted with two MATLAB-controlled Keithley 2400 source-measure units. The gate potential was applied through the electrolyte by using a three-electrode configuration with an Ag/AgCl reference electrode and Pt wire counter electrode.

Density-functional theory (DFT) calculations. Calculations were performed using the Gaussian 16 and Gauss View 6 package on the Vera cluster of the Chalmers Center for Computational Science and Engineering (C3SE). Geometry optimization to reach stable conformations of trimers was performed using the ωB97XD functional with 6-31+G(d,p) level of theory. Side chains were truncated to methyl-groups, in case of alkyl side chains, or methoxy-groups, in case of glycol side chains. Optimized structures were validated by vibrational analysis.

S2.3 Monomer synthesis and characterization

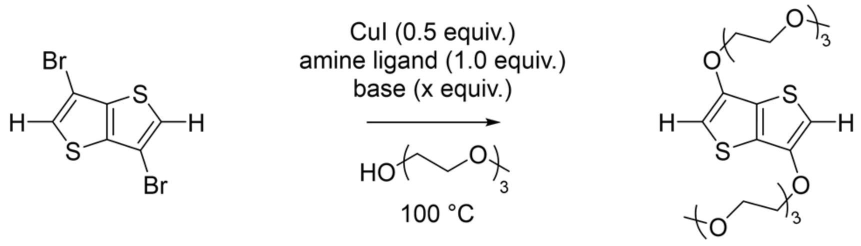

Figure S29. Monomer synthesis optimization.

Triethylene glycol monomethyl ether (53.5 mL, 336 mmol, 10 equiv.), *t*BuOK (15.1 g, 134 mmol, 4.0 equiv.), and CuI (3.20 g, 16.8 mmol, 0.50 equiv.) were added to a dried 250 mL two-neck round bottom flask mounted with a condenser and stirred for 1 h at room temperature under inert atmosphere. Under vigorous stirring, 3,6-dibromothieno[3,2-*b*]thiophene (10.0 g, 33.6 mmol, 1.0 equiv.) was added after which the flask was evacuated and brought under inert atmosphere and left to react for 24 h at 100 °C. The reaction was monitored throughout with thin liquid chromatography using ethyl acetate as the eluent (R_f = 0.45 - 0.6). After 24 h, the reaction mixture was cooled to room temperature and diluted with 250 mL diethyl ether. The mixture was filtered into a separatory funnel to remove residual solids and washed with 3 × 200 mL hydrochloric acid ($[H^+]$ = 1 M). The combined aqueous layer was extracted with 250 mL diethyl ether. The combined organic layer was dried over anhydrous $MgSO_4$ and concentrated *in vacuo*. The brown crude oil was passed through a silica column with a gradient of ethyl acetate : hexane = 1 : 1 going to pure ethyl acetate as the eluent. The product fraction was collected and concentrated. The product was separated into several 250 mL single-necked round bottom flasks and recrystallized from diethyl ether at -20 °C to yield yellow crystals (7.32 g, 15.8 mmol, 47%). ^1H NMR ($CDCl_3$, 600 MHz): δ 6.28 (s, 2H), 4.22 (t, J = 4.8 Hz, 4H), 3.87 (t, J = 4.7 Hz, 4H), 3.75-3.72 (m, 4H), 3.70-3.63 (m, 8H), 3.56-3.53 (m, 4H), 3.37 (s, 6H); ^{13}C NMR ($CDCl_3$, 100 MHz): δ 149.9, 128.7, 98.6, 72.1, 71.0, 70.8, 70.7, 70.1, 69.7, 59.2.

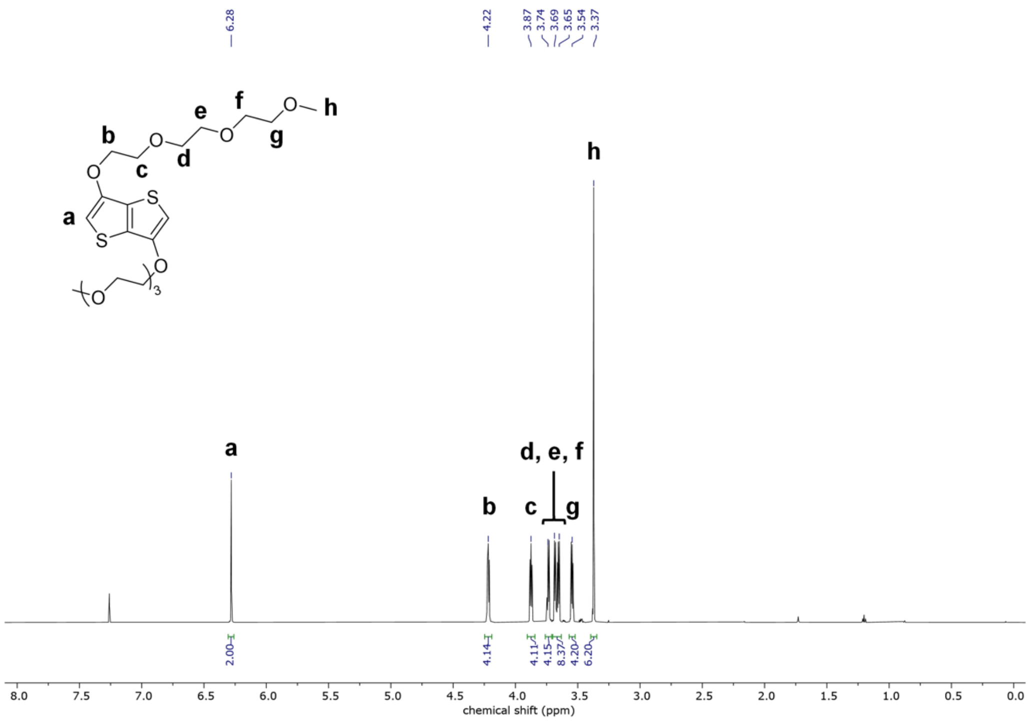

Figure S30. ^{1}H NMR spectrum ($CDCl_3$, 600 MHz, 298K) of 3,6-bis(triethylene glycol monomethyl ether)thieno[3,2-*b*]thiophene.

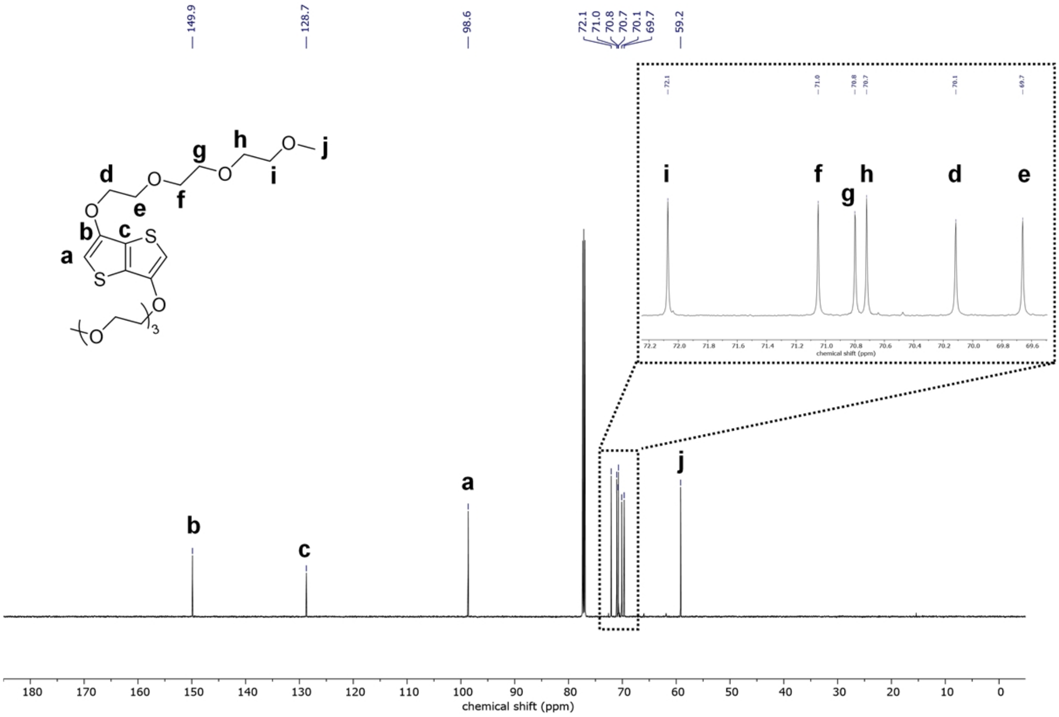

Figure S31. ^{13}C NMR spectrum ($CDCl_3$, 151 MHz, 298K) of 3,6-bis(triethylene glycol monomethyl ether)thieno[3,2-*b*]thiophene

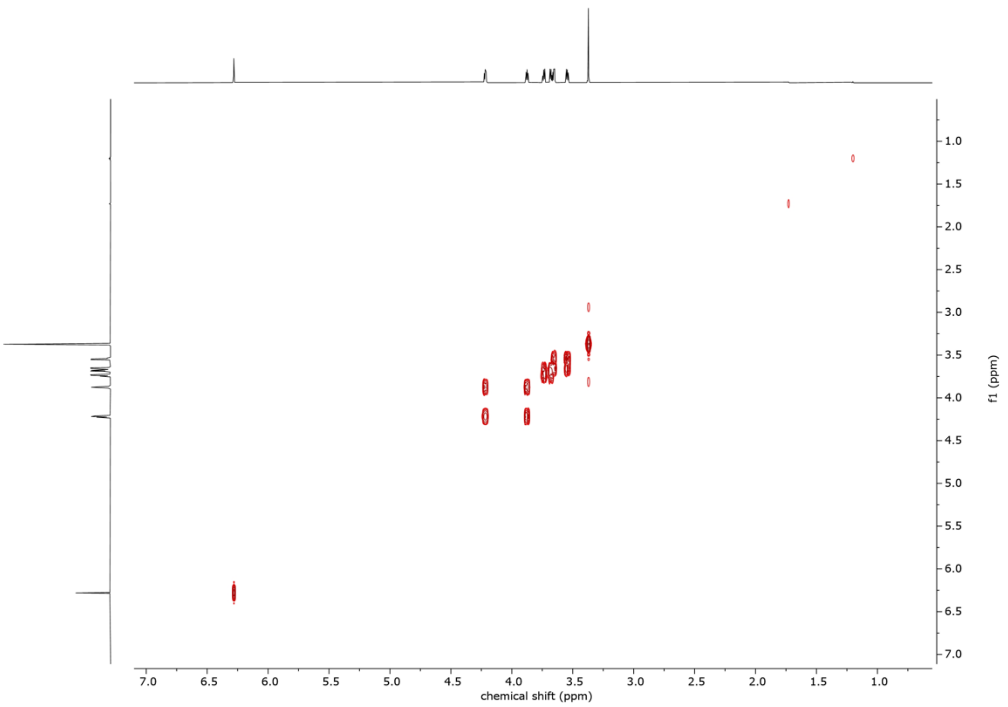

Figure S32. ^{1}H COSY NMR spectrum ($CDCl_3$, 600 MHz, 298K) of 3,6-bis(triethylene glycol monomethyl ether)thieno[3,2-*b*]thiophene

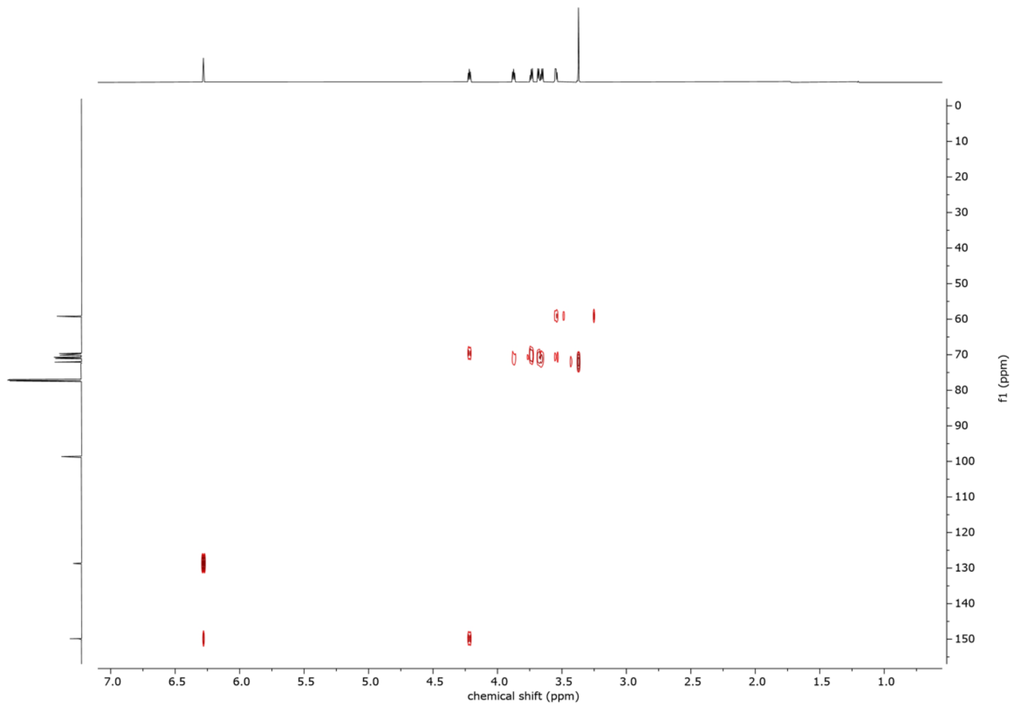

Figure S33. HMBC NMR spectrum ($CDCl_3$, 600/151 MHz, 298K) of 3,6-bis(triethylene glycol monomethyl ether)thieno[3,2-*b*]thiophene

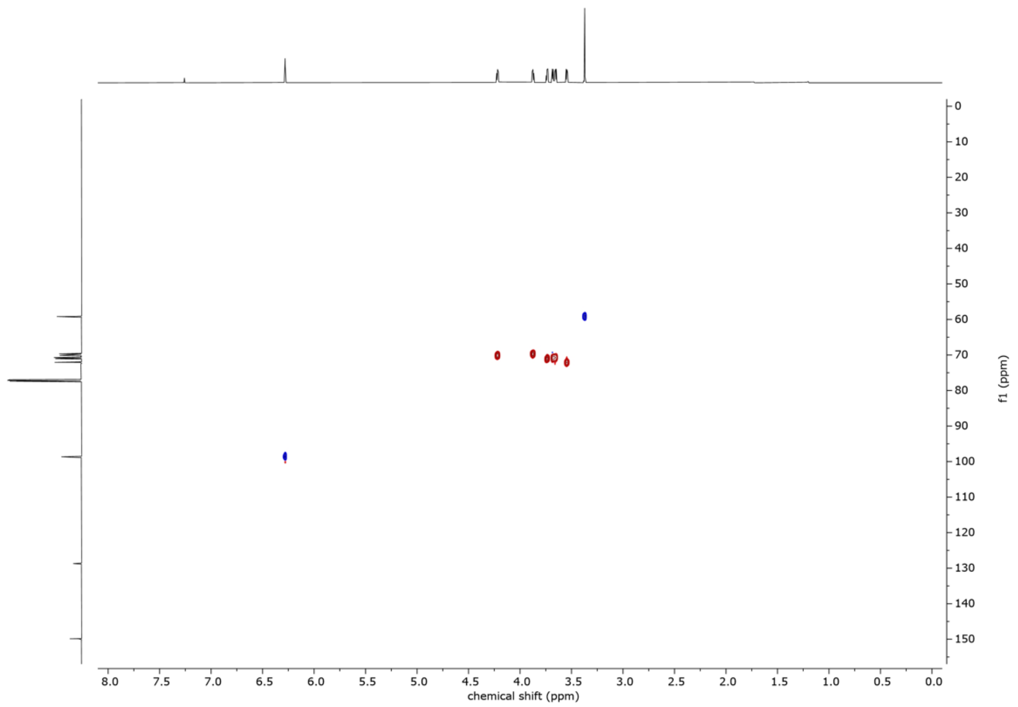

Figure S34. HSQC NMR spectrum ($CDCl_3$, 600/151 MHz, 298K) of 3,6-bis(triethylene glycol monomethyl ether)thieno[3,2-*b*]thiophene

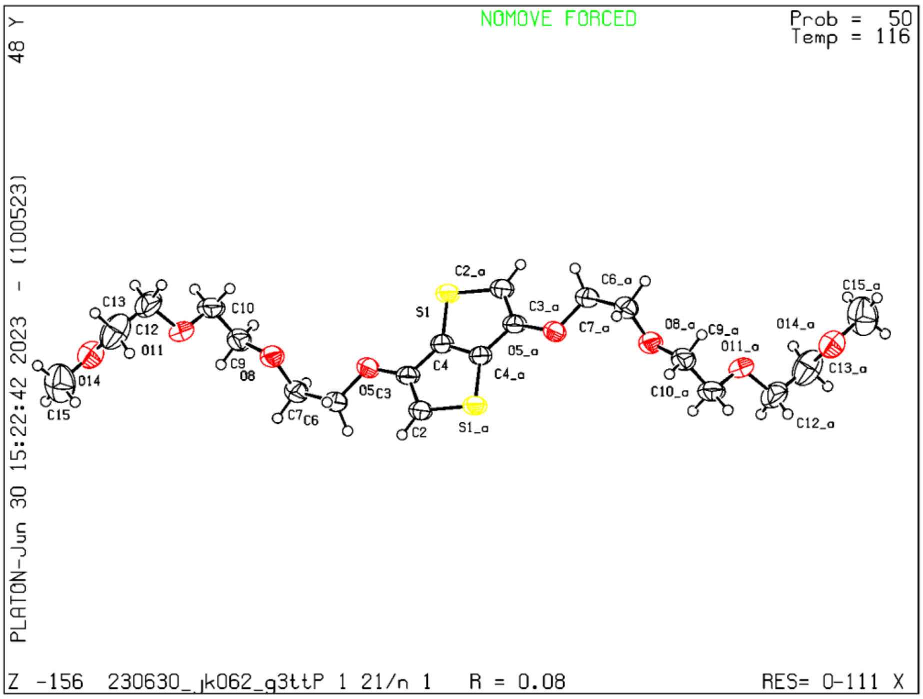

Figure S35. Single crystal X-ray ellipsoid plot of 3,6-bis(triethylene glycol monomethyl ether)thieno[3,2-*b*]thiophene. CCDC deposition number 2336289.

Optimization of glycolation of thieno[3,2-b]thiophene by base change and amine ligand change: Triethylene glycol monomethyl ether (1.34 mL, 8.40 mmol, 10 equiv.) and base [sodium hydroxide (0.336 g, 8.40 mmol, 10 equiv.) or cesium hydroxide monohydrate (1.41 g, 8.40 mmol, 10 equiv.)] were added to a 10 mL high pressure reaction vial and stirred for 1 h at 80 °C under inert atmosphere. After reaction of the glycol with the base, indicated by formation of a deep brown [sodium hydroxide] or deep orange [cesium hydroxide monohydrate] solution, CuI (0.0800 g, 0.420 mmol, 0.50 equiv.), amine ligand (0.840 mmol, 1.0 equiv) and 3,6-dibromothieno[3,2-*b*]thiophene (0.250 g, 0.839 mmol, 1.0 equiv.) were added to the reaction mixture and left to react for 24 h at 100 °C. After 24 h, the reaction mixture was cooled to room temperature and poured into 100 mL chloroform. Purification was performed in a similar way mentioned before. Synthetic results are summarized in Table S3.

Table S3. Monomer synthesis optimization parameters.

Attempt	Base	Amine	Yield
1	*t*BuOK (4 equiv.)	-	47
2	*t*BuOK (8 equiv.)	-	45
3	*t*BuOK (4 equiv.)	Phenanthroline	18
4	*t*BuOK (4 equiv.)	Sarcosine	21
5	$CsOH \bullet H_2O$ (10 equiv.)	-	11
6	NaOH (10 equiv.)	-	8
7	NaOH (10 equiv.)	Phenanthroline	12
8	NaOH (10 equiv.)	Bipyridine	15
9	NaOH (10 equiv.)	Sarcosine	14
10	NaOH (10 equiv.)	Glycine	8
11	NaOH (10 equiv.)	TMEDA	6
12	NaOH (10 equiv.)	TriMEDA	10

Table S4. Monomer optimized synthesis repeat yields. Yield used in synthesis description given in bold.

Attempt	Yield
1	40
2	37
3	33
4	65
5	52
6	53
7	55
8	41
9	37
10	44
11	61
**12**	**47**

S2.4 Polymer synthesis and characterization

To a 10 mL high pressure reaction vial, 3,6-di(triethylene glycol monomethyl ether)thieno[3,2-*b*]thiophene (0.0500 g, 0.108 mmol, 1.0 equiv.), comonomer (0.108 mmol, 1.0 equiv.), pivalic acid (0.0109 g, 0.108 mmol, 1.0 equiv.), and cesium carbonate (0.105 g, 0.323 mmol, 3.0 equiv.) were added and brought under inert atmosphere. After three consecutive cycles of vacuum and inert gas, tris(dibenzylideneacetone)dipalladium(0)-chloroform adduct (3.3 mg, 3.23 μmol, 0.03 equiv.) and toluene (2.16 mL, [monomer] = 50 mM) were added to the reaction tube. This was followed with another three cycles of vacuum and inert gas. Under vigorous stirring, the temperature was raised to 110 °C and kept constant. Reaction progress was monitored by precipitation of aliquots in hexane, methanol, ethyl

acetate and chloroform. Once precipitates formed in hexane, methanol and ethyl acetate, the reaction was considered complete and the mixture was precipitated into hexane. Solids were collected by filtration into a Soxhlet thimble. Soxhlet extraction was performed with hexane, methanol, ethyl acetate and chloroform. The highest molecular weight fraction was concentrated to roughly 50 mL. To this, 50 mL of a 0.5 M sodium diethyldithiocarbamate trihydrate in demineralized water was added and the mixture was stirred vigorously under reflux for 1 h to remove the catalyst. The mixture was poured into a separatory funnel, the organic phase was collected. The organic phase was concentrated and precipitated into hexane. For **P1** (T), **P2** (T2), **P3** (TT), and **P8** (BT) the synthesis was repeated with multiple times. The average yields of all batches are reported in Table 1, specific yields are given in Table S5.

Table S5. Repeated polymer synthesis yields. Highest yields given in bold.

Material	Attempt	Yield
**P1**	1	48
	**2**	**65**
	3	35
	4	42
	5	50
	6	47
**P2**	1	32
	**2**	**33**
	3	25
	4	18
	5	28
	6	30
**P3**	1	34
	**2**	**41**
**P8**	1	31
	**2**	**54**

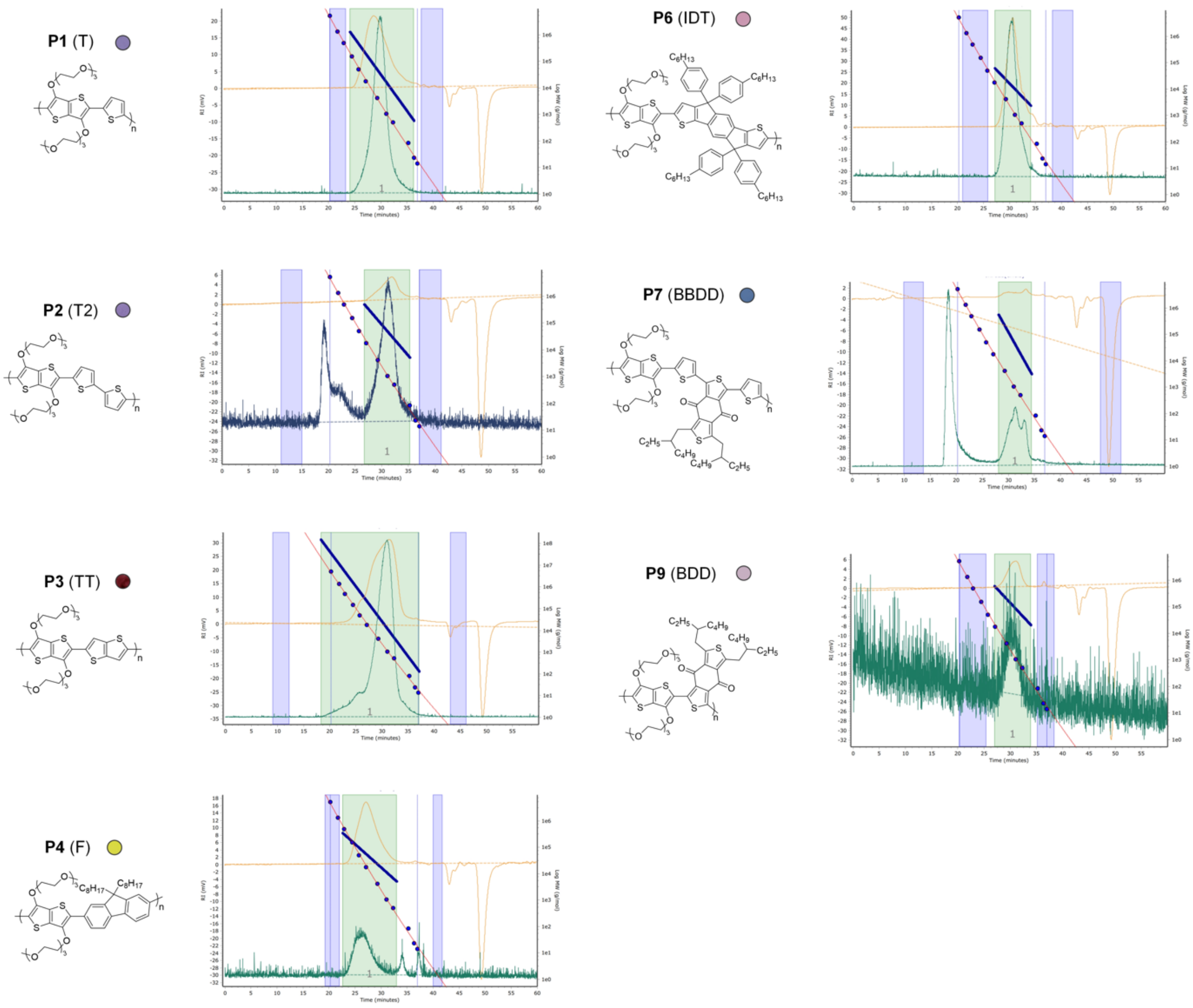

Figure S36. SEC traces of **P1** – **P4**, **P6**, **P7** and **P9** at 343 K in DMF with 0.1 wt% LiBr at a polymer concentration of 1 g L^{-1}. Detectors showcased in traces are refractive index (orange) and light scattering (green/blue).

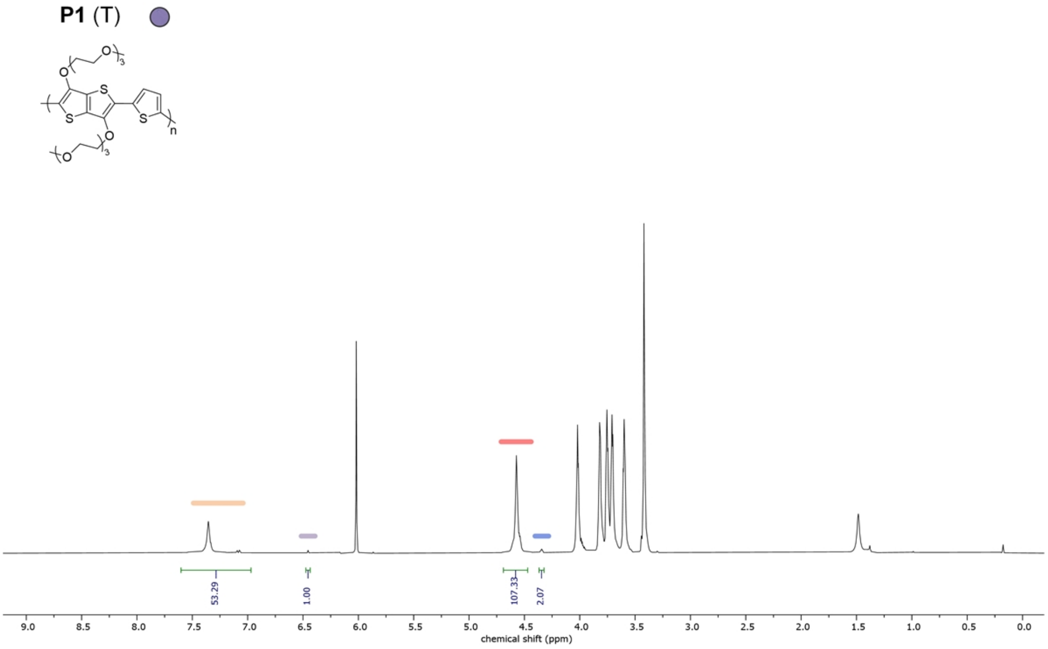

Figure S37. High temperature ^{1}H NMR spectrum ($C_2D_2Cl_4$, 600 MHz, 393K) of **P1** (T) where integrals showcase thiophene aromatic peaks in polymer (orange line), g_3TT aromatic end-group (purple line), g_3TT glycol peaks in polymer chain (red line), and g_3TT glycol end-group (blue line).

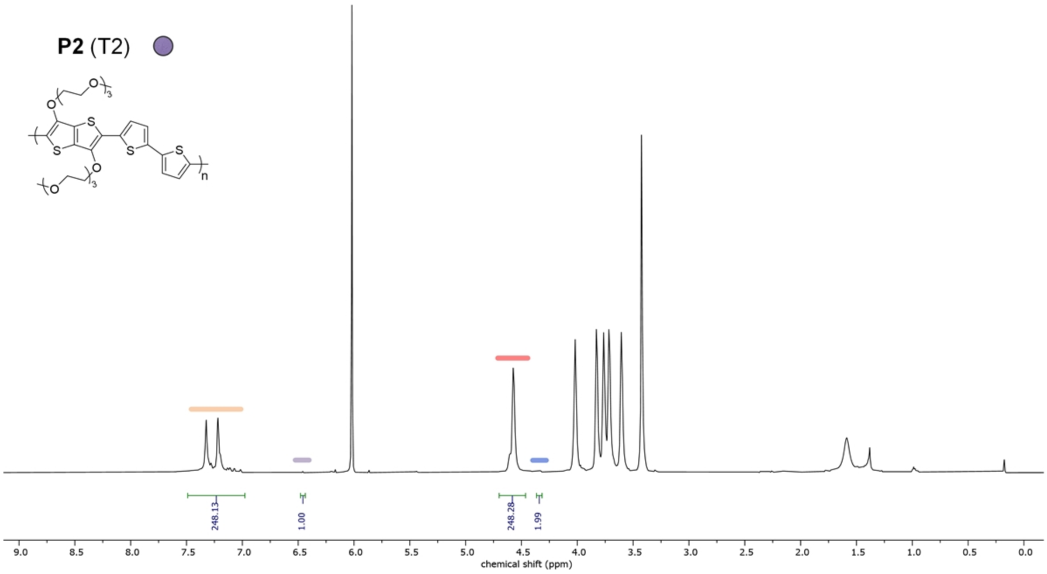

Figure S38. High temperature ^{1}H NMR spectrum ($C_2D_2Cl_4$, 600 MHz, 393K) of **P2** (T2) where integrals showcase bithiophene aromatic peaks in polymer (orange line), g_3TT aromatic end-group (purple line), g_3TT glycol peaks in polymer chain (red line), and g_3TT glycol end-group (blue line).

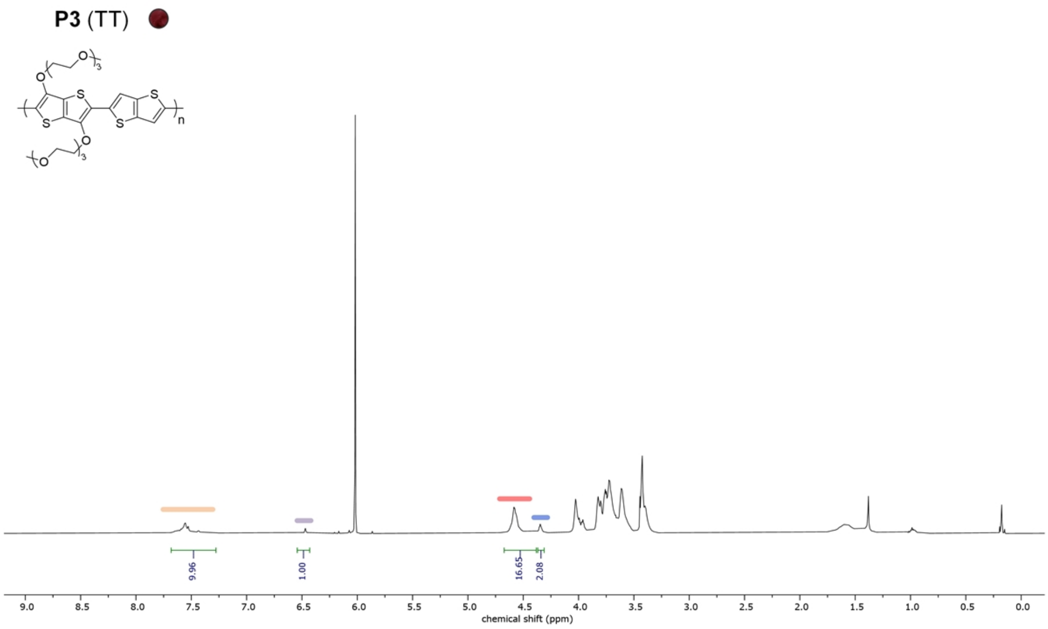

Figure S39. High temperature ^{1}H NMR spectrum ($C_2D_2Cl_4$, 600 MHz, 393K) of **P3** (TT) where integrals showcase thieno[3,2-*b*]thiophene aromatic peaks in polymer (orange line), g_3TT aromatic end-group (purple line), g_3TT glycol peaks in polymer chain (red line), and g_3TT glycol end-group (blue line).

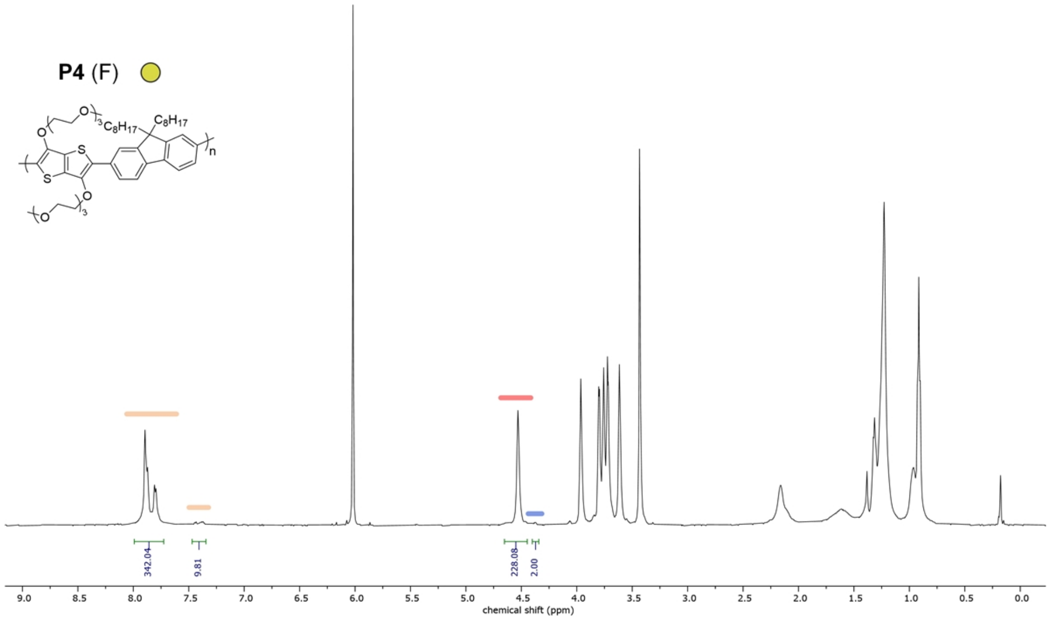

Figure S40. High temperature ^{1}H NMR spectrum ($C_2D_2Cl_4$, 600 MHz, 393K) of **P4** (F) where integrals showcase fluorene aromatic peaks in polymer (orange line), g_3TT glycol peaks in polymer chain (red line), and g_3TT glycol end-group (blue line).

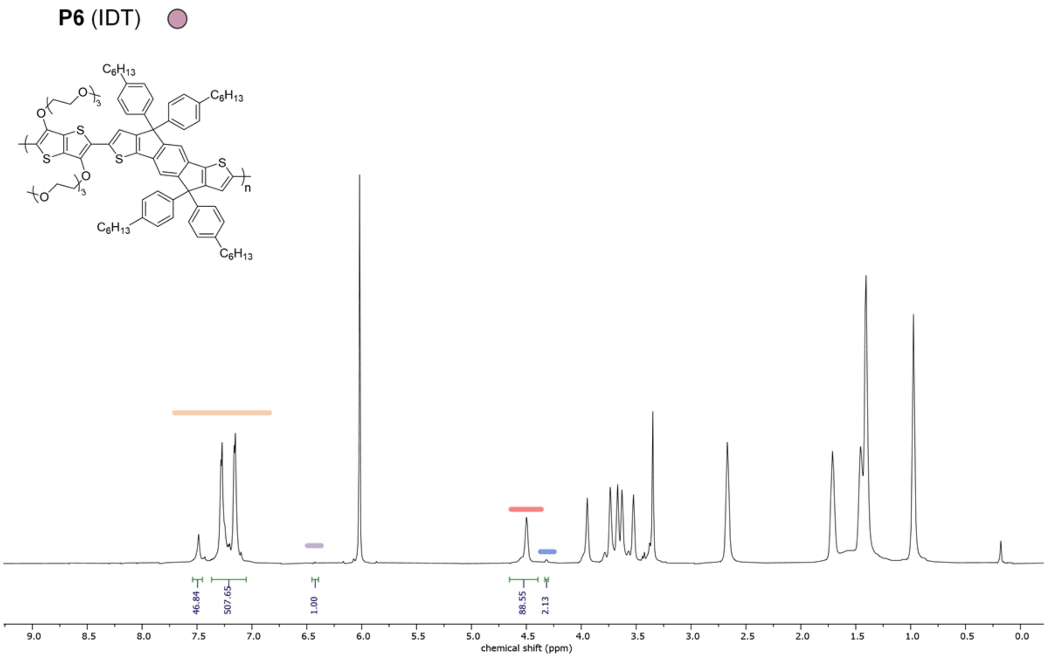

Figure S41. High temperature ^{1}H NMR spectrum ($C_2D_2Cl_4$, 600 MHz, 393K) of **P6** (IDT) where integrals showcase indacenodithiophene aromatic peaks in polymer (orange line), g_3TT aromatic end-group (purple line), g_3TT glycol peaks in polymer chain (red line), and g_3TT glycol end-group (blue line).

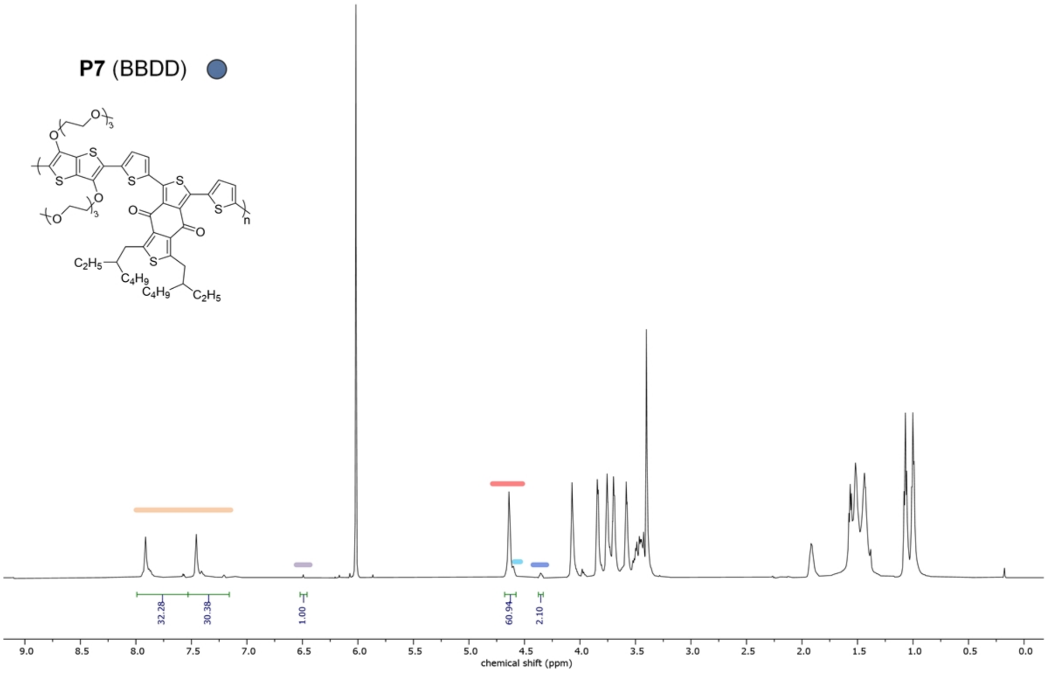

Figure S42. High temperature ^{1}H NMR spectrum ($C_2D_2Cl_4$, 600 MHz, 393K) of **P8** (BBDD) where integrals showcase bisthiophenylbenzodithiophenedione aromatic peaks in polymer (orange line), g_3TT aromatic end-group (purple line), g_3TT glycol peaks in polymer chain (red line), and g_3TT glycol end-groups (turquoise and blue lines).

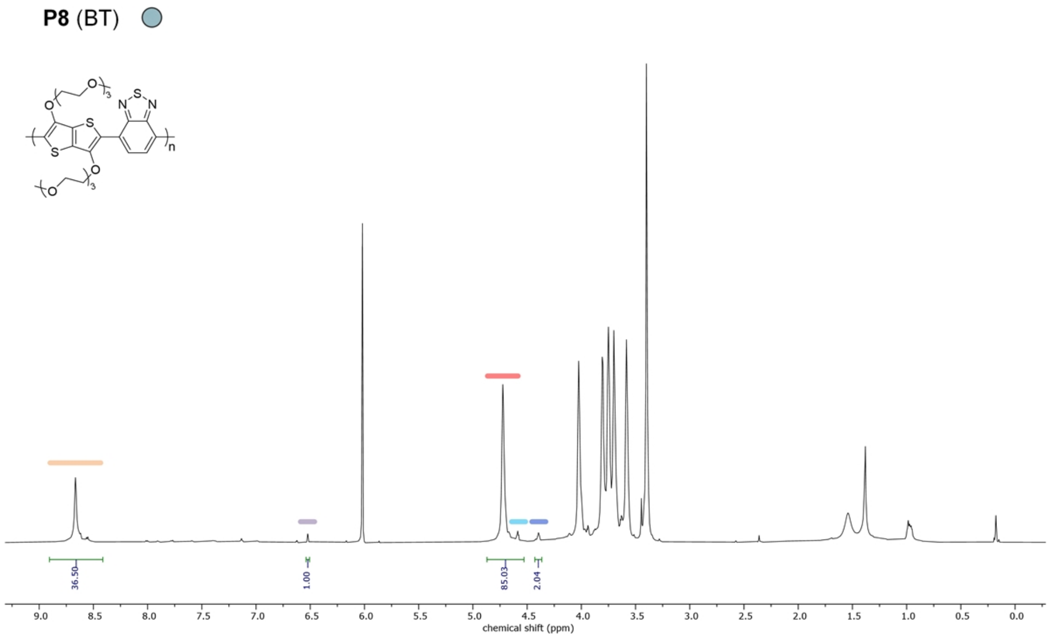

Figure S43. High temperature ^{1}H NMR spectrum ($C_2D_2Cl_4$, 600 MHz, 393K) of **P8** (BT) where integrals showcase benzothiadiazole aromatic peaks in polymer (orange line), g_3TT aromatic end-group (purple line), g_3TT glycol peaks in polymer chain (red line), and g_3TT glycol end-group (turquoise and blue lines).

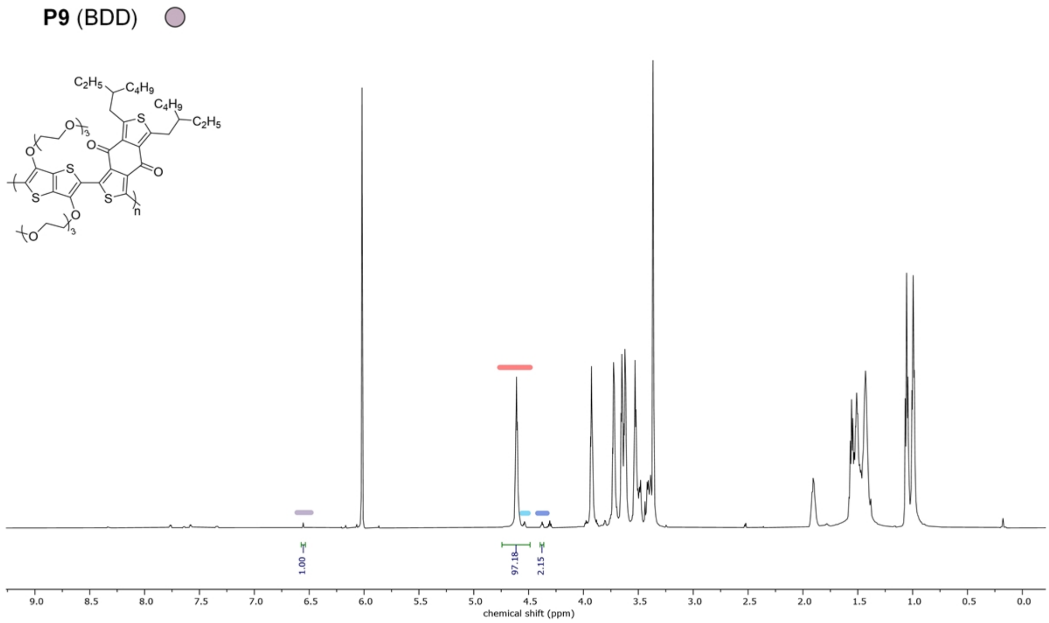

Figure S44. High temperature ^{1}H NMR spectrum ($C_2D_2Cl_4$, 600 MHz, 393K) of **P8** (BDD) where integrals showcase g_3TT aromatic end-group (purple line), g_3TT glycol peaks in polymer chain (red line), and g_3TT glycol end-group (turquoise and blue lines).

S3 Polymer molecular weight determination by NMR

S3.1 Derivation

Using Figure 3 from the main text (shown on the right here), the number-average molecular weight of the polymer can be determined by using high temperature NMR spectroscopy in tetrachloroethane-d_2. Aromatic peaks are expected above 6.25 ppm and glycol peaks are expected between 3.30 ppm and 4.75 ppm, based on NMRs of previously reported glycolated aromatic systems.[4, 9, 13, 14]

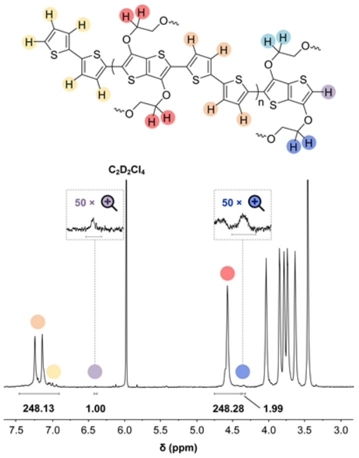

In the final calculation of number-average molecular weight, we assume a statistical mixture of end-groups of the C-H activated monomer (here, g_3TT) and the brominated monomer which has undergone debromination (here, T2) as determined by MALDI-ToF (Figure S45). The end-group of g_3TT possesses one indicative aromatic C-H group (circa 6.50 ppm, purple in Figure). This signal showcases an integral ratio of 1:2 with the CH_2 from the glycol chains closest to the thieno[3,2-*b*]thiophene unit (ca. 4.30 ppm, dark blue in Figure 3). The end-group possesses another CH_2 from the other glycol chain (ca. 4.55 ppm, turquoise in Figure) which has merged with the CH_2 signals from the main chain glycol chain (ca. 4.60 ppm, red in Figure) in **P2**. This signal is visible for **P7-P9** in Figures S14 and S15, though still in the shoulder region of the main chain. Both signals come from the glycol chains of the end-group, they must be the same integral. Accordingly, we make the assumption in equation S3.

The main chain molecular weight was found by comparing the integral of the glycol end-group signal(s) against the integral of the glycol in the main chain and subsequently multiplying with the molecular weight of the repeat unit. The end-group integral must be subtracted from the main chain integral due to the overlap of the end-group at ca. 4.55 ppm.

Moreover, the molecular weight of one repeat unit and HBr must be added to this molecular weight, which are representative of the actual end-group. Equation S4 summarizes this notion and equation S5 showcases the calculation, brackets representing different parts.

$$\int I_{end}\, d\delta \cong \int I'_{end}\, d\delta \tag{S3}$$

$$M_{n,NMR} = [\![main\ chain]\!] + [end - group] + < end - group\ H\ on\ both\ ends > \tag{S4}$$

$$M_{n,NMR} = \left[\!\!\left[\left(\frac{\int I_{chain}\, d\delta - \int I'_{end}\, d\delta}{\int I_{end}\, d\delta + \int I'_{end}\, d\delta} \right) MW_{repeat} \right]\!\!\right] + [MW_{repeat}] + < MW_{H_2} > \tag{S5}$$

where $\int I_{end}\, d\delta$ is the integral of the glycol end-group signal oriented to the end of the chain (ca. 4.30 ppm), $\int I'_{end}\, d\delta$ is the integral of the glycol end-group signal oriented to the polymer chain (ca. 4.55 ppm), $\int I_{chain}\, d\delta$ is the integral of the main chain glycol signal (ca. 4.60 - 4.70 ppm), MW_{repeat} is the molecular weight of the repeat unit, and MW_{H_2} is the molecular weight of the hydrogens at the ends of the polymer chain.

Equation S3 can be used to derive equation S6 from S5 which ultimately simplifies to equation S7 (equation 1 in main text).

$$M_{n,NMR} = \left(\frac{\int I_{chain}\, d\delta - \int I_{end}\, d\delta}{2\int I_{end}\, d\delta} + 1 \right) MW_{repeat} + MW_{H_2} \tag{S6}$$

$$M_{n,NMR} = \frac{MW_{repeat}}{2}\left(\frac{\int I_{chain}\, d\delta}{\int I_{end}\, d\delta} + 1\right) + MW_{H_2} \tag{S7}$$

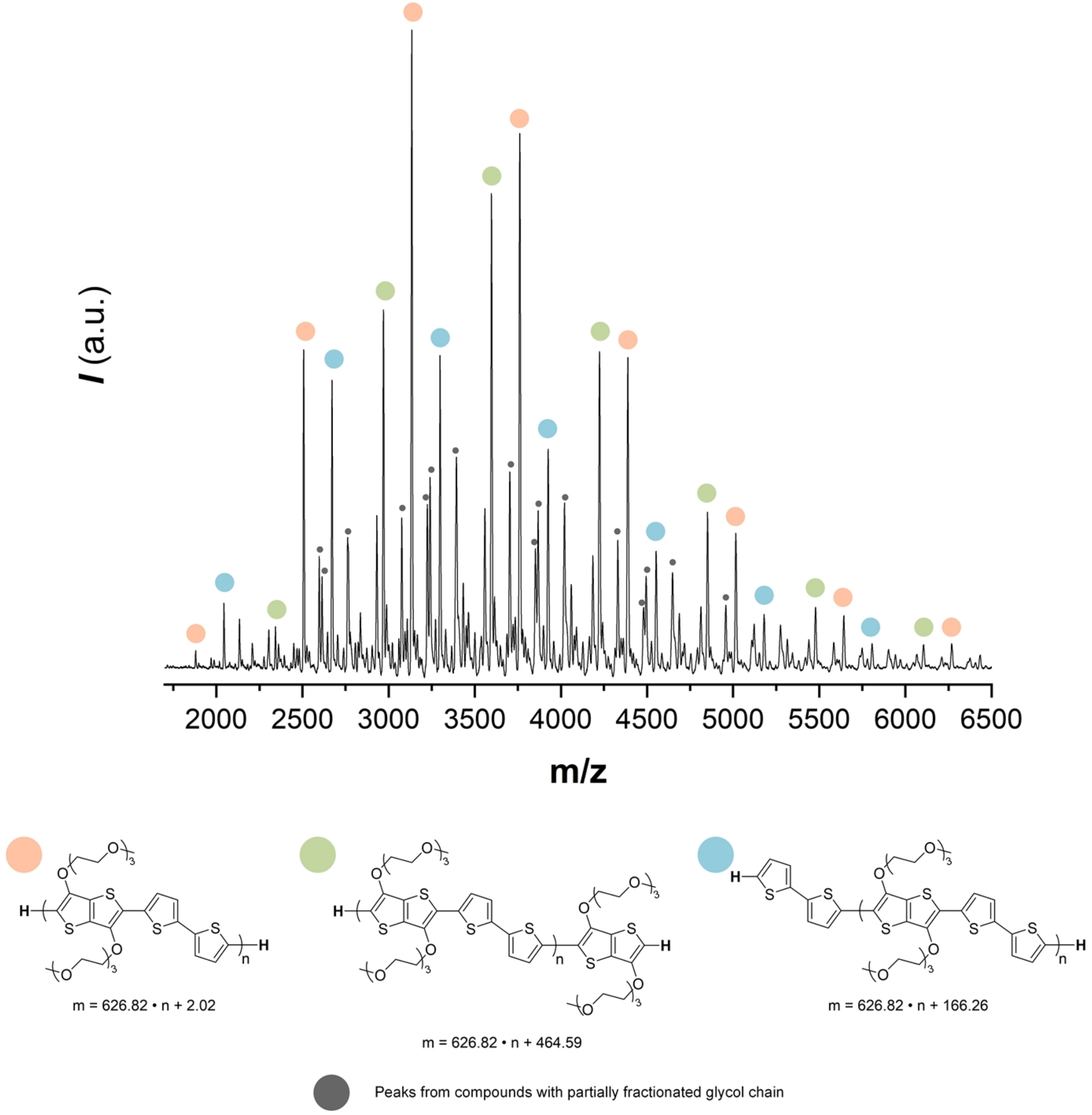

Figure S45. Representative MALDI-ToF spectrum of **P2**. Minor peaks, shown in black, are oligoether fractionation peaks (parts of the side chain have fractionated to leave a lighter ions).

S3.2 Uncertainties in molecular weight from NMR

An uncertainty in integral was estimated using MestReNova's built-in signal-to-noise (S/N) tool and equation S8. We assume that peak height uncertainty extends to integration uncertainty by using consistent spectral widths of signal and noise. Accordingly, a relative uncertainty in the integral ratio is calculated by basic quadrature analysis:

$$\frac{|\Delta R|}{R} = \sqrt{\frac{1}{(S/N_{chain})^2} + \frac{1}{(S/N_{end})^2}} \tag{S8}$$

where $\frac{|\Delta R|}{R}$ is the relative uncertainty, S/N_{chain} is the signal-to-noise of the main chain signal, and S/N_{end} is the signal-to-noise of the end-group signal.

**P1** (T)	$\frac{|\Delta R|}{R} = \sqrt{\frac{1}{372^2} + \frac{1}{37^2}} = 0.027 \rightarrow 2.7\%$
**P2** (T2)	$\frac{|\Delta R|}{R} = \sqrt{\frac{1}{363^2} + \frac{1}{6.5^2}} = 0.15 \rightarrow 15\%$
**P3** (TT)	$\frac{|\Delta R|}{R} = \sqrt{\frac{1}{161^2} + \frac{1}{57^2}} = 0.019 \rightarrow 1.9\%$
**P4** (F)	$\frac{|\Delta R|}{R} = \sqrt{\frac{1}{319^2} + \frac{1}{7.8^2}} = 0.13 \rightarrow 13\%$
**P6** (IDT)	$\frac{|\Delta R|}{R} = \sqrt{\frac{1}{136^2} + \frac{1}{11^2}} = 0.091 \rightarrow 9.1\%$
**P7** (BBDD)	$\frac{|\Delta R|}{R} = \sqrt{\frac{1}{178^2} + \frac{1}{10^2}} = 0.091 \rightarrow 10\%$
**P8** (BT)	$\frac{|\Delta R|}{R} = \sqrt{\frac{1}{828^2} + \frac{1}{52^2}} = 0.019 \rightarrow 1.9\%$
**P9** (BBD)	$\frac{|\Delta R|}{R} = \sqrt{\frac{1}{504^2} + \frac{1}{21^2}} = 0.048 \rightarrow 4.8\%$

S3.3 Molecular weight calculations for all polymers

In reference to NMRs shown in Figure S36 – S43, molecular weights have been determined using equation S7 (i.e. equation 1 from the main text) and adding the uncertainties obtained from equation S8.

**P1** (T)	$M_{n,NMR} = \frac{544.7}{2}\left(\frac{107.33}{2.07}+1\right)+2.02 = 14396\ g\ mol^{-1}\ \pm 2.7\% \approx 14\ \pm 0.4\ kg\ mol^{-1}$
**P2** (T2)	$M_{n,NMR} = \frac{626.8}{2}\left(\frac{248.28}{1.99}+1\right)+2.02 = 39416\ g\ mol^{-1} \pm 15\% \approx 39\ \pm 6\ kg\ mol^{-1}$
**P3** (TT)	$M_{n,NMR} = \frac{600.8}{2}\left(\frac{16.65}{2.08}+1\right)+2.02 = 2707\ g\ mol^{-1}\ \pm 1.9\% \approx 3\ \pm 0.1\ kg\ mol^{-1}$
**P4** (F)	$M_{n,NMR} = \frac{851.2}{2}\left(\frac{228.08}{2.00}+1\right)+2.02 = 48963\ g\ mol^{-1} \pm 13\% \approx 49\ \pm 6\ kg\ mol^{-1}$
**P6** (IDT)	$M_{n,NMR} = \frac{1368.0}{2}\left(\frac{88.55}{2.13}+1\right)+2.02 = 29122\ g\ mol^{-1} \pm 9.1\% \approx 29\ \pm 3\ kg\ mol^{-1}$
**P7** (BBDD)	$M_{n,NMR} = \frac{1069.5}{2}\left(\frac{60.94}{2.10}+1\right)+2.02 = 16055\ g\ mol^{-1} \pm 10\% \approx 16\ \pm 2\ kg\ mol^{-1}$
**P8** (BT)	$M_{n,NMR} = \frac{596.8}{2}\left(\frac{85.03}{2.04}+1\right)+2.02 = 12738\ g\ mol^{-1} \pm 1.9\% \approx 13\ \pm 0.2\ kg\ mol^{-1}$
**P9** (BBD)	$M_{n,NMR} = \frac{905.2}{2}\left(\frac{97.18}{2.15}+1\right)+2.02 = 20912\ g\ mol^{-1} \pm 4.8\% \approx 21\ \pm 1\ kg\ mol^{-1}$

S4 Material properties of polymers

S4.1 Optical properties

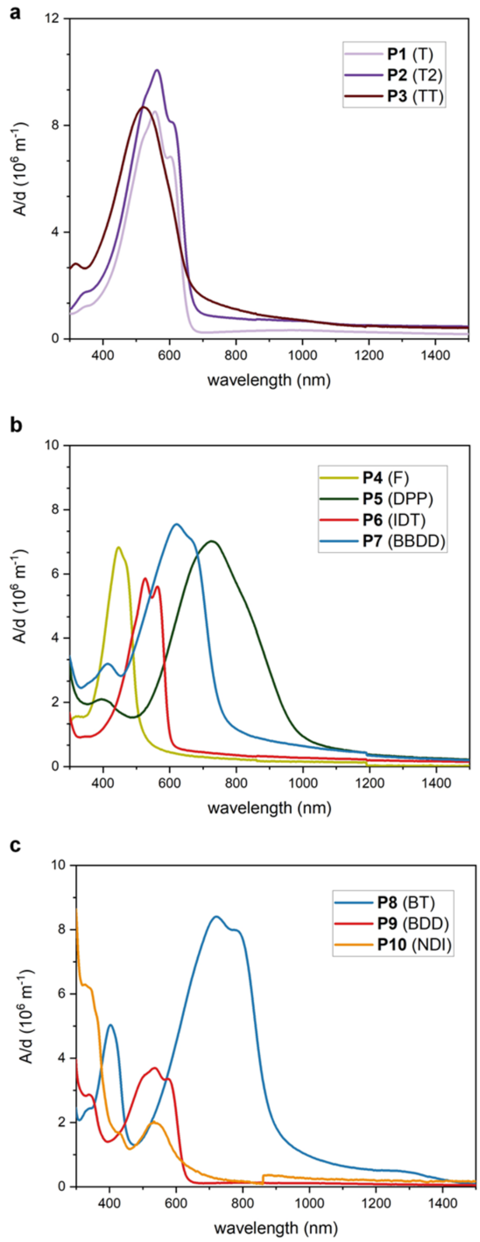

Figure S46. Thickness-normalized UV-vis (absorbance *A* divided by film thickness *d*) of g_3TT copolymers where comonomers are a) donors, b) neutral or ambipolar, and c) acceptors. Spectrum of **P10** contains an artefact of the switching lamps in the spectrometer due to the low thickness of the film.

S4.2 Grazing incidence wide angle X-ray scattering patterns

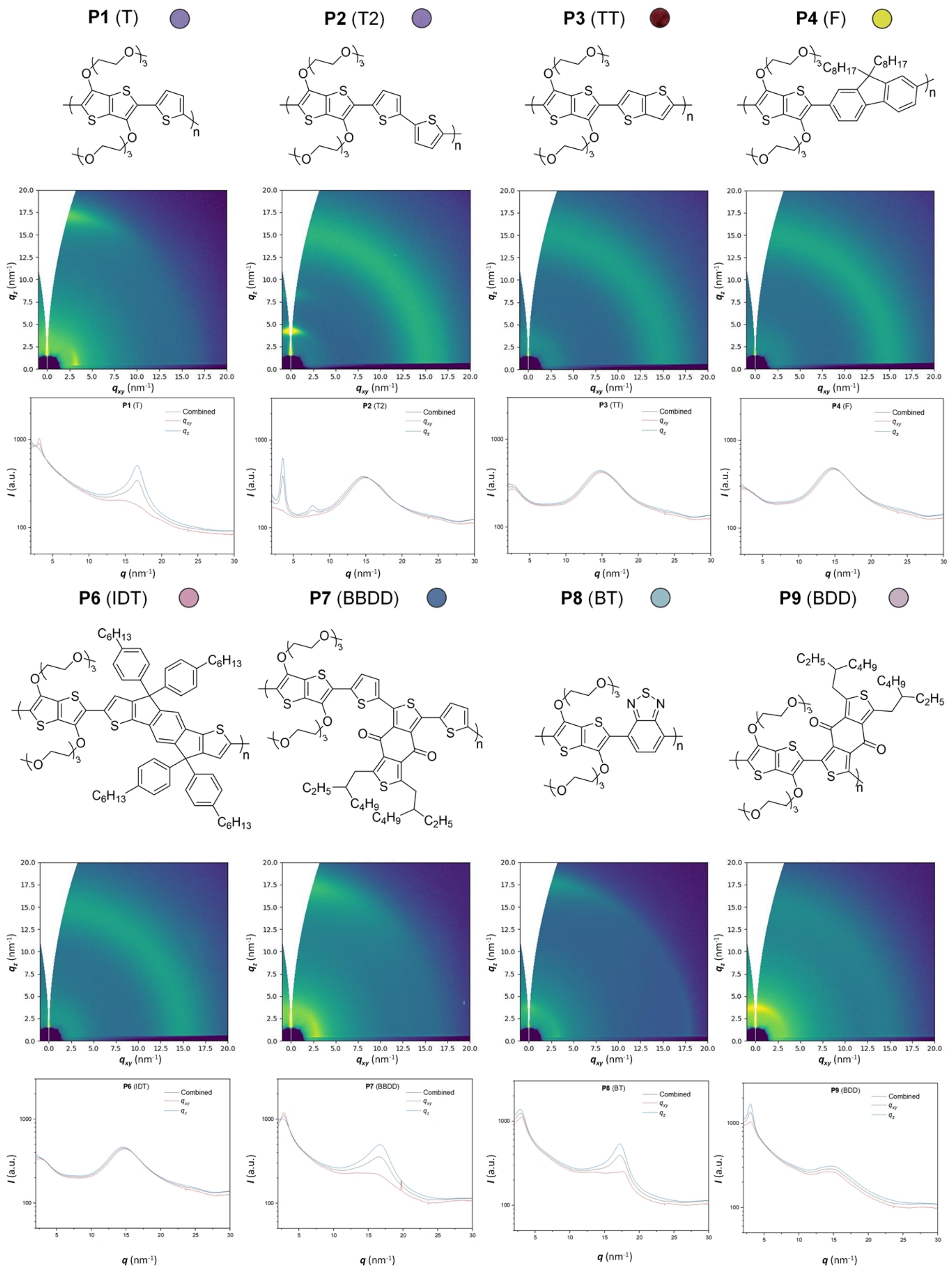

Figure S47. GIWAXS patterns and line cuts of **P1** – **P4** and **P6** – **P9**.

S4.3 Thermogravimetric analysis thermograms

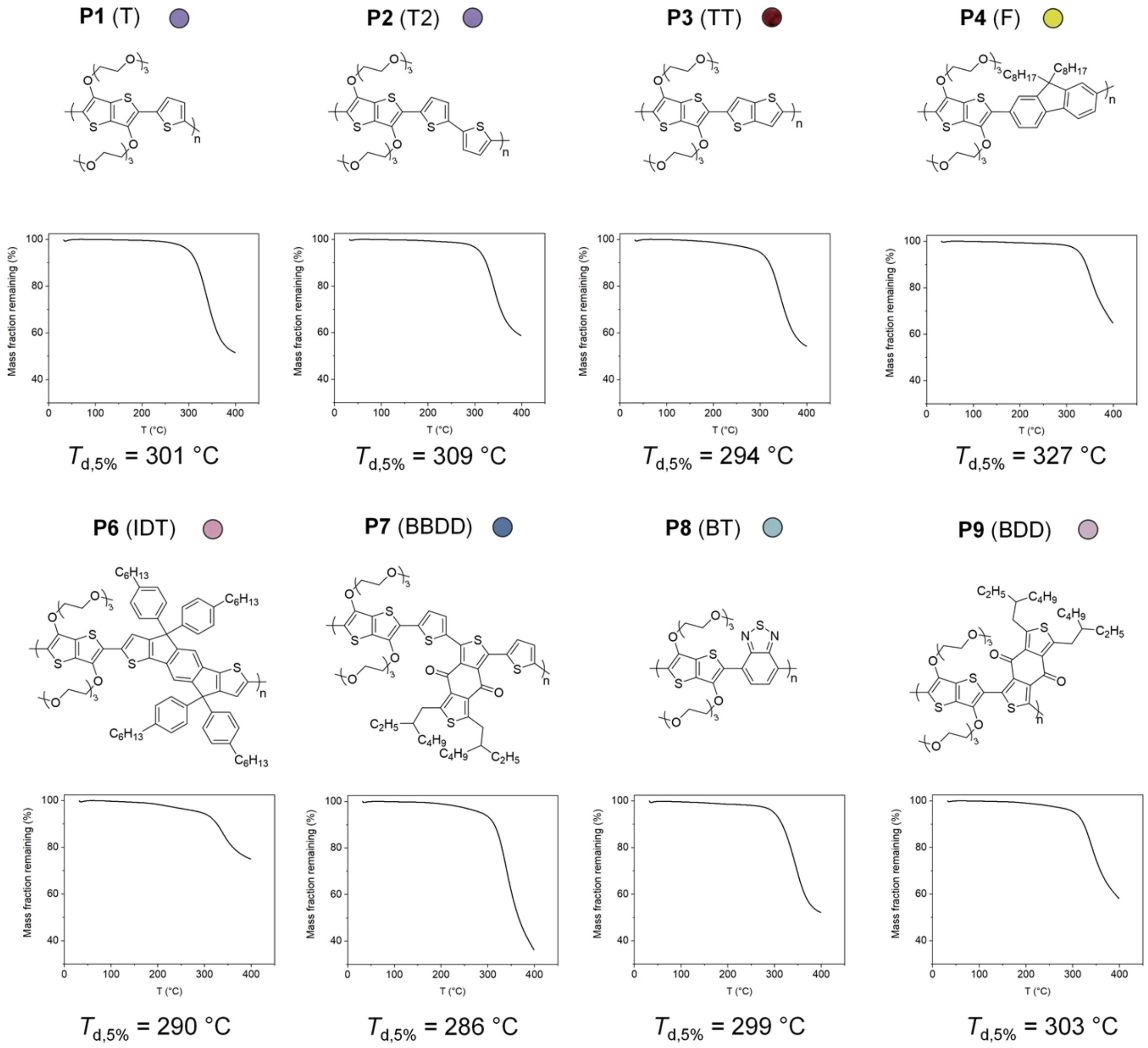

Figure S48. Thermogravimetric analysis thermograms of **P1** – **P4** and **P6** – **P9**, samples were heated from 25 °C to 450 °C (flow rate = 60 mL min^{-1}) using a heating/cooling rate of 10 °C min^{-1}.

S4.4 Electronic properties

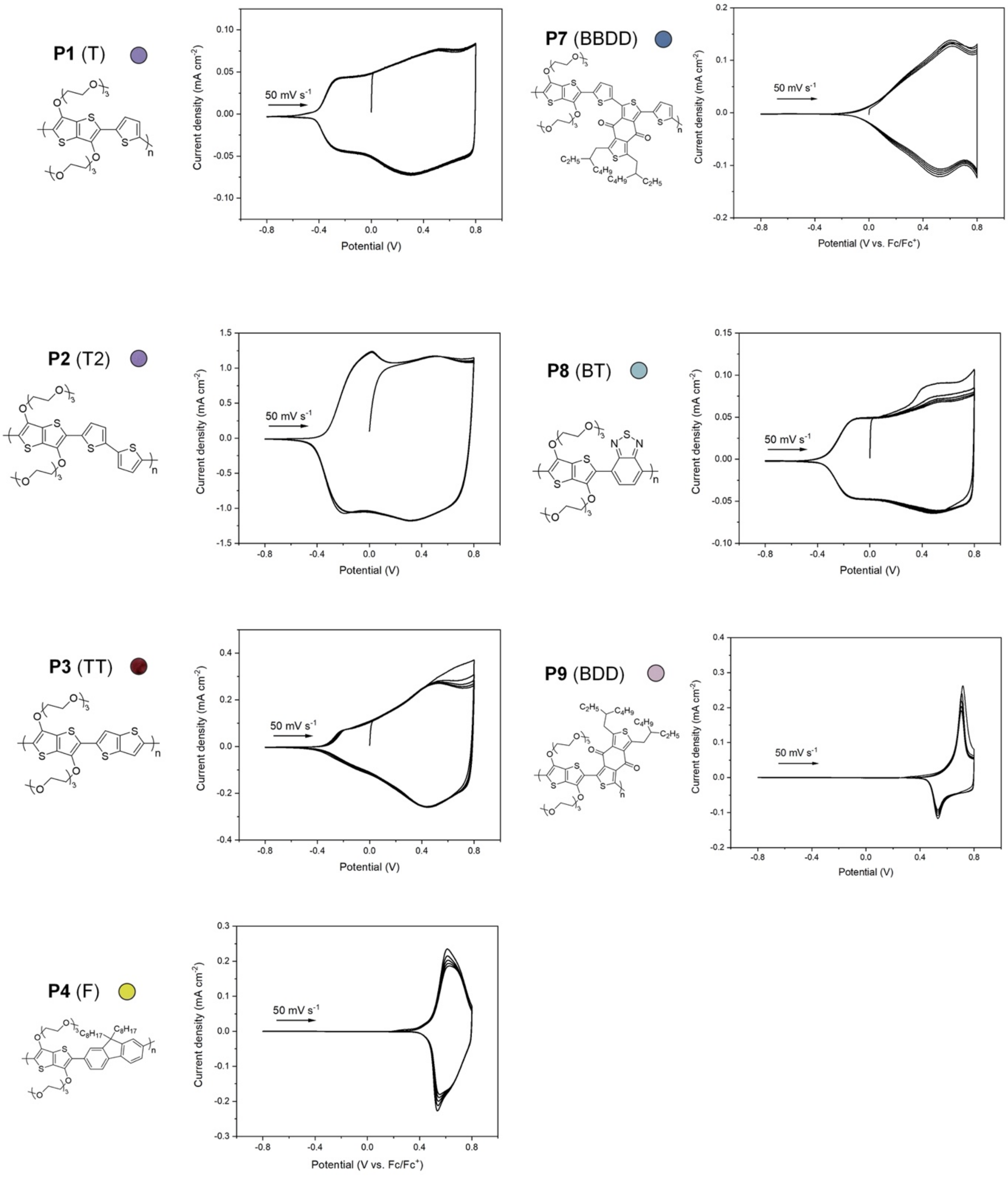

Figure S49. Cyclic voltammograms measured in 0.1 M NBu_4PF_6 in acetonitrile of thin films of **P1** – **P4** and **P7** – **P9**, five consecutive cycles.

Polymer	Pristine	Oxidized	Reduced (after oxidation)
**P1** (T)	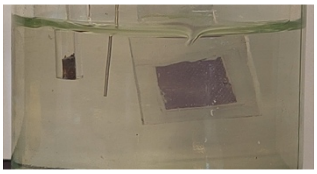	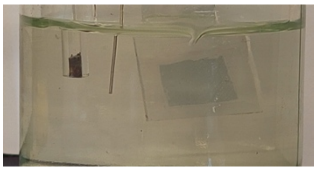	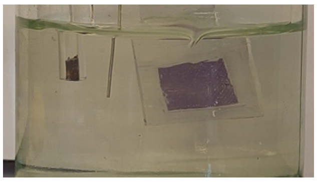
**P2** (T2)	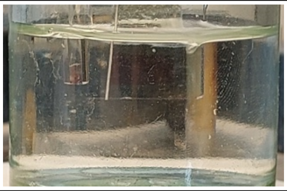	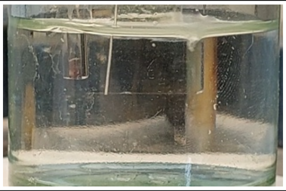	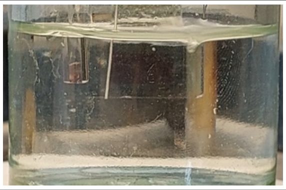
**P3** (TT)	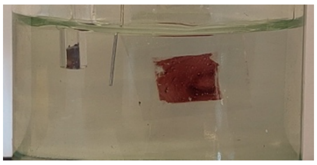	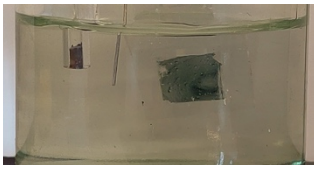	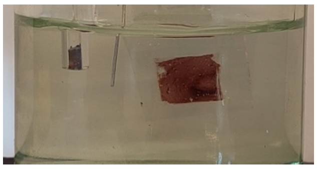
**P4** (F)	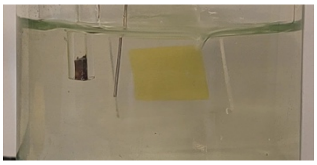	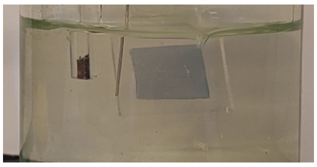	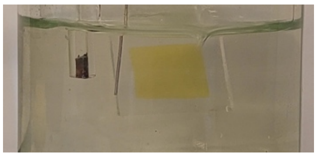
**P7** (BBDD)	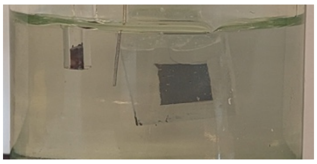	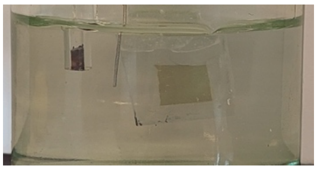	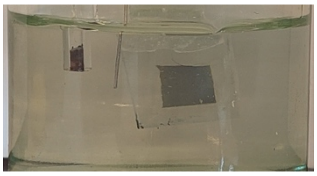
**P8** (BT)	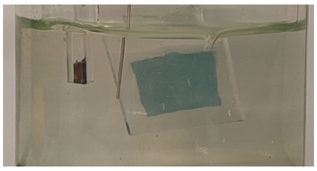	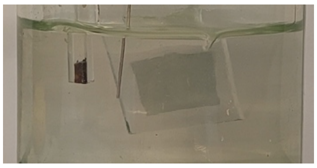	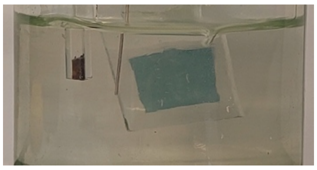
**P9** (BBD)	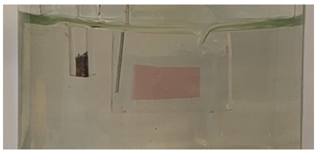	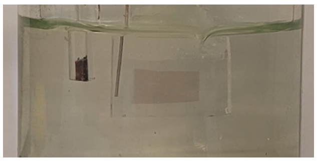	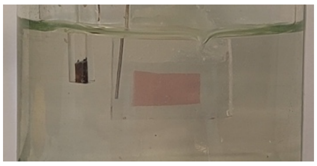

Figure S50. Pictures of pristine, oxidized, and reduced polymer thin films observed in cyclic voltammetry measurements in 0.1 M NBu_4PF_6 in acetonitrile.

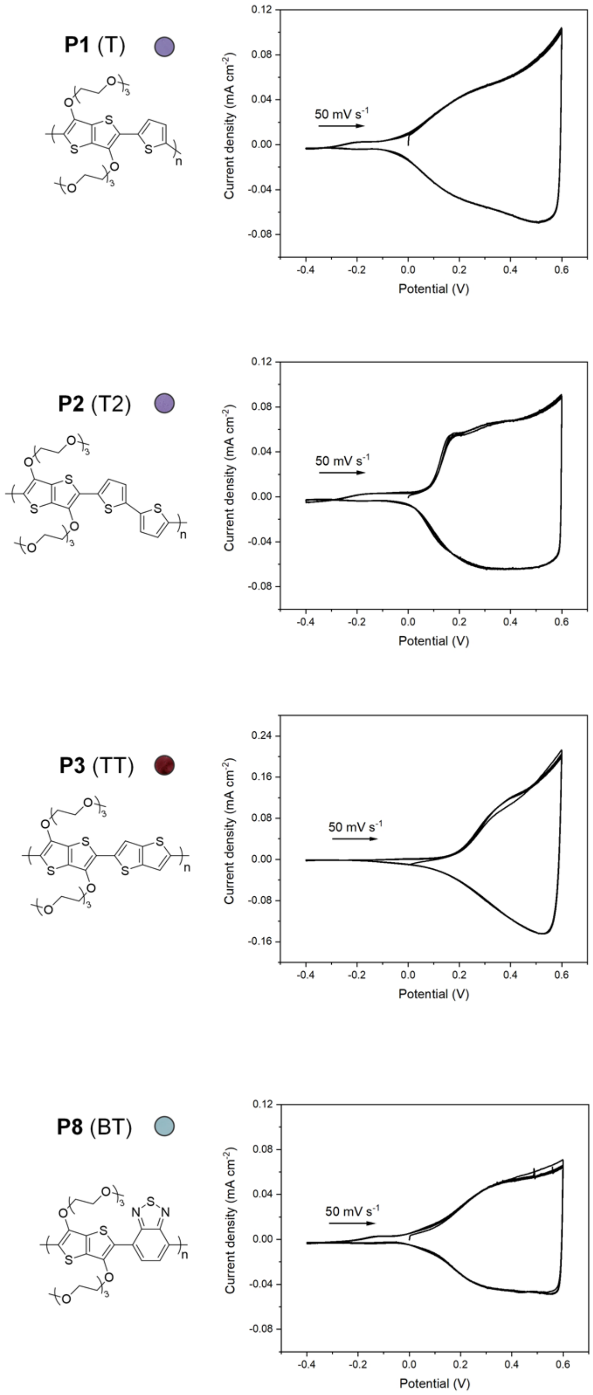

Figure S51. Cyclic voltammograms measured in 0.1 M NaCl in H_2O of thin films of **P1**, **P2**, **P3**, and **P8** recorded, five consecutive cycles.

Table S6. Optoelectronic properties of polymers. [a] Measured against Ag/AgCl from -0.4 to 0.6V. [b] Measured against Fc/Fc$^+$ from -0.8 to 0.8V. [c] From UV-vis. [d] Non-permeable in electrolyte within measured potential range. [e] Upon oxidation polymer dissolved in ACN indicative of degradation.

Polymer	E_{ox,H_2O} (eV)[a]	$E_{ox,AcN}$ (eV)[b]	E_g (eV)[c]
**P1** (T)	4.35	4.69	1.86
**P2** (T2)	4.52	4.77	1.90
**P3** (TT)	4.62	4.74	1.81
**P4** (F)	n.a.[d]	5.59	2.42
**P5** (DPP)	n.a.[d]	5.23	1.26
**P6** (IDT)	n.a.[d]	n.a.[e]	2.06
**P7** (BBDD)	n.a.[d]	5.05	1.63
**P8** (BT)	4.48	4.77	1.37
**P9** (BDD)	n.a.[d]	5.72	1.98
**P10** (NDI)	n.a.[d]	n.a.[e]	1.98

Table S7. Crystalline characteristics of selected copolymers.

Polymer	d_{100} (nm)	L_{100} (nm)	d_{010} (nm)	L_{010} (nm)	d_{halo} (nm)	Orientation
**P1** (T)	1.98	9.57	0.38	2.72	-	Face-on
**P2** (T2)	1.78	10.36	-	-	0.42	Edge-on
**P3** (TT)	-	-	-	-	0.42	-
**P4** (F)	-	-	-	-	0.42	-
**P6** (IDT)	-	-	-	-	0.43	-
**P7** (BBDD)	2.20	3.89	0.38	1.74	-	Face-on
**P8** (BT)	2.12	4.25	0.3.6	2.65	-	Face-on
**P9** (BDD)	2.07	6.12	-	-	0.42	Edge-on

S5 Device properties of polymers

S5.1 OECT output characterization curves

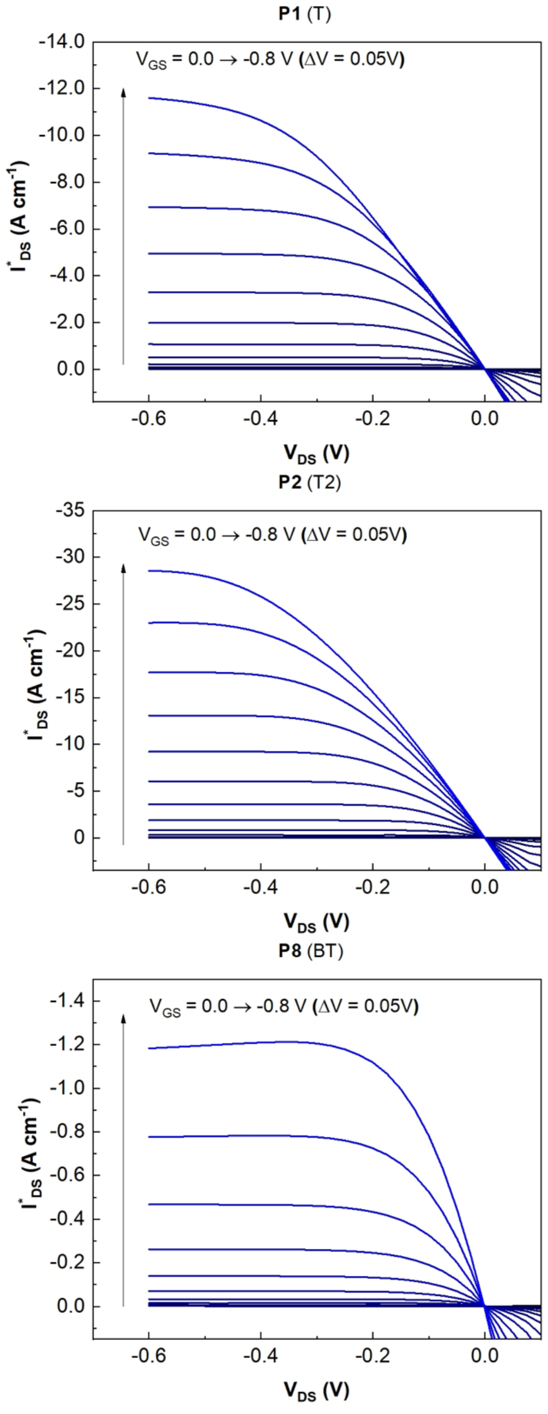

Figure S52. Output characteristic curves of P1, P2 and P8. V_{GS} was scanned from +0.1 to -0.6 V with V_{DS} stepped from 0.0 V (black) to -0.8 V (blue) in 0.05 V interval.

S5.2 Electrochemical impedance spectroscopy characterization curves

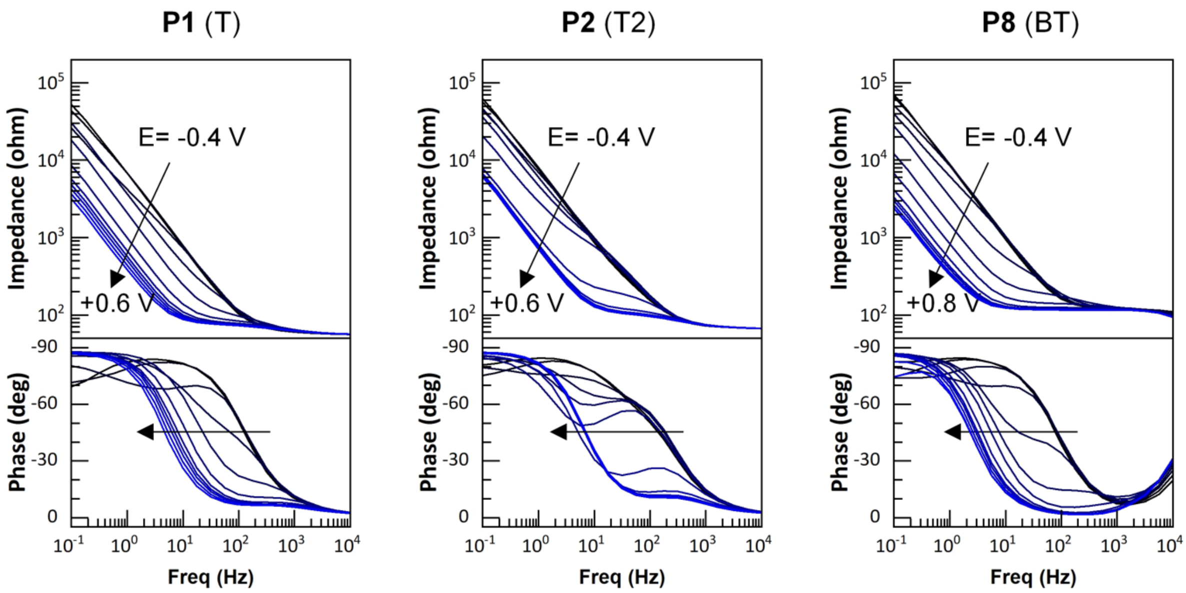

Figure S53. Electrochemical impedance spectroscopy of P1, P2 an P8. The offset potential of the working electrode (*E*) was scanned from -0.4 V to +0.6 V (vs. Ag/AgCl, for P1 and P2) and +0.8 V (for P8) in 0.1 V interval.

S5.3 OECT cycling stability curves

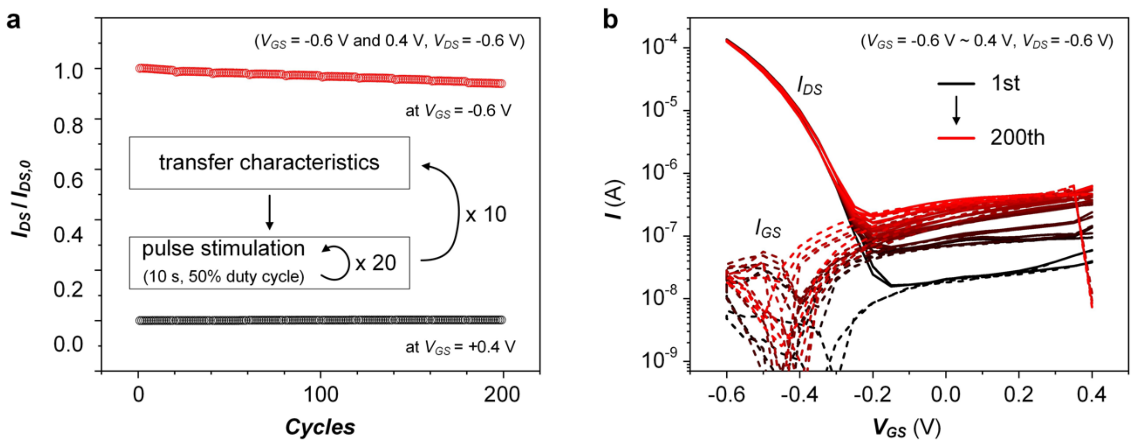

Figure S54. OECT cyclic stability of P2 polymer. (a) Relative on- (red)/off-current (black) under pulsed gate potential (V_{GS}= -0.6 V and +0.4 V at V_{DS} = -0.6 V) for 200 cycles. Inset depicts a flowchart for the cyclic test. (b) Transfer curves at every 20-pulse cycle.

S6 Computational results

Table S8. Summarized computational results for all polymers. Calculations performed on trimer structures (six monomers) with ωB97XD functional at 6-31+G(d,p) level of theory. [a] $0° < \varphi < 90°$. * annotates dihedral angles between aromatic rings within monomers, e.g. between thiophenes in bithiophene. The inset underneath the table shows the orbital coefficients of the frontier orbitals of **P8**.

Polymer	$\varphi_{DFT}{}^{a}$ (°)	$E_{HOMO,DFT}$ (eV)
**P1** (T)	26.9 ± 11.3	-6.82
**P2** (T2)	13.1 ± 0.8<br>*28.1 ± 1.4	-6.78
**P3** (TT)	14.9 ± 0.9	-6.68
**P4** (F)	45.6 ± 1.8	-7.28
**P5** (DPP)	6.7 ± 0.6<br>*25.5 ± 1.3	-6.66
**P6** (IDT)	15.6 ± 1.0	-6.39
**P7** (BBDD)	16.9 ± 8.9<br>*39.8 ± 12.4	-6.84
**P8** (BT)	55.4 ± 4.6	-7.34
**P9** (BDD)	44.2 ± 30	-7.21
**P10** (NDI)	52.1 ± 1.2	-7.45

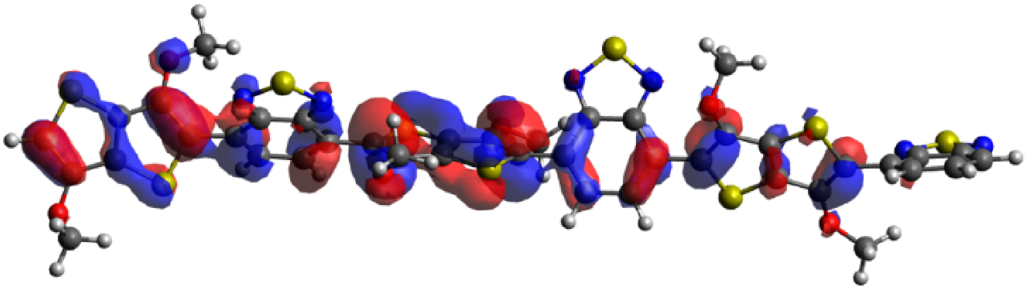

HOMO energy level of **P8** (BT).

S7 References

1. R. Po, G. Bianchi, C. Carbonera and A. Pellegrino, *Macromolecules*, 2015, **48**, 453-461.
2. G. L. Gibson, D. Gao, A. A. Jahnke, J. Sun, A. J. Tilley and D. S. Seferos, *Journal of Materials Chemistry A*, 2014, **2**, 14468-14480.
3. B. Ding, I.-Y. Jo, H. Yu, J. H. Kim, A. V. Marsh, E. Gutiérrez-Fernández, N. Ramos, C. L. Rapley, M. Rimmele, Q. He, J. Martín, N. Gasparini, J. Nelson, M.-H. Yoon and M. Heeney, *Chem. Mater.*, 2023, **35**, 3290-3299.
4. B. Ding, G. Kim, Y. Kim, F. D. Eisner, E. Gutiérrez-Fernández, J. Martín, M.-H. Yoon and M. Heeney, *Angew. Chem., Int. Ed.*, 2021, **60**, 19679-19684.
5. B. Ding, V. Le, H. Yu, G. Wu, A. V. Marsh, E. Gutiérrez-Fernández, N. Ramos, M. Rimmele, J. Martín, J. Nelson, A. F. Paterson and M. Heeney, *Adv. Electron. Mater.*, **n/a**, 2300580.
6. X. Luo, H. Shen, K. Perera, D. T. Tran, B. W. Boudouris and J. Mei, *ACS Macro Lett.*, 2021, **10**, 1061-1067.
7. R. Halaksa, J. H. Kim, K. J. Thorley, P. A. Gilhooly-Finn, H. Ahn, A. Savva, M.-H. Yoon and C. B. Nielsen, *Angew. Chem., Int. Ed.*, 2023, **62**, e202304390.
8. I. McCulloch, M. Heeney, C. Bailey, K. Genevicius, I. MacDonald, M. Shkunov, D. Sparrowe, S. Tierney, R. Wagner, W. Zhang, M. L. Chabinyc, R. J. Kline, M. D. McGehee and M. F. Toney, *Nat. Mater.*, 2006, **5**, 328-333.
9. R. K. Hallani, B. D. Paulsen, A. J. Petty, II, R. Sheelamanthula, M. Moser, K. J. Thorley, W. Sohn, R. B. Rashid, A. Savva, S. Moro, J. P. Parker, O. Drury, M. Alsufyani, M. Neophytou, J. Kosco, S. Inal, G. Costantini, J. Rivnay and I. McCulloch, *J. Am. Chem. Soc.*, 2021, **143**, 11007-11018.

10. A. Giovannitti, C. B. Nielsen, D.-T. Sbircea, S. Inal, M. Donahue, M. R. Niazi, D. A. Hanifi, A. Amassian, G. G. Malliaras, J. Rivnay and I. McCulloch, *Nat. Commun.*, 2016, **7**, 13066.
11. C. K. Song, B. J. Eckstein, T. L. D. Tam, L. Trahey and T. J. Marks, *ACS Appl. Mater. Interfaces*, 2014, **6**, 19347-19354.
12. A. Giovannitti, C. B. Nielsen, J. Rivnay, M. Kirkus, D. J. Harkin, A. J. P. White, H. Sirringhaus, G. G. Malliaras and I. McCulloch, *Adv. Funct. Mater.*, 2016, **26**, 514-523.
13. C. B. Nielsen, A. Giovannitti, D.-T. Sbircea, E. Bandiello, M. R. Niazi, D. A. Hanifi, M. Sessolo, A. Amassian, G. G. Malliaras, J. Rivnay and I. McCulloch, *J. Am. Chem. Soc.*, 2016, **138**, 10252-10259.
14. R. Kroon, D. Kiefer, D. Stegerer, L. Yu, M. Sommer and C. Müller, *Advanced Materials*, 2017, **29**, 1700930.
15. M. Moser, T. C. Hidalgo, J. Surgailis, J. Gladisch, S. Ghosh, R. Sheelamanthula, Q. Thiburce, A. Giovannitti, A. Salleo, N. Gasparini, A. Wadsworth, I. Zozoulenko, M. Berggren, E. Stavrinidou, S. Inal and I. McCulloch, *Adv. Mater.*, 2020, **32**, 2002748.
16. A. Giovannitti, D.-T. Sbircea, S. Inal, C. B. Nielsen, E. Bandiello, D. A. Hanifi, M. Sessolo, G. G. Malliaras, I. McCulloch and J. Rivnay, *PNAS*, 2016, **113**, 12017-12022.
17. S. Griggs, A. Marks, D. Meli, G. Rebetez, O. Bardagot, B. D. Paulsen, H. Chen, K. Weaver, M. I. Nugraha, E. A. Schafer, J. Tropp, C. M. Aitchison, T. D. Anthopoulos, N. Banerji, J. Rivnay and I. McCulloch, *Nat. Commun.*, 2022, **13**, 7964.
18. L. Q. Flagg, C. G. Bischak, J. W. Onorato, R. B. Rashid, C. K. Luscombe and D. S. Ginger, *J. Am. Chem. Soc.*, 2019, **141**, 4345-4354.
19. J. W. Onorato, Z. Wang, Y. Sun, C. Nowak, L. Q. Flagg, R. Li, B. X. Dong, L. J. Richter, F. A. Escobedo, P. F. Nealey, S. N. Patel and C. K. Luscombe, *J. Mater. Chem. A*, 2021, **9**, 21410-21423.

20. D. G. Harman, R. Gorkin, L. Stevens, B. Thompson, K. Wagner, B. Weng, J. H. Y. Chung, M. in het Panhuis and G. G. Wallace, *Acta Biomater.*, 2015, **14**, 33-42.
21. S. Inal, G. G. Malliaras and J. Rivnay, *Nature Communications*, 2017, **8**, 1767.
22. S.-M. Kim, C.-H. Kim, Y. Kim, N. Kim, W.-J. Lee, E.-H. Lee, D. Kim, S. Park, K. Lee, J. Rivnay and M.-H. Yoon, *Nat. Commun.*, 2018, **9**, 3858.
23. J. Rivnay, S. Inal, B. A. Collins, M. Sessolo, E. Stavrinidou, X. Strakosas, C. Tassone, D. M. Delongchamp and G. G. Malliaras, *Nature Communications*, 2016, **7**, 11287.
24. L. Lindell, A. Burquel, F. L. E. Jakobsson, V. Lemaur, M. Berggren, R. Lazzaroni, J. Cornil, W. R. Salaneck and X. Crispin, *Chem. Mater.*, 2006, **18**, 4246-4252.
25. A. I. Hofmann, W. T. T. Smaal, M. Mumtaz, D. Katsigiannopoulos, C. Brochon, F. Schütze, O. R. Hild, E. Cloutet and G. Hadziioannou, *Angew. Chem., Int. Ed.*, 2015, **54**, 8506-8510.
26. S. Inal, J. Rivnay, A. I. Hofmann, I. Uguz, M. Mumtaz, D. Katsigiannopoulos, C. Brochon, E. Cloutet, G. Hadziioannou and G. G. Malliaras, *J. Polym. Sci., Part B: Polym. Phys.*, 2016, **54**, 147-151.
27. R. Meziane, J.-P. Bonnet, M. Courty, K. Djellab and M. Armand, *Electrochim. Acta*, 2011, **57**, 14-19.
28. L. Porcarelli, A. S. Shaplov, M. Salsamendi, J. R. Nair, Y. S. Vygodskii, D. Mecerreyes and C. Gerbaldi, *ACS Appl. Mater. Interfaces*, 2016, **8**, 10350-10359.
29. A. S. Shaplov, P. S. Vlasov, M. Armand, E. I. Lozinskaya, D. O. Ponkratov, I. A. Malyshkina, F. Vidal, O. V. Okatova, G. M. Pavlov, C. Wandrey, I. A. Godovikov and Y. S. Vygodskii, *Polym. Chem.*, 2011, **2**, 2609-2618.
30. O. V. Dolomanov, L. J. Bourhis, R. J. Gildea, J. A. K. Howard and H. Puschmann, *J. Appl. Crystallogr.*, 2009, **42**, 339-341.
31. M. A. Viswamitra, *J. Chem. Phys.*, 2004, **37**, 1408-1414.
